# Schnurri-3 drives tumor growth and invasion in cancer cells expressing interleukin-13 receptor alpha 2

**DOI:** 10.1038/s41419-023-06255-4

**Published:** 2023-11-14

**Authors:** Rubén A. Bartolomé, Ángela Martín-Regalado, Laura Pintado-Berninches, Javier Robles, Mª Ángeles Ramírez-González, Issam Boukich, Pilar Sanchez-Gómez, Irina V. Balyasnikova, J. Ignacio Casal

**Affiliations:** 1 Department of Molecular Biomedicine, Centro de Investigaciones Biológicas (CIB-CSIC), Ramiro de Maeztu 9, 28040 Madrid, Spain; 2 Universidad Autónoma de Madrid. Cantoblanco, Madrid, Spain; 3 Protein Alternatives SL. Tres Cantos, Madrid, Spain; 4 Unidad Funcional de Investigación en Enfermedades Crónicas. Instituto de Salud Carlos III, Madrid, Spain; 5 Department of Neurological Surgery, Northwestern University, Chicago, IL USA; 6 Northwestern Medicine Malnati Brain Tumor Institute of the Lurie Comprehensive Cancer Center, Feinberg School of Medicine, Northwestern University, Chicago, IL USA

**Keywords:** CNS cancer, Colorectal cancer, Cell signalling, Cell invasion

## Abstract

Interleukin 13 receptor alpha 2 (IL13Rα2) is a relevant therapeutic target in glioblastoma (GBM) and other tumors associated with tumor growth and invasion. In a previous study, we demonstrated that protein tyrosine phosphatase 1B (PTP1B) is a key mediator of the IL-13/IL13Rα2 signaling pathway. PTP1B regulates cancer cell invasion through Src activation. However, PTP1B/Src downstream signaling mechanisms that modulate the invasion process remain unclear. In the present research, we have characterized the PTP1B interactome and the PTP1B-associated phosphoproteome after IL-13 treatment, in different cellular contexts, using proteomic strategies. PTP1B was associated with proteins involved in signal transduction, vesicle transport, and with multiple proteins from the NF-κB signaling pathway, including Tenascin-C (TNC). PTP1B participated with NF-κB in TNC-mediated proliferation and invasion. Analysis of the phosphorylation patterns obtained after PTP1B activation with IL-13 showed increased phosphorylation of the transcription factor Schnurri-3 (SHN3), a reported competitor of NF-κB. SHN3 silencing caused a potent inhibition in cell invasion and proliferation, associated with a down-regulation of the Wnt/β-catenin pathway, an extensive decline of MMP9 expression and the subsequent inhibition of tumor growth and metastasis in mouse models. Regarding clinical value, high expression of SHN3 was associated with poor survival in GBM, showing a significant correlation with the classical and mesenchymal subtypes. In CRC, SHN3 expression showed a preferential association with the mesenchymal subtypes CMS4 and CRIS-B. Moreover, SHN3 expression strongly correlated with IL13Rα2 and MMP9-associated poor prognosis in different cancers. In conclusion, we have uncovered the participation of SNH3 in the IL-13/IL13Rα2/PTP1B pathway to promote tumor growth and invasion. These findings support a potential therapeutic value for SHN3.

## Introduction

PTP1B, also called PTPN1, is an ubiquitous tyrosine phosphatase that modulates a wide array of physiological processes, such as glucose metabolism, vesicle trafficking, endoplasmic reticulum stress, apoptosis, differentiation, and cell signaling [1]. According to these multiple roles, PTP1B alterations have been associated with several conditions, such as diabetes, obesity, cardiovascular diseases and cancer [2–4], supporting a central position for PTP1B at the crossroads of inflammation and metabolism. PTP1B overexpression is associated with the expression of TNFα [5], and is partially regulated by the transactivation of NF-κB [5], which plays a central role in promoting inflammation by inducing the expression of some cytokines and chemokines [6]. We have recently identified PTP1B as a critical mediator in the interleukin 13 (IL-13)/IL-13 receptor α2 (IL13Rα2) pathway involved in colorectal cancer (CRC), ovarian cancer and glioblastoma (GBM) progression [7]. IL-13 is a suppressor of type 1 and type 17-associated inflammation [8], although IL-13 drives equally important type 2 inflammation [9]. IL-13 is also a powerful in vivo regulator of tissue remodeling and fibrosis in airway hyperresponsiveness and asthma [10].

PTP1B has been reported to function as a tumor promoter by inducing cell proliferation, invasion, and metastasis [7, 11, 12]. PTP1B is overexpressed in breast, colorectal, ovarian, and prostate cancer [12–14] and frequently correlates with poor prognosis of patients at advanced stages [14–17]. After IL-13 addition, PTP1B associates with IL13Rα2 Tyr_369_ to promote Src activation [7], which in turn activates PI3K through FAM120A, a scaffold protein [18] promoting cell adhesion, migration, invasion, proliferation and survival in tumor cells [18, 19]. PTP1B inhibitors (i.e. claramine) blocked IL-13/IL13Rα2-mediated invasion and metastasis development in CRC, GBM and ovarian cells [7]. PTP1B is anchored in stretchable regions of the endoplasmic reticulum located in the cell periphery to facilitate the association with the target (i.e. IL13Rα2) [20]. In any case, IL-13 binding provokes the internalization of the IL13Rα2 receptor [7]. Therefore, PTP1B regulates biological processes using different cellular localization or a different set of adaptor proteins.

A deeper characterization of the PTP1B molecular networks should contribute to elucidating the molecular connections between IL-13, IL13Rα2, PTP1B, and other mediators in inflammation and cancer progression. Previous analyses of PTP1B functional interactions focused only on direct substrates [21]. Here, we aimed to identify a wide array of interactors using complementary proteomic strategies. In addition, elucidating the phosphorylation alterations ultimately regulated by PTP1B after treatment with IL-13 should identify novel signaling pathways, providing us with additional insights into the role of PTP1B in cancer invasion and inflammation. Here, we identified a close association of PTP1B with different proteins of the NF-κB signaling pathway together with a novel mechanism involving the activation of the transcription factor Schnurri-3 (SHN3), to promote cancer cell growth and invasion by activating the Wnt-β-catenin pathway and the expression of matrix metalloprotease 9 (MMP9). Remarkably, specific inhibition of SHN3 caused a substantial regression of tumor growth and metastasis in mouse models. We identified a correlation between SHN3 expression and poor prognosis in GBM and other tumors. Understanding the mechanistic basis of IL13/IL13Rα2-promoted invasion may have implications for treating GBM and other IL13Rα2-positive tumors.

## Results

### The PTP1B protein interaction network reveals a significant association with proteins from the NF-κB signaling pathway

**Fig. 1:**
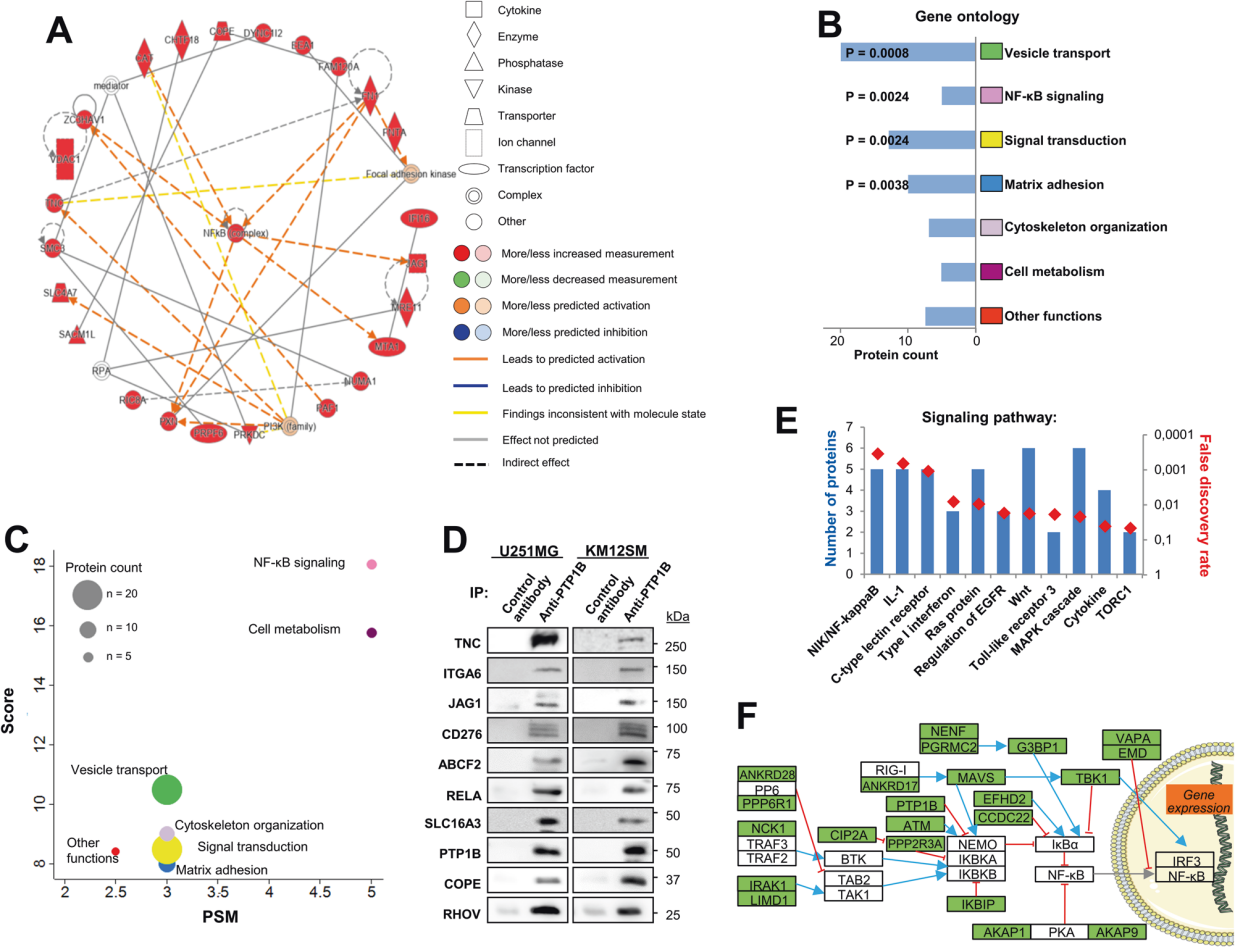
Immunoprecipitation and Bio-ID analyses of PTP1B in U251 GBM cells. **A** NF-κB complex showed the highest score functional network according to IPA analysis of the PTP1B interactors identified by immunoprecipitation and mass spectrometry. **B** GO analysis corresponding to biological processes associated with PTP1B. False discovery rates lower than 0.05 are indicated. **C** Associated protein functions according to peptide number and identification score. **D** Confirmation of PTP1B-associated proteins using Western blot analysis in U251 GBM and KM12SM CRC cells. Data are representative of three independent experiments. **E** Significant signaling pathways identified in the PTP1B Bio-ID interactome by Gene ontology (Biological Process Annotation) showing the number of identified proteins in the pathway (left axis) and the false discovery rates (red diamonds, right axis). **F** Data from the literature and KEGG pathway database were used to highlight those proteins involved in the NF-κB signaling pathway. Green-shaded proteins were detected by mass spectrometry after PTP1B Bio-ID in U251 cells. Data are representative of three independent experiments.

The PTP1B protein interaction network was investigated using immunoprecipitation (IP) in the U251 GBM cell line and BioID assays in HEK293 Flip-In PTP1B cells followed by mass spectrometry. U251 cells express IL13Rα2 and PTP1B at high level [7]. Using IP, we identified 56 PTP1B-specific interacting proteins (Supplementary Table S1). Ingenuity Pathway Analysis (IPA) revealed that the top protein interactome networks were centered in the NF-κB pathway complex and its p65 subunit, also known as RELA (Fig. 1A). RELA was the main interactor of PTP1B according to the number of identified peptides. Other proteins of the NF-κB pathway present in the interactome were NFKBIB, NFKBIE, NEMO (IKBKG), CSNK2, JAG1, the extracellular matrix protein TNC and fibronectin (FN) (Supplementary Table S1). To note that NF-κB signaling plays a key role in the mesenchymal differentiation of GBM [22]. Gene ontology (GO) analysis clustered PTP1B-associated proteins in vesicle transport, NF-κB signaling, signal transduction and matrix adhesion (Fig. 1B). When protein families associated with PTP1B were represented according to the number of identified peptides *vs* the score, the results indicated NF-κB family as the proteins with the highest score and peptide number (Fig. 1C). The association of PTP1B with NF-κB signaling proteins (TNC, JAG1, RELA), adhesion molecules (ITGA6), vesicle transport proteins (ABCF2, RHOV and COPE), and transporters like SLC16A3 was confirmed by Western blot in U251 and KM12SM cells (Fig. 1D).

To complement the IP results, we carried out a proximity biotinylation BioID assay coupled with mass spectrometry in HEK293 Flip-In PTP1B cells. We identified 218 high-confidence PTP1B-interacting proteins (Supplementary Table S2). GO analysis classified the interacting proteins in vesicle transport, regulation of signal transduction, cell cycle, and cytoskeleton organization (Supplementary Fig. S1A). PTP1B interacting proteins were involved in transport routes from the endoplasmic reticulum to the Golgi apparatus, exocytosis, endocytosis, recycling endosomes transport, etc., as well as membrane organization according to the RER location of PTP1B (Supplementary Fig. S1B). Numerous signaling pathways, including MAPK and Wnt signaling, were associated with the PTP1B interactome, with NF-κB signaling reaching the highest significance (Fig. 1E). A representation of the identified proteins using KEGG templates showed multiple regulators of NF-κB activation, including proteins regulating IκBα, which keeps NF-κB in cytosol, as TBK1, EFHD2 or CCDC22 (Fig. 1F). Also, we identified pathways regulating the IKK complex, the main activator of NF-κB by inducing the degradation of IκBα, the cytokine-activated IRAK1 or NCK1, as well as IKBIP, ATM or MAVS. Similar to the IP results, protein networks with the highest score after IPA analysis were centered in NF-κB and ERK1/2 (Supplementary Fig. S1C). Overall, these results indicate a good correlation between BioID and IP results, a significant role of PTP1B in vesicle transport, and a relevant presence of NF-κB-signaling proteins in the interactome of PTP1B.

### Tenascin promotes cell adhesion and invasion but inhibits cancer cell proliferation

**Fig. 2:**
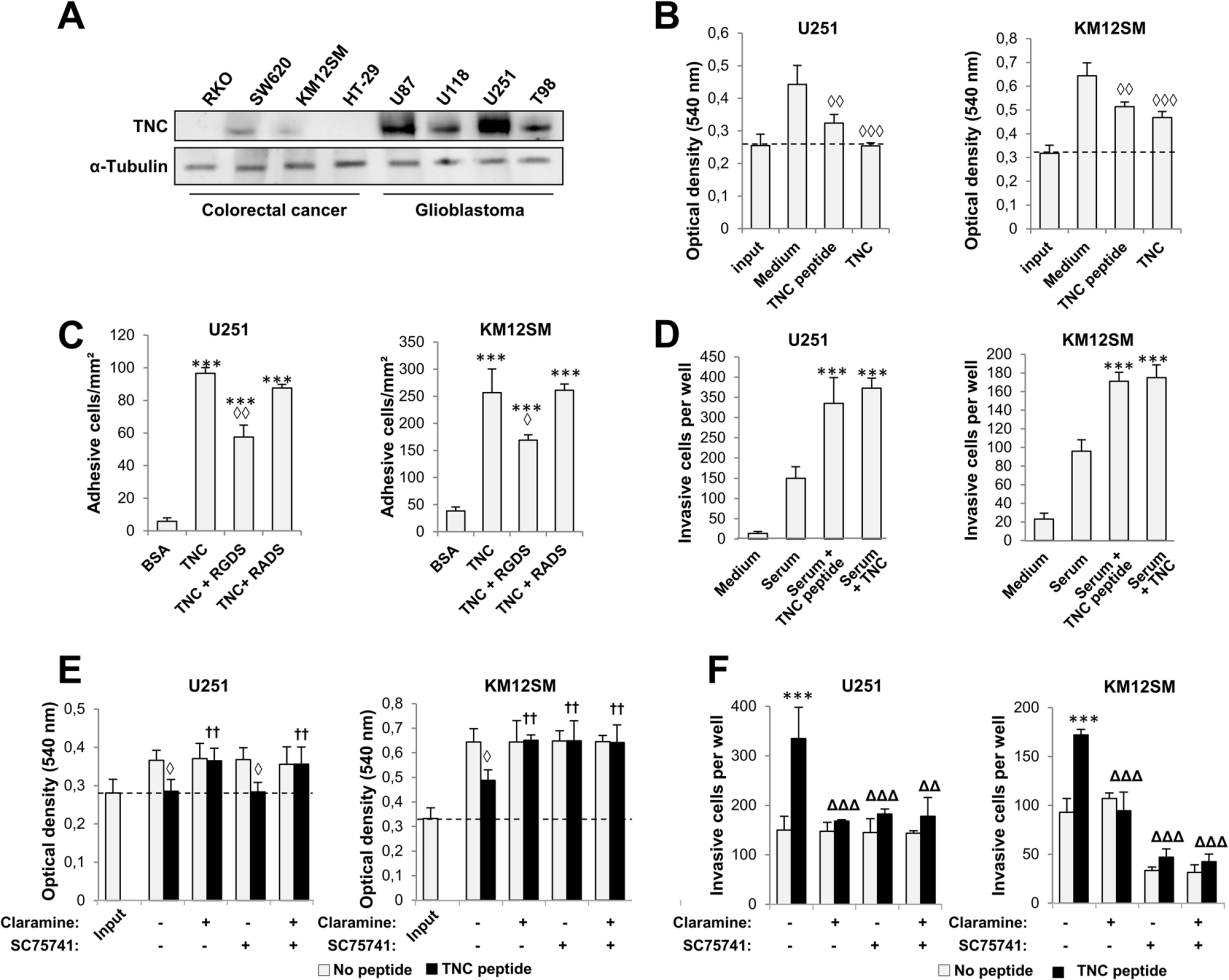
Tenascin-C promotes cancer cell invasion and inhibits cell proliferation in a PTP1B and NF-κB-dependent way. **A** Expression of TNC in the indicated human CRC and GBM cell lines by Western blot analysis. As a loading control, we used anti-α-Tubulin. **B** Cells were subjected to proliferation assays in the presence of TNC or a 9-aa peptide containing the RGD motif of TNC. Input refers to cell seeding density. The presence of TNC or the peptide significantly inhibited cell proliferation (◊◊, *p* < 0.01; ◊◊◊, *p* < 0.001). **C** KM12SM and U251 cells were subjected to cell adhesion assays to TNC in the presence of RGDS or RADS peptides. Cell adhesion was significantly increased by the addition of TNC (***p < 0.001) and inhibited by RGDS peptides (◊, *p* < 0.05; ◊◊, *p* < 0.01). **D** Cells were subjected to invasion assays in the presence of TNC or a 9-aa peptide containing the RGD motif of TNC. The presence of TNC or the peptide significantly stimulated cell invasion (***p < 0.001). The indicated cells were subjected to proliferation (**E**) or invasion (**F**) assays in the presence or absence of TNC peptide, and the inhibitors Claramine and SC75741. The presence of TNC peptides significantly inhibited cell proliferation (◊, *p* < 0.05) and enhanced cell invasion (***p < 0.001), whereas the presence of the indicated inhibitors significantly recovered cell proliferation (^††^*p* < 0.01) or inhibited cell invasion (ΔΔ, *p* < 0.01; ΔΔΔ, *p* < 0.001) triggered by TNC peptides. Data are representative of three independent experiments in (**A**–**F**).

Since TNC was only present in the U251 IP but not in the non-cancer HEK293 interactome, and given that TNC expression is up-regulated by NF-κB in the aggressive mesenchymal subtype of GBM [23, 24] and associated with unfavorable prognosis in multiple cancers (Supplementary Fig. S2A, B), we next explored the functional links between TNC, PTP1B and NF-κB. GBM cells showed higher expression of TNC than CRC cells (Fig. 2A). TNC inhibited cell proliferation (Fig. 2B), but promoted cell adhesion and invasion in both cell lines (Fig. 2C, D). Extracellular TNC contains an RGD motif that suggests a potential role in integrin activation. Indeed, the three effects were RGD-dependent, as RGDS peptides caused an inhibition of 33–41% in cell adhesion. The TNC 9-aa RGD peptide and the whole protein induced similar effects in invasion and proliferation. Moreover, the siRNA silencing of integrins in U251 and KM12SM cells indicated a partial involvement of α2 and αV integrins in cellular attachment to TNC (Supplementary Fig. S2C, D). Next, cell lines were treated with PTP1B (claramine) and NF-κB (SC75741) inhibitors to investigate the association of TNC with PTP1B and NF-κB. TNC-mediated inhibition of proliferation was abolished in both cell lines after PTP1B inhibition, while NF-κB inhibition was effective only in colon cancer cells (Fig. 2E). Claramine and SC75741 repressed TNC-promoted invasion in both cell lines (Fig. 2F). It is noteworthy that the combination of both inhibitors did not cause further inhibition of cell invasion. These results indicate that PTP1B, together with NF-κB, regulates the TNC effects on proliferation and invasion in GBM and colon cancer cells.

### Alterations of the PTP1B phosphoproteome after IL-13 treatment

**Fig. 3:**
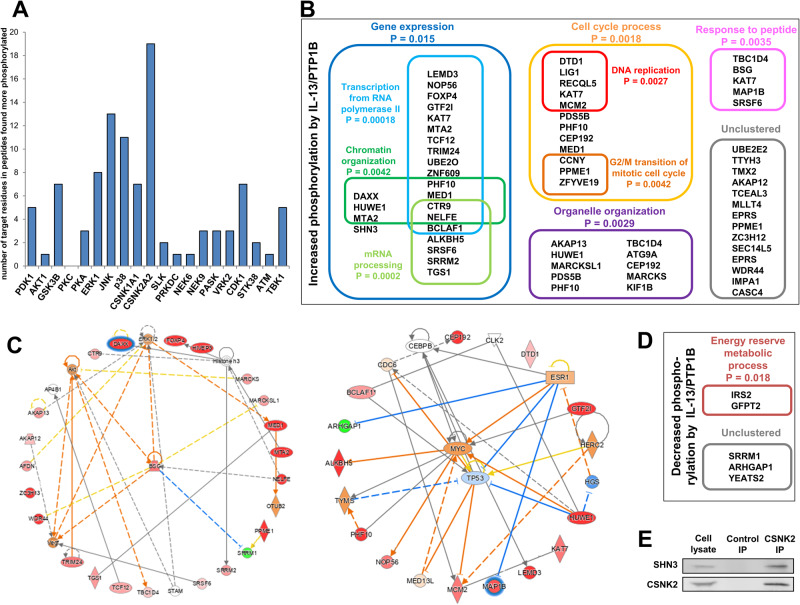
Phosphoproteome alterations mediated by IL-13 and PTP1B. U251 cells were silenced for PTP1B expression, treated with IL-13 and lysed. The extracts were digested, and the peptides analyzed by mass spectrometry. **A** The histogram shows those kinases involved in the phosphorylation of the peptides enhanced by IL-13 through PTP1B according to the GPS 5.0 prediction tool. **B** Identified proteins whose phosphorylation was enhanced by IL-13/PTP1B activities were analyzed by GO using the DAVID database. **C** IPA analysis of IL-13/PTP1B-regulated phosphoproteins. **D** Identified proteins whose phosphorylation was inhibited by IL-13 were analyzed by GO using the DAVID database. **E** CSKN2 IP and Western blot analysis (*n* = 3) of U251 cell lysates showing the association of SHN3 and CSKN2.

To further characterize the role of IL-13/PTP1B in cancer invasion and progression, we analyzed the phosphoproteome of PTP1B-silenced and control cells, treated or not with IL-13 (Supplementary Fig. S3A). Cells were silenced with PTP1B-specific siRNAs as previously described [7], and used for preparation and digestion of protein extracts. After TiO_2_ enrichment of the total peptide content, we identified 2434 phosphopeptides. Amongst them, the phosphorylation status in 59 proteins was altered by IL-13 through the activity of PTP1B (Supplementary Table S3). By using the GPS 5.0 prediction tool [25], we identified those kinases responsible for IL-13/PTP1B-driven phosphorylation events. Among the predicted kinases, the most relevant, by the number of potential substrates identified, was casein kinase 2 (CSNK2A2), related to the Wnt signaling pathway (Fig. 3A). IL-13 treatment of PTP1B-silenced cells led to a significant loss of phosphorylated peptides in the phosphoproteome compared to IL-13-treated control cells. We identified 4 major phosphoprotein clusters according to DAVID analysis (Fig. 3B). The largest cluster (gene expression) was consistent with transcriptional regulation (SHN3), involving proteins related to transcription from RNA polymerase II (FOXP4, GTF2I, TRIM24), chromatin organization (HUWE1) and mRNA processing (SRSF6). The three resting clusters were related to cell cycle, comprising DNA replication (KAT7, MCM2) and G2/M transition of mitotic cell cycle (CCNY, ZFYVE19); organelle organization (AKAP13, MARCKSL-1, ATG9A and KIF1B) and response to peptide (KAT7, MAP1B). After IPA analysis, two phosphoprotein networks centered in basigin (BSG) and, in both p53 and Myc were identified in IL-13-treated cells (Fig. 3C). In addition, we found several proteins differentially phosphorylated by IL-13, such as MARCKS, MARCKSL1, MLLT4 and ARHGAP2 [7, 17], which control actin cytoskeleton remodeling and cell cycle regulation [26] (Supplementary Fig. S3B). Among the few phosphoproteins showing decreased phosphorylation after IL-13 treatment regained after PTP1B-silencing, we identified IRS2 (insulin receptor substrate 2) and GFPT2, related to energy reserve metabolic process (Fig. 3D). In summary, treatment with IL-13 of PTP1B-silenced cells uncovered abundant alterations in transcription factors (i.e. SHN3) related to gene expression regulation.

### SHN3 regulates IL13-promoted cancer cell invasion and proliferation in a NF-κB-independent way

SHN3 showed a phosphorylation at Ser720, in the 720-sLGDEEEPPAFESTK-734 peptide (Supplementary Table S3), located in a disordered region associated with blocking NF-κB activation by TRAF2. According to GPS 5.0, only four kinases can phosphorylate SHN3: LIMK1, HASPIN, GRK7 or CSNK2. CSNK2 was the kinase targeting more substrates affected by IL-13 treatment (Fig. 3A), and was also present in the PTP1B interactome (Supplementary Table S1). To note that the pSer720 peptide matches the consensus sequence [S/T]-x-x-[D/E] reported for CSNK2 substrates. The high frequency of aspartic acid and glutamic acid residues downstream from the Ser agrees with the acidophilic nature of CSNK2 [27]. Moreover, we confirmed the association of SHN3 with CSNK2 by immunoprecipitation (Fig. 3E). All these findings supported the phosphorylation of SHN3 by CSNK2 and led us to investigate the role of SHN3 as a downstream mediator of IL-13/PTP1B.

**Fig. 4:**
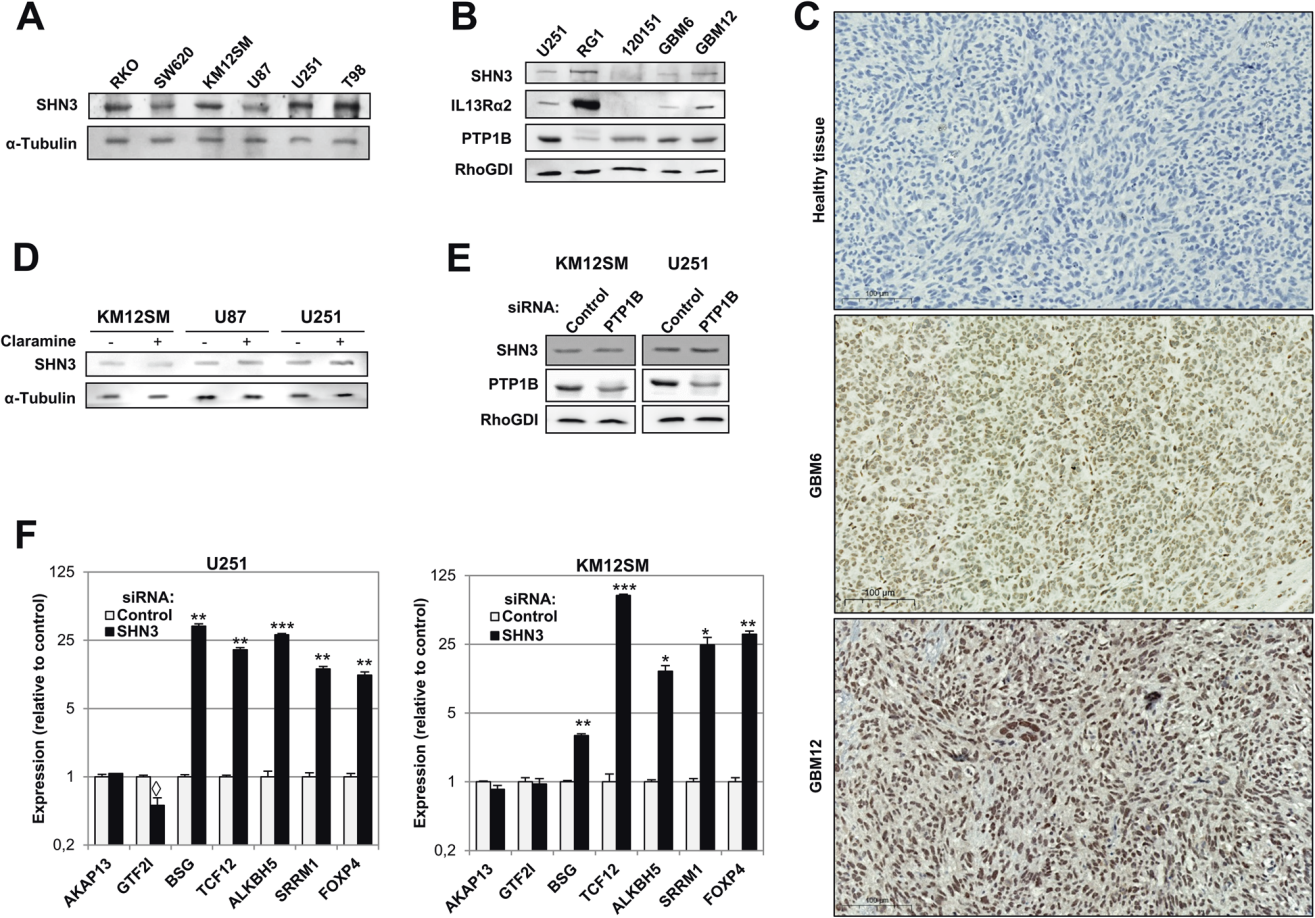
Expression of SNH3 in cancer cell lines and PDXs, and competition with NF-κB. **A** Western blot analysis of SNH3 expression in the indicated CRC and GBM cells. Tubulin was used as a loading control. **B** Western blot analysis of GBM tumor samples showing the expression of SHN3, IL13Rα2, and PTP1B. RhoGDI was used as a loading control. **C** Paraffinated brain tissues of mice inoculated with GBM PDXs 6 and 12 were subjected to immunohistochemistry analysis using an anti-SHN3 antibody. **D** Western blot analysis of SHN3 expression in the indicated cell lines in presence or absence of the PTP1B inhibitor claramine. **E** U251 and KM12SM cells were transfected with control or PTP1B-targeting siRNAs. 48 h after transfection, cells were lysed, and the extracts were analyzed by Western blot to detect the indicated proteins. **F** mRNA from the same transfectants was retrotranscribed and subjected to qPCR to detect alterations in the expression of the indicated NF-κB target genes. Silencing of SNH3 provoked a significant increase (*p < 0.05; **p < 0.01; ***p < 0.001) or decrease (◊, *p* < 0.05) in the expression of the indicated genes. Data are representative of three independent experiments in (**A**–**F**).

SHN3 was similarly expressed in CRC and GBM cells (Fig. 4A). Moreover, SHN3 was expressed in patient-derived xenografts (PDX) GBM samples, as determined by western blot (Fig. 4B) and immunohistochemistry (Fig. 4C). Most samples showed variable expression of SHN3 and IL13Rα2, whereas all expressed PTP1B (Fig. 4B). Interestingly, SHN3 expression correlated with PTP1B and IL13Rα2 expression according to the Chinese Glioma Genome Atlas (CGGA) dataset (Supplementary Fig. S4). Then, we explored the effect of PTP1B-mediated phosphorylation on SHN3 expression. Treatment with the PTP1B inhibitor claramine or siRNA targeting did not cause significant alterations on SHN3 expression (Fig. 4D, E). Therefore, PTP1B does not modulate SHN3 expression, but only its phosphorylation. To study the reported competition between SNH3 and NF-κB [28, 29], we evaluated the effects of SHN3 silencing (Supplementary Fig. S5A) on the expression of NF-κB targets selected using Encode [30] and Swiss Regulon [31] databases among the proteins phosphorylated by IL-13/PTP1B (Supplementary Table S4). Whereas five genes (*BSG, TCF12, ALKBH5, SRRM1* and *FOXP4*) were overexpressed in U251 and KM12SM after SHN3 silencing, a down-regulation of the general transcription factor *GTF2I*, which forms a multiprotein complex at the c-FOS promoter in the AP-1 complex, was observed (Fig. 4F). No transcriptional effects were observed on NF-κB non-target genes (data not shown). These results agree with the previously described binding of SHN3 to κB motifs for gene expression regulation and suggest a potential competition with NF-κB.

**Fig. 5:**
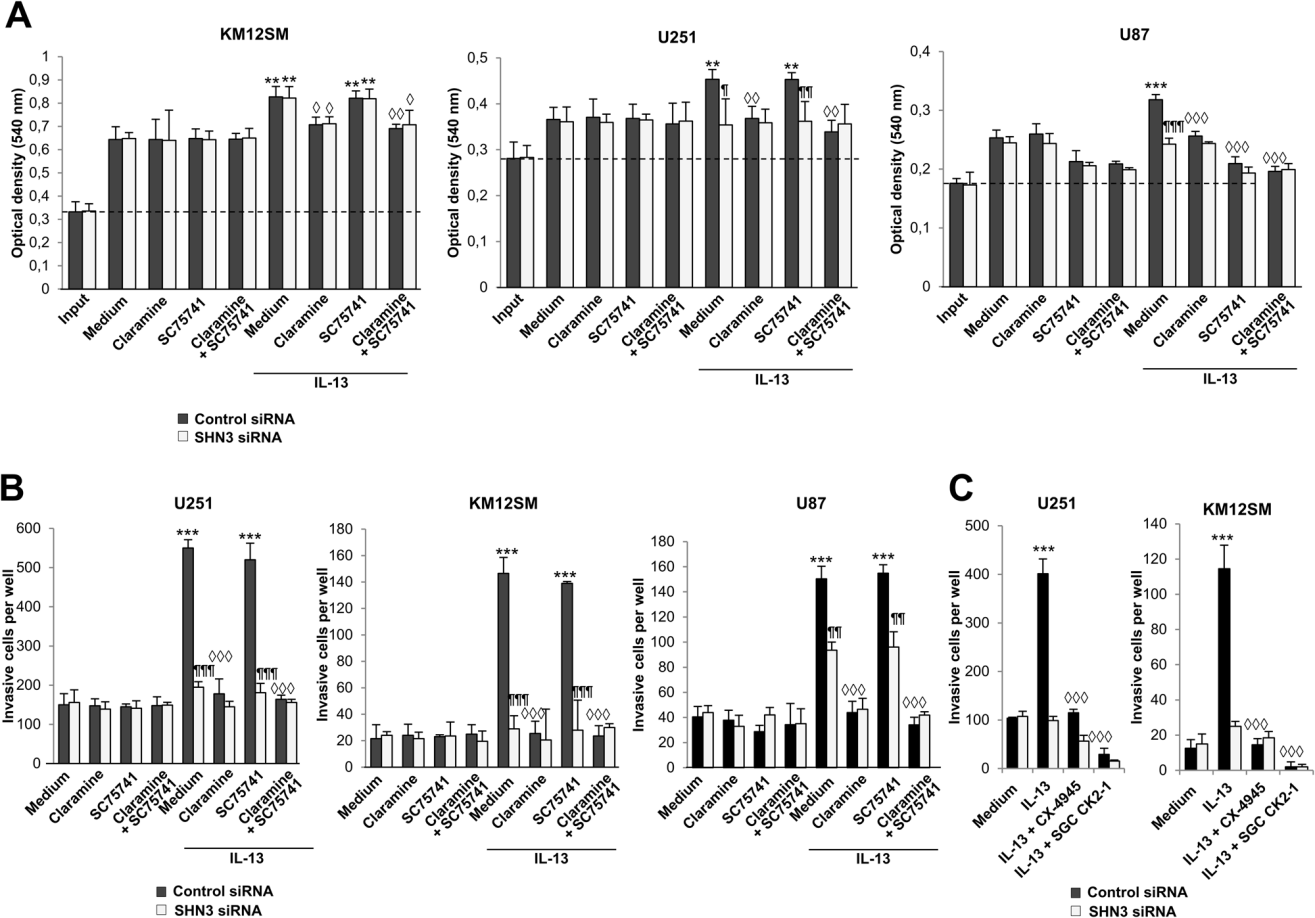
SHN3 is required for IL-13-mediated cell proliferation and invasion. Control or SHN3 silenced KM12SM, U251, and U87 cells were subjected to proliferation (**A**) or invasion (**B**) assays in the presence of IL-13 and the inhibitors Claramine and SC75741. Input refers to cell seeding density. **C** The same transfectant cells were subjected to invasion assays in the presence of IL-13 and the indicated CSNK2 inhibitors. Cell proliferation or invasion was significantly enhanced by the presence of IL-13 (**p < 0.01; ***p < 0.001) and inhibited by SNH3 silencing (^¶^*p* < 0.05; ^¶¶^*p* < 0.01; ^¶¶¶^*p* < 0.001) or the presence of the indicated inhibitors (◊, *p* < 0.05; ◊◊, *p* < 0.01; ◊◊◊, *p* < 0.001). Data are representative of three independent experiments in (**A**–**C**).

Next, we investigated the involvement of SHN3 in cancer invasion and proliferation, before and after IL-13 treatment, in CRC KM12SM cells and GBM U251 and U87 cells. Functional effects were cancer cell-type dependent. SHN3-silencing did not affect proliferation in KM12SM, but decreased IL-13-promoted proliferation in U251 and U87 cells in a PTP1B-dependent fashion and NF-κB-independent for U251 (Fig. 5A). As the combination of SHN3-silencing with PTP1B inhibition was not additive, both proteins should use the same pathway (Fig. 5A). Cell invasion triggered by IL-13 in CRC and GBM cells was equally diminished by SHN3 silencing or PTP1B inhibition. In contrast, NF-κB inhibition did not affect the IL-13 pro-invasive capacity (Fig. 5B). Inhibitors of CSNK2 also abolished the invasion (Fig. 5C), confirming the role of this kinase in SHN3 activation. Therefore, IL-13 pro-invasive activity mediated by SHN3 was PTP1B and CSNK2-dependent but NF-κB-independent.

### SHN3 regulates Wnt signaling and MMP9-mediated invasion

**Fig. 6:**
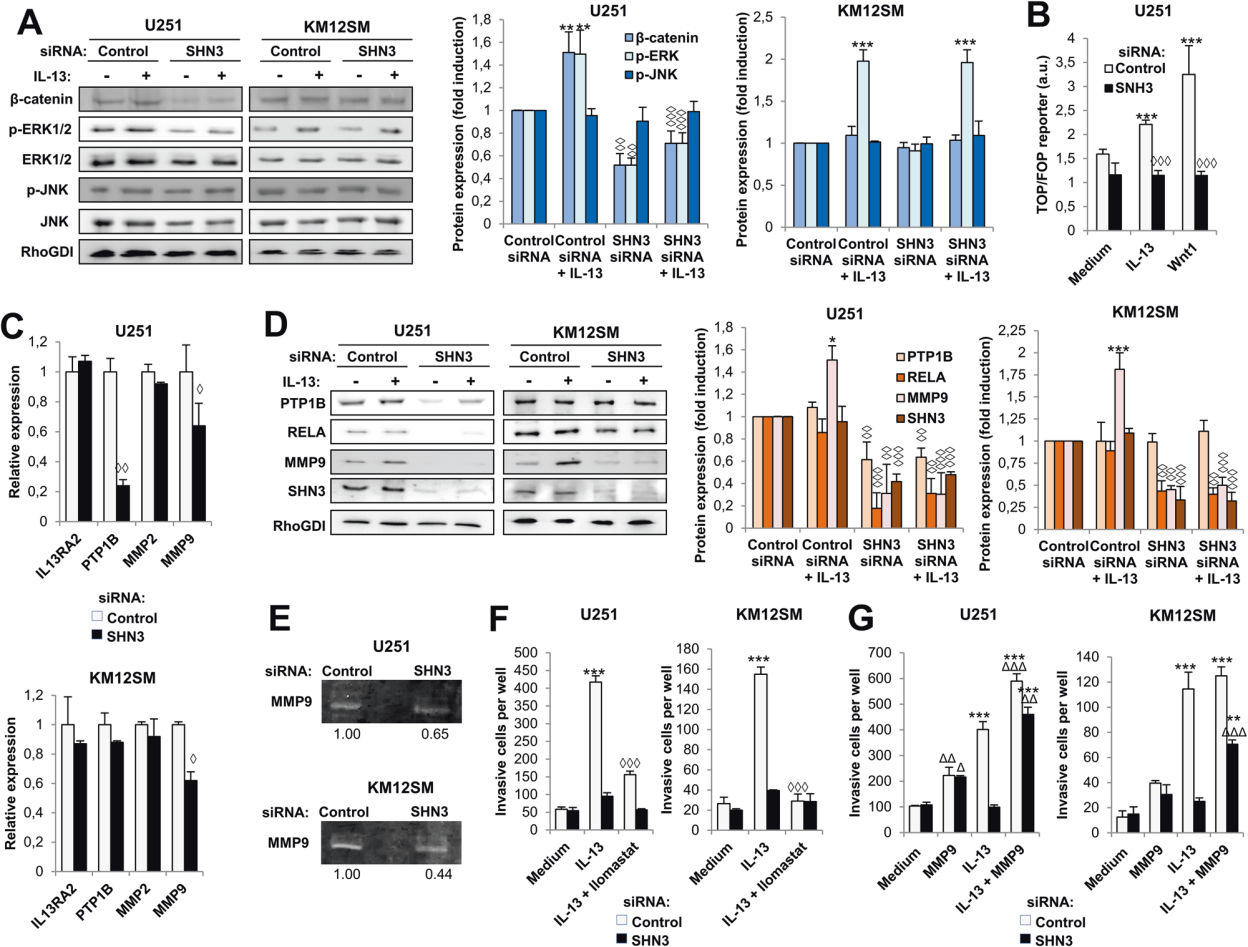
SNH3 silencing inhibits the Wnt/β-catenin pathway and MMP9 expression. **A** Control or SNH3-silenced U251 and KM12SM cells were exposed to IL-13, lysed and the extracts were analyzed by Western blot to detect the indicated (phospho)-proteins. Quantification of the bands is shown in the histograms. The expression of the indicated proteins was significantly enhanced by the presence of IL-13 (*p < 0.05; **p < 0.01) and inhibited by SNH3 silencing (◊, *p* < 0.05; ◊◊, *p* < 0.01; ◊◊◊, *p* < 0.001). **B** Control or SHN3 silenced U251 cells transfected with TOP and FOP vectors were exposed to IL-13 or Wnt1 and lysed, and the luciferase and renilla luminescence of the extracts was quantified. The ratio TOP/FOP was significantly increased by the addition of IL-13 or Wnt1 (***p < 0.001), and this enhancement was significantly inhibited by the silencing of SHN3 (◊◊◊, *p* < 0.001). **C** qPCR of the indicated genes in control or SNH3 silenced cells. The expressions of the indicated genes were significantly reduced upon SNH3 silencing (◊, *p* < 0.05; ◊◊, *p* < 0.01). **D** Western blot analysis and quantification of treated GBM and CRC cells as in (**A**). **E** Gelatinolytic zymography of conditioned media from the same transfectants as in (**A**). The quantification of gelatinolytic activity bands is shown below the strip. **F** The same transfectants were subjected to invasion assays in the presence of IL-13 and/or the MMP9 inhibitor Ilomastat. IL-13-triggered cell invasion (***p < 0.001) was significantly inhibited by Ilomastat (◊◊◊, *p* < 0.001). **G** Invasion assays of the indicated transfectants in the presence of IL-13 and recombinant MMP9. The invasive capacity was significantly enhanced by treatment with IL-13 (***p < 0.001) or MMP9 (Δ, *p* < 0.05; ΔΔ, *p* < 0.01; ΔΔΔ, *p* < 0.001). Data are representative of three independent experiments in (**A**–**G**).

Wnt signaling plays a key role in cell proliferation, invasiveness, and drug resistance associated with the pathophysiology of GBM and CRC [32]. So, we investigated the effects of SHN3 silencing in combination with IL-13 treatment on the expression of β-catenin, pERK1/2 and pJNK activation in U251 and KM12SM cells. Treatment with IL-13 increased the levels of β-catenin, and pERK in U251 control cells. However, SHN3-silencing greatly reduced the levels of β-catenin and the activation of pERK1/2 compared to control cells, in a way partly IL-13 independent (Fig. 6A). pJNK activation was not affected by IL-13 treatment or SHN3 silencing. Interestingly, no alterations were observed in protein expression in KM12SM cells after SHN3 silencing, likely because APC mutations in colon carcinomas prevent β-catenin degradation (Fig. 6A). These differences may explain the distinct effect of SHN3 on proliferation in GBM and CRC cells. To study the effects of SHN3 on Wnt signaling in U251 cells, we used a TOP/FOP reporter luciferase/renilla assay. After using either IL-13 or Wnt1 as activators, knocking down SHN3 significantly decreased the TOP/FOP reporter ratios compared with the mock and control cells, indicating the inhibition of Wnt signaling (Fig. 6B).

Because IL-13/IL13Rα2-promoted invasion is mainly attributed to actions of matrix metalloproteases MMP2 and MMP9 [33, 34], we investigated the effects of SHN3 silencing on gene and protein expression of both metalloproteases and other upstream mediators. MMP9 gene expression, but not MMP2 or IL13Rα2, was significantly reduced in both cell lines (Fig. 6C). PTP1B expression was only reduced in GBM cells. Protein results were confirmed by western blot (Fig. 6D). Although MMP9 expression increased after IL-13 treatment of control cells, SHN3 silencing caused an apparent down-regulation of MMP9 protein expression in both tumors. In addition, RELA, in GMB and CRC, and PTP1B only in GBM cells, were also inhibited by SNH3 silencing in a IL-13 independent way (Fig. 6D). These results indicate that RELA and MMP9 expression were SHN3-dependent. Then, we explored the expression of genes encoding for other NF-κB subunits after SHN3 silencing (Supplementary Fig. S5B). We found a downregulation of RELA and NFKB1 in GBM and CRC cells. In contrast, NFKB2 was upregulated in both cell lines and REL in CRC cells. These results suggest that SHN3 activity might promote a shift in the components of NF-κB, from a heterodimer composed of REL/NFKB2 to another composed of RELA/NFBK1.

Next, we determined MMP9 activity by zymography, using gelatin as a substrate under non-reducing conditions. The gelatinolytic activity of secreted MMP9 was significantly reduced after SHN3 silencing (Fig. 6E). Ilomastat, an MMP9 inhibitor, caused a complete inhibition of IL-13-mediated GBM and CRC cell invasion (Fig. 6F). However, treatment with recombinant MMP9 restored the invasive capacity of SHN3-silenced U251 cells and KM12SM cells (Fig. 6G). Collectively, these results support MMP9 as a critical mediator of SHN3-dependent invasion and SHN3 as a promising target for inhibiting Wnt signaling and MMP9 expression in GBM cells.

### SHN3 inhibition reduces tumor growth and metastasis in IL13Rα2-positive cells

**Fig. 7:**
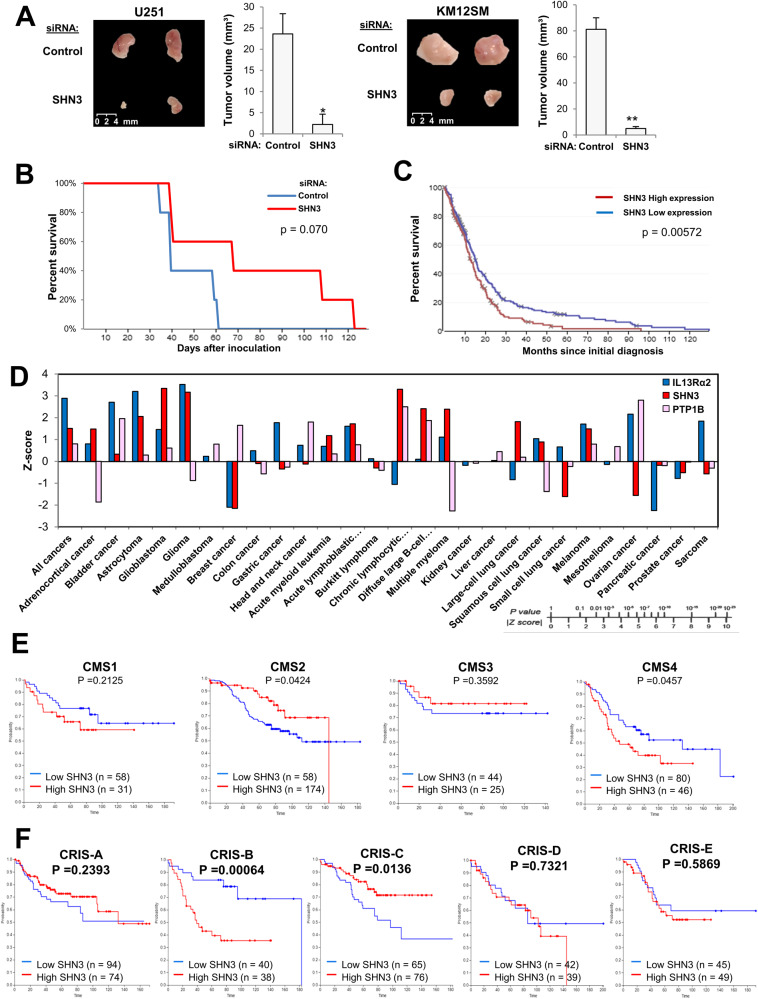
SHN3 silencing inhibits tumor growth and metastasis, and associates with poor prognosis. **A** Mock or SHN3 silenced U251 and KM12SM cells were inoculated in mice subcutaneously. 6 days later, tumors were isolated and measured. The silencing of SNH3 significantly inhibited tumor growth (*p < 0.05; **p < 0.01). **B** Kaplan-Meier representation of survival analysis in mice inoculated in the spleen with control or siRNA-silenced KM12SM cells. **C** SHN3 expression levels correlate with prognosis in glioblastoma patients according to the Betastasis database. **D** Association between expression levels of IL13Rα2, SHN3, and PTP1B and poor overall survival according to the PRECOG web tool by cancer type. **E**, **F** Kaplan-Meyer survival of colorectal cancer patients according to SHN3 mRNA expression levels in each CMS (**E**) or CRIS (**F**) subtype is shown together with the *p*-value of log-rank statistical analyses.

To further evaluate the relevance of SHN3 on tumor growth, we carried out xenograft experiments in Swiss nude mice. U251 and KM12SM cells were siRNA-silenced for SHN3 and inoculated subcutaneously. After 6 days, animals were sacrificed and tumors were removed. In both cases, tumor volumes from SHN3-silenced cells were drastically reduced compared to those from control cells (Fig. 7A). Then, we investigated the capacity of SHN3 to regulate liver metastasis of CRC KM12SM cells. Six Swiss nude mice were inoculated into the spleen either with wild-type or SHN3-silenced KM12SM cells. Mice inoculated with silenced cells showed significantly longer survival than those treated with control cells (Fig. 7B). Post-mortem analysis indicated the presence of liver metastasis, but no colonization in other organs. As no effects on in vitro proliferation were observed in CRC, the xenograft growth inhibition and longer survival of mice inoculated with SHN3 knocked down cells are probably explained by a reduced extracellular matrix degradation. A reduced matrix degradation, associated to the loss of MMP9, resulted in lower tumor growth and poorer angiogenic capacity in different tumors [35–37].

### SHN3 expression is associated with poor prognosis in human cancers

Finally, to explore the clinical relevance of SHN3, we investigated the prognostic value of *SHN3*, together with *IL13Rα2, PTP1B and MMP9* in GBM and other cancers using the Betastasis (REMBRANDT), PRECOG and the Human Protein Atlas (HPA) databases. The analysis of the Betastasis datasets showed a significantly lower survival of GBM patients with tumors overexpressing *SHN3* (log-rank test *P*-value: 0.00563) (Fig. 7C). PRECOG confirmed the association between SHN3 expression and shorter life expectancy in GBM patients, as well as a good correlation between high *SHN3* and *IL13Rα2* expression and poor prognosis in multiple cancers, particularly in tumors of the central nervous system (GBM, astrocytoma), hematological malignancies, melanoma, and some lung cancers, (Fig. 7D). In CRC, using CMS and CRIS molecular classifications [38], we observed a strong association of SHN3 expression with the mesenchymal CMS4 and CRIS-B subtypes, which correspond to CRC patients with the worst outcome (Fig. 7E, F). In GBM, SHN3 expression significantly correlated with the expression of biomarkers corresponding to the mesenchymal subtype [39], including CHI3L1 (aka YKL40) and CD44 (Supplementary Fig. S6A), and with other markers of the classical subpopulation of GBM cells (Supplementary Fig. S6B). In contrast, the study indicated a lack of correlation with markers of the proneural subtype (Supplementary Fig. S6C). According to the SCP393 scRNAseq dataset [40], SHN3 expression was significantly high in mesenchymal and classical GBM subtypes, and low in proneural cells (Supplementary Fig. S7). Finally, using the Human Protein Atlas dataset, overall survival (OS) analyses showed a significant association between high expression of *SHN3* and *MMP9* and poor prognosis in glioma (*p* values: 0.012 and 0.0084, respectively; while *SHN3, IL13Rα2* and *MMP9* expression were also associated with poor OS in the thyroid (*p*-value: 0.011, 0.0097) and renal (*p*-value: 2.8E-9, 1.5E-5) cancers (Supplementary Fig. S8). Taken together, these results indicate an excellent prognostic value for SHN3 in multiple IL13Rα2-positive cancers.

## Discussion

**Fig. 8:**
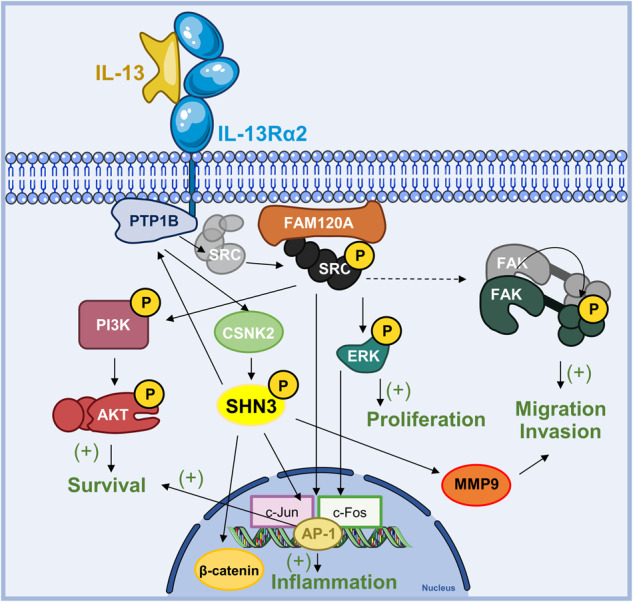
Working model for SHN3 involvement in the IL13/PTP1B signaling pathway. A functional model involving IL13/PTP1B/SHN3/MMP9 during cancer progression in IL13Rα2-positive tumors. IL-13 promotes cancer cell invasion and proliferation in GBM through PTP1B-mediated Src activation, which would then activate CSNK2-mediated SHN3 phosphorylation. In the presence of IL-13, SHN3 regulates Wnt/β-catenin signaling and MMP9 expression, promoting cancer invasion.

An in-depth analysis of the interactome and the phosphoproteome of PTP1B discovered a critical role for the transcription factor SHN3 in regulating IL-13/PTP1B-mediated proliferative and invasive capacities in cancer cells. This conclusion was obtained from the following observations: (1) PTP1B interactome revealed a major association with NF-κB-related proteins, including TNC, (2) treatment with IL-13 promoted SHN3 phosphorylation downstream of PTP1B, likely mediated by CSNK2, (3) SHN3 was essential for IL-13-promoted proliferation and invasion in GBM cells, (4) SHN3 silencing caused inhibition of Wnt signaling and pERK1/2, as well as a substantial decrease in MMP9, PTP1B and RELA expression, (5) in vivo experiments showed that the removal of SHN3 expression inhibited tumor growth and metastasis in IL13Rα2-positive cells and (6) high SHN3 expression is associated with lower overall survival in GBM, CRC and other human cancers. These results support that IL-13/PTP1B-mediated phosphorylation of SHN3 by CSNK2 is a critical player in the crosstalk of Wnt and NF-κB signaling pathways in cancer cells expressing IL13Rα2. To note that CSNK2 is usually overexpressed in GBM patients [41]. A graphical summary of the IL-13/PTP1B signaling cascade is represented in Fig. 8. Our data facilitate a connecting link between the IL-13/PTP1B/Src pathway reported by us [7, 42] and the initially described IL-13/AP-1 pathway [43], as SHN3 has been described to regulate AP-1 activity.

The transcription factor SHN3 (also known as HIVEP3, KBP1, KRC or ZAS3) is a mammalian homologue of *Drosophila Shn*, an essential nuclear cofactor for Decapentaplegic (Dpp) signaling [44]. SHN3 collaborates with other transcription factors, such as SMADs and AP-1, in promoting gene expression [45]. In addition, SHN3 promotes IL-2 and Th2 cytokine (IL-4, IL-13) production during T-cell antigen stimulation, and the expression of AP-1 components such as c-Jun and Jun B [45, 46]. Over the last decade, a crosstalk has been well-established between inflammation and Wnt signaling. Wnt signaling may regulate NF-κB activity and vice versa, in a positive or negative way [47–49]. In this context, SHN3 might be an essential piece of this crosstalk, as knocking-down SHN3 decreased Wnt activity and reduced total β-catenin, suggesting that SHN3 is necessary for β-catenin activation in GBM cells. In this regard, the activation of β-catenin in alveolar cells promotes pulmonary fibrosis [50], in a process that reminds IL-13-mediated fibrosis. The Wnt inhibition, caused by the ablation of SHN3, is accompanied by a decline of RELA and PTP1B expression after SHN3 silencing, together with a reorganization of the NF-κB subunits. These results indicate that SHN3 in GBM cells can regulate NF-κB activity differently. IL-13/IL13Rα2 activities mediated by SHN3 in GBM cells were PTP1B-dependent but NF-κB-independent, suggesting that IL-13/PTP1B/SHN3 might be an alternative or complementary pathway to NF-κB when necessary. Therefore, the IL-13/PTP1B/SHN3 pathway would contribute to the immunosuppressive microenvironment, associated with chronic inflammation and tumor progression observed in GBM and other cancers [51].

IL-13 effects on cancer inflammation and invasion have not been thoroughly investigated, despite IL-13 drives the expression of IL13Rα2, a promising therapeutic target for GBM [52] and other tumors [42]. In this regard, IL-13 causes Th2-derived allergic inflammation and promotes extracellular matrix invasion through AP-1 and the MMP9 collagenase [34]. In this report, we demonstrated the capacity of SHN3 to modulate MMP9 expression and, consequently, xenograft growth and invasion in GBM and CRC cell lines. The MMP9 promoter contains binding motifs for several regulatory transcription factors such as AP-1, specific protein-1 (SP-1) or NF-κB, among others [53]. Since SHN3 participates in the regulation of AP-1 or NF-κB transcriptional activity in IL13Rα2-positive cancer cells, a coordinated action of these transcription factors might explain the regulation of MMP9 expression. Previous reports indicate that the removal of MMP9 expression increases the secretion of IL-4 and IL-13 in the inflammation of respiratory airways [54], suggesting the existence of a self-regulatory loop between IL-13 and MMP9 expression to sustain cancer-enabling inflammation. In this regard, GBM-induced inflammation is caused by an enrichment of inflammatory cytokines and drives the expression of MMP9 in the microenvironment [53].

MMP9 inhibition might explain those results observed in the xenograft and metastatic experiments. Despite SHN3 silencing did not affect in vitro cell proliferation of CRC cells, an inhibition of xenograft tumor growth was observed in both types of tumors, and liver metastasis in CRC. The inhibition of tumor growth could be attributed to a decrease in extracellular matrix degradation resulting from the downregulation of MMP9 expression. This reduction in matrix degradation limits the release of various growth factors, including VEGF, which are essential for tumor growth and the angiogenic processes necessary to supply nutrients [35–37]. In addition, depletion of RELA-mediated nuclear signaling has been reported to delay tumor growth in small cell lung cancer [55]. In summary, the inhibition of MMP9 and RELA might explain the tumor growth reduction and prolonged survival by a combination of reduced matrix degradation, lower invasion and reduced growth factor release (i.e. VEGF).

From a clinical point of view, SHN3 expression analysis showed a robust prognostic value for GBM and other IL13Rα2-positive tumors in multiple databases. NF-κB participates in the up-regulation of several factors identified in the aggressive mesenchymal subtype of GBM (i.e. CHI3L1 and CD44) [22]. The correlation of SHN3 expression with classical and mesenchymal markers in GBM provides additional evidence for supporting the participation of IL-13 in the regulation of NF-κB activation. Interestingly, CHI3L1 (YKL40) works as an IL13Rα2 ligand that promotes cell signaling in melanoma and other diseases [56, 57]. The cooperative or alternative effects of IL-13, CHI3L1 and IL13Rα2 expression on SHN3 activation deserves further investigation. In CRC, high SHN3 expression was associated to poor prognosis only in the most aggressive subtypes, mesenchymal CMS4 and CRIS-B. In addition, we observed a strong correlation of the SHN3 prognostic value with MMP9 and IL13Rα2 in highly aggressive brain tumors, thyroid and renal cancer, which supports a close functional association with these proteins. These correlations likely rely on the capacity of the signaling pathway IL-13/IL13Rα2/PTP1B/SHN3 to trigger cell proliferation and invasion in the more aggressive tumors.

Despite some reported limitations for GBM cell lines [58], they are still essential for elucidating basic mechanisms underlying tumor progression. In this study, our data were retrieved from cancer cell lines with different levels of IL13Rα2 expression and representing different cancer types. Overall, the results support that the mechanisms of IL13Rα2-triggered cancer progression are relatively homogenous, with minor differences likely caused by the different genetic background (i.e. APC mutations in CRC). Taken together, our results reveal that the transcription factor SHN3 is a key mediator of the tumor-promoting IL-13/IL13Rα2/PTP1B signaling pathway. This pathway appears to play a role in adapting cancer cells to chronic inflammation conditions with high NF-κB activation and Th2 cytokine levels, that induce an immunosuppressive microenvironment in the tumor [51], as reported for GBM [59]. We provide evidence of the SHN3 capacity to promote proliferation and invasion by regulating Wnt/β-catenin signaling, ERK1/2 activation and MMP9 expression, as well as regulating PTP1B and RELA expression levels. The combined down-regulation of RELA and MMP9 expression upon SHN3 silencing inhibits tumor growth and cancer invasion, supporting a potential therapeutic value for SHN3 targeting in IL13Rα2-positive tumors.

## Materials and methods

### Cell culture conditions, RNA interference and reagents

Cell lines U251, U87, KM12SM and Flip-In HEK293 (Invitrogen, Carlsbad, CA) were cultured in DMEM (Invitrogen) containing 10% FCS (Invitrogen) and penicillin-streptomycin at 37 °C in a 5% CO_2_ humidified atmosphere. U251 and U87 cells were originally obtained from ATCC (Manassas, VA) [60]. KM12SM cell line originally received from I. Fidler (MD Anderson. USA) was authenticated by short tandem repeat analysis. Cells were tested for mycoplasma routinely. SiRNAs against SHN3 (#1 SASI_HS02_00330334 and #2 SASI_HS02_00330335), PTP1B (SASI_Hs01_00230698) [7], α2 integrin (SASI_Hs01_00123982), αV integrin (SASI_Hs01_00220507) and a control siRNA were obtained from Sigma-Aldrich (St Louis, MO). They were transfected with JetPrime (Polyplus Transfection, Illkirch, France) according to the manufacturer’s instructions. Human recombinant IL-13 and Wnt1 were acquired from Preprotech (London, UK) and used at 10 ng/mL and 500 ng/mL, respectively. Tenascin-C was obtained from Millipore (Burlington, MA) and used at 10 μg/mL. RGDS, RADS and Tenascin-C peptide (ISRRGDMSS) were in-house synthesized using solid-phase chemistry with a Focus XC instrument (AAPPtec, Louisville, KY). For blocking the RGD motif, RGDS and RADS peptides were used at 0.5 mM, whereas Tenascin-C RGD peptide was used at 2 μM in the different assays. PTP1B specific inhibitor Claramine (Sigma-Aldrich) and NF-κB specific inhibitor SC75741 (Selleckchem, Planegg, Germany) were used at 2 and 0.5 μM, respectively. MMP9 inhibitor Ilosmastat (Selleckchem) was used at 2.5 μM. The inhibitors CX-4945 (Peprotech) and SGC CK2-1 (Tocris Bioscience, Bristol, UK) of CSNK2 were used at 5 μM and 2 μM, respectively. Recombinant human active MMP9 was purchased from Calbiochem (San Diego, CA) and used at 10 ng/mL. For a complete antibody list see supplementary information.

### Immunoprecipitation and mass spectrometry

For proteomic analysis, 1 mg of cell lysates were immunoprecipitated (IP) using anti-PTP1B (#sc-133115, SCBT, Dallas, TX) or an irrelevant control antibody as previously described [18]. IP proteins were resolved by SDS-PAGE and divided into three slices for in-gel digestion with trypsin. Mass spectrometry analysis was carried out as previously described [18]. Mass spectra data were analyzed with Proteome Discoverer (1.4.0.288) (Thermo Fischer Scientific, Waltham, MA) and searched against the human Uniprot Database using the Sequest search engine as previously described [18]. After statistical comparison using Student’s *t*-test, a fold-change threshold of 3 compared to control antibody immunoprecipitated proteins was fixed for peptide spectral matches (PSM) in significantly different identified proteins. Proteins identified with at least two peptides in each replicate were filtered using the CRAPome resource (https://reprint-apms.org/) for deleting common contaminants and proteins related to unspecific protein-protein interaction (chaperons, ribosomal proteins, proteasome proteins, etc.). Functional annotations of filtered proteins were analyzed using Biological Process (Gene Ontology) in the g:Profiler database (https://biit.cs.ut.ee/gprofiler/gost).

Proximity biotinylation coupled to mass spectrometry (BioID) and phosphorylation analysis methods are described in supplementary information.

### Ingenuity pathway analyses

Functional networks were algorithmically generated based on the connectivity of the identified proteins in the previous proteomics assays after comparison with Ingenuity Pathways Knowledge Base and Fisher’s exact test to determine the probability of association. Only the two most top-scored networks were represented. In the graphical representation of molecular networks, proteins are represented as nodes whose color indicates the degree of up-regulation (red if measured, orange if predicted) or down-regulation (green if measured, blue if predicted). Relationships between nodes are represented by lines indicating activation (orange) or deactivation (blue). Other in silico methods are described in the supplementary information.

### TOP/FOP reporter assays

TOP/FOP reporter assays were performed to assess the activity of Wnt/β-catenin signaling. In brief, 8xTOPFlash and 8xFOPFlash plasmids were provided by Dr. A Muñoz (IIB-CSIC) and transfected in U251 cells. After the different treatments, cells were lysed, and the luciferase activities of Firefly and Renilla were measured using the Dual-Luciferase Reporter Assays System (Promega, Madison, WI), following the manufacturer’s instructions. The firefly luciferase gene in TOPFlash is controlled by a promotor regulated by β-catenin, whereas such promotor is mutated in FOPFlash vector, setting the basal luminescence. Luciferase activity was normalized by transfection efficacy using the activity of Renilla luciferase encoded in the vectors under a constitutive promotor.

### Immunohistochemistry

Paraffinated brain tissues with patient-derived glioma xenografts (PDXs) were recovered from mouse intracranial experiments as previously described [7]. All experiments were approved by the Northwestern University Institutional Animal Care and Use Committee (IACUC). Immunohistochemistry was carried out as previously described [18] using anti-SHN3 (# HPA005728, Sigma-Aldrich) as primary antibody. MACH2 rabbit HRP polymer (#RHRP520, Biocare, Concord, CA) was used as secondary antibody. The reaction was revealed using DAB as chromogen and hematoxylin for counterstaining. Sections stained with the secondary antibody were used as negative controls.

### Xenograft and metastasis experiments

The Ethics Committees of the CSIC and Community of Madrid approved all protocols used in animal experimentation (PROEX 252/15). KM12SM cells were transfected with control or SHN3-targeted siRNAs as described above. After 16 h, 10^6^ cells were inoculated subcutaneously in the flanks of 12 weeks-old female Swiss nude mice (*n* = 6 per condition). After 6 days, subcutaneous tumors were isolated and measured. Mouse metastasis experiments with KM12SM-silenced cells were performed as previously described [61].

### Survival analysis in cancer patients

For prognostic studies, the GSE17538 [62] and GSE39582 [38] datasets, containing 244 and 232 tumor samples, respectively, with clinicopathological descriptions were used for analysis of CRC patient overall survival. The last dataset contains tumor samples classified by molecular subtypes (CMS and CRIS), and were used for overall survival analysis by subtypes. Data were normalized using Bioconductor’s Affymetrix package and transformed into z-scores, as previously described [63].

For GBM, association with prognosis was evaluated using the Betastasis dataset with 349 samples [64]. Samples were divided using the median as preset threshold according to the expression levels of SHN3. The significance of the difference in survival between high and low-expression populations was estimated by the log-rank test. Additionally, survival z-score analyses by cancer type were obtained from PRECOG [65], which provides false-discovery rates for survival scores. Furthermore, in silico analyses by Kaplan-Meier survival of cancer patients according to the protein expression levels of selected genes were obtained from the Human Protein Atlas [66]. Correlations between genes were evaluated using two glioblastoma datasets (CGGA with 388 samples, and TCGA RNA-seq with 156 samples) and visualized using GlioVis web tool (http://gliovis.bioinfo.cnio.es/). Association between GBM subtypes and SHN3 expression in single cell RNA sequencing datasets was evaluated using SCP393 GBM scRNAseq dataset using the Single Cell Portal web tool (https://singlecell.broadinstitute.org/single_cell/study/SCP393). *Z*-scores for SHN3 expression were compared between two cell populations divided by the median of the mesenchymal, classical or proneural meta-module values. Mesenchymal (MES1-like) and proneural (NPC1-like) meta-modules were previously calculated after each signature expression values [40]. Classical meta-module was calculated by the average of the glial (AC-like and OPC-like) meta-modules.

### Statistical analyses

Histograms showed the average value, indicating the standard deviation as error bars. Data with Gaussian distributions were tested for differences in variance with the *F* test, and analyzed by two-way ANOVA, followed by Tukey-Kramer multiple comparison test. Correlations between genes were evaluated using Pearson’s product-moment correlation test. In all analyses the minimum acceptable level of significance was *p* < 0.05. Mouse or patient survival data were analyzed by log-rank test.

### Supplementary information

Supplementary Information

Checklist

Original western blots

## Data Availability

Original data and materials are available upon request.
