# Supplementary material for: Schnurri-3 drives tumor growth and invasion in cancer cells expressing interleukin-13 receptor alpha 2
Source: Cell Death Dis. 2023 Nov 14;14(11):742. doi: 10.1038/s41419-023-06255-4 (PMC10645886; doi:10.1038/s41419-023-06255-4)
Supplement: Supplementary file 1 — Supplementary Information [file 41419_2023_6255_MOESM1_ESM.pdf]

Schnurri-3 drives tumor growth and invasion in cancer cells expressing interleukin-13 receptor alpha 2

Rubén A. Bartolomé[1], Ángela Martín-Regalado[1], Laura Pintado-Berninches[1,2], Javier Robles[1,4], Mª Ángeles Ramírez-González[3], Issam Boukich[1,4], Pilar Sanchez-Gómez[3], Irina V. Balyasnikova[5] and J. Ignacio Casal[1]

1. Department of Molecular Biomedicine. Centro de Investigaciones Biológicas (CIB-CSIC). Ramiro de Maeztu 9. 28040 Madrid, Spain.
2. Universidad Autónoma de Madrid. Cantoblanco. Madrid. Spain
3. Unidad Funcional de Investigación en Enfermedades Crónicas. Instituto de Salud Carlos III. Madrid, Spain
4. Protein Alternatives SL. Tres Cantos. Madrid.
5. Department of Neurological Surgery, Feinberg School of Medicine, Northwestern University, Chicago, IL 60611, USA.

Supplementary methods

Supplementary figures S1-S8

Supplementary tables S1-S4

SUPPLEMENTARY MATERIALS AND METHODS

Antibodies

Antibodies against PTP1B (B-9), α6 integrin (ITGA6, GOH3), α2 integrin (P1E6), αV integrin (H-2), CD276 (F-11), ABCF2 (E-2), RELA (5G8), MMP9 (E-11), MCT-4 (SLC16A3, D-1), JAGGED-1 (JAG1, E-12), RHOV (F-2), β-catenin (E-5), phospho-GSK3β (F-2), GSK3β (E-11) and RhoGDI (G-2) were purchased from Santa Cruz Biotechnology. Antibodies against phospho-ERK1/2 (#9101), ERK1/2 (#4695), phospho-JNK (#9255) and JNK (#9558) were from Cell Signaling Technology. Anti-COPE (267-280) and anti-SHN3 (HPA005728) antibodies were from Sigma-Aldrich. Anti-Tenascin-C (TNC, BC-24) and anti-α-Tubulin (ab21057) were from Thermo Fisher Scientific and Abcam, respectively.

Immunoprecipitation and Western blot analysis

For immunoprecipitation, cells were lysed with protease and phosphatase inhibitors in lysis buffer (1% Igepal, 100 mM NaCl, 2 mM $MgCl_2$, 10% Glycerol in 50 mM Tris-HCl). 1 mg of cell lysate was incubated with anti-PTP1B or control antibodies and Protein G-sepharose beads (Sigma-Aldrich) for 16 h. Immunoprecipitates were washed four times, resuspended in Laemmli buffer, and loaded onto SDS-PAGE gels, which were analyzed by Western blot analysis.

For Western blot, cells were lysed as before and 75 μg of proteins were resolved in SDS-PAGE and transferred to nitrocellulose membranes. Blots were incubated with primary antibodies followed by incubation with HRP-conjugated secondary antibodies (Thermo Fisher Scientific). Bands were visualized with SuperSignal West Pico Chemiluminescent Substrate (Thermo Fisher Scientific) in the Chemidoc Imaging System device (Bio-Rad).

Proximity biotinylation coupled to mass spectrometry (BioID)

PTPN1-pDONR201 (DNASU Plasmid Repository, AZ, USA) plasmid was cloned into the Flp-In T-REx system destination plasmid, pDest-pcDNA5-BirA-Flag-Ct (a gift from Dr. Anne-Claude Gingrass) using Gateway LR Clonase II enzyme mix (Invitrogen). The resultant product was validated by sequencing using the oligonucleotide: 5'-ATCCTGGAGCCACACAATGG-3'. Flip-In T-Rex HEK-273 host cell line was transfected with the generated plasmid and pOG44 using Jet Prime. Flip-In T-Rex HEK-273 cells are tailored for BioID analyses. After recombination, Flp-In T-Rex cells carrying the fusion gene were treated with doxycycline (0.5 μg/mL, Sigma-Aldrich) and biotin (50μM, Sigma-Aldrich) for 24 h. Then, cells were lysed, and biotinylated proteins purified using streptavidin-coupled Sepharose beads (GE Healthcare). Purified biotinylated proteins were

analyzed by mass spectrometry, as before **(Supp. Fig. S1)**. As a control, Flp-In T-Rex HEK-273 cells transfected with pcDNA.5-pDEST-BirA-Flag-Ct were subjected to the same process. Analysis and filtering of identified proteins was carried out using label-free quantification (LFQ) intensities using the SAINT web-server (https://reprint-apms.org/) (1), designed for BioID assays with a Max score > 0.5. Functional annotations and networks were analyzed as before.

Phosphorylation analysis by mass spectrometry

To analyze the phosphoproteome, U251 cells were transfected with siRNAs against PTP1B or control. 48 h after transfection, cells were starved for 3 h and incubated with or without IL-13 (10 ng/mL) for 5 min. Then, cells were lysed as before, and 300 µg of protein extracts were subjected to reduction, alkylation and digestion with trypsin. Phosphopeptide enrichment was performed by affinity chromatography using Titansphere TiO_2 beads (GL Sciences). Phosphopeptides were subjected to mass spectrometry as above. The phophoRS node 3 of Proteome Discoverer was applied to assess the statistical confidence in the phosphorylation site location. Analysis of phosphopeptides was carried out by using peptide-to-spectrum matches (PSM), taking into consideration peptides with missed cleavages and peptides with multiple phosphorylation sites. Only phosphopeptides with at least two PSM per replicate (in at least one condition) were considered in the analysis. A 2.0-fold change cutoff was fixed as significant. Heatmap analysis was performed using the $\log_2$ of fold change with respect to the PSM number average of the four conditions. Functional annotations of the selected proteins were analyzed using Gene Ontology (GOTERM_BP_FAT) in DAVID (https://david.ncifcrf.gov) database, IPA software data mining of the scientific literature. Potential kinases involved in the phosphorylation of the peptides from the selected proteins were predicted using GPS 5.0 website (http://gps.biocuckoo.cn).

Quantitative PCR

RNA was isolated using TRIZOL reagent (Ambion) and retrotranscribed using MMLV-RT (Promega) and random hexamers (Invitrogen). Complementary DNA was subjected to real-time PCR using FastStart Master Mix (Roche) in a LightCycler 480 Real-Timer PCR System (Roche) using specific primers for ALKBH5 (5'-TGTCCGTGTCCTTCTTTAGC-3', 5'-TTCATCAGCAGCATATCCAC-3'), BSG (5'-TCACCTGCTCCTTGAATGAC-3', 5'-CCTTGAACTCCGTTTTCTGG-3'), FOXP4 (5'-ACTGGTTCACCAGGATGTTC-3', 5'-GGTCTCCGCTTCTGATACTC-3'), GTF2I (5'-CGTGCTTTTGTCAATACCAG-3', 5'-TCTACACTCATCCGATTTGC-3'), SRRM1 (5'-AGAAGAACTCCGCCAAGAAG-3', 5'-ACCGTGAACGAGATGAAGAG-3'), TCF12 (5'-TCAACCAAGTCCTTCCTATG-3', 5'-AAATGAGCCTCTCTCTGATG-3'), AKAP13 (5'-AGGGTCATTGGACTCAGAAC-3', 5'-

GGCTGGTTGAACTCAGTATG-3’), IL13RA2 (5’-GGATCATCAGAGAACAAG-3’, 5’-GAATAGGTCCCAAAGGTATG-3’), MMP2 (5’-TGTTGGTGGGAACTCAGAAG-3’, 5’-ACTTGCGGTCATCATCGTAG-3’), MMP9 (5’-ACTTTGACAGCGACAAGAAG-3’, 5’-TCAGTGAAGCGGTACATAGG-3’) and PTP1B (5’-GAAGGAGGACGGTTGTAAGC-3’, 5’-AACAGGGAGGAGAATAAGCG-3’). Each value was adjusted using GAPDH and 18S RNA levels as references.

Identification of transcription factor targets

To identify the potential targets of NF-kB in the set of identified phosphoproteins, we examined two databases: SwissRegulon (https://swissregulon.unibas.ch/sr/swissregulon) and ENCODE (https://maayanlab.cloud/Harmonizome/dataset/ENCODE+Transcription+Factor+Targets), which predict transcription factor binding sites in regulatory regions of analyzed genes.

Cell adhesion, proliferation and invasion assays

For cell adhesion, cells were starved for 3h and labelled with BCECF-AM (Molecular Probes). Then, 6×10^4 cells were added into 96-well plates previously coated with Tenascin-C (10 μg/mL) and incubated for 25 min in the presence or absence of peptides (0.5 mM). Non-adhered cells were removed by washing, whereas bound cells were quantified using POLARstar Galaxy fluorescence analyzer (BMG Labtech).

For proliferation assays, 1×10^4 cells were added on 96-plates previously coated with or without Tenascin-C (10 μg/mL) or TNC peptide (2 μM) and allowed to attach for 2h, followed by 48 h incubation in the presence of 0.5% serum, with or without IL-13 (10 ng/mL), Claramine (2 μM), SC75741 (0.5 μM), CX-4945 (5 μM) and/or SGC CK2-1 (2 μM). Then, 0.6 mg/mL Thyazolyl Blue Tetrazolium Bromide (MTT) (Sigma-Aldrich) was added for 1 h. Cell proliferation was determined by absorbance at 560 nm and compared with the absorbance of cells incubated with MTT for 1 h immediately after the attachment (Input).

For invasion assays, 6×10^4 cells were loaded onto 8 μm pore-sized transwells (Corning) coated with 35 μL of DMEM containing Matrigel (BD Biosciences) at 3 mg/mL with or without Tenascin-C (10 μg/mL) or TNC peptide (2 μM), and incubated for 48 h in presence of medium or serum (4%). For IL-13 triggered invasion assessment, cells loaded onto the Matrigel coated traswells were incubated without serum in the presence or absence of IL-13 (10 ng/mL), active MMP9 (10 ng/mL), Ilomastat (2.5 μM), Claramine (2 μM), SC75741 (0.5 μM), CX-4945 (5 μM) and/or SGC CK2-1 (2 μM). Non-invading cells were removed and migrated cells were fixed with

paraformaldehyde (Sigma-Aldrich) at 4% for 15 min, stained with crystal violet (Sigma-Aldrich) for 30 min and counted under a microscope.

Data from cell adhesion, proliferation and invasion assays were analyzed by one-way ANOVA test followed by Tukey-Kramer multiple comparison test. P values < 0.05 were considered statistically significant.

Zymography

Twenty-four h conditioned media from cancer cells incubated in serum-free medium were concentrated 20 times using Vivaspin 15R tubes (Sartorius). Concentrated media were mix with Laemmli buffer and resolved under non-reducing conditions polyacrylamide gels containing 1% bovine skin gelatin (Sigma-Aldrich). Gels were washed with 2.5% Triton X-100 (Sigma-Aldrich) followed by incubation in reaction buffer (200 mM NaCl, 50 mM Trism 10 mM $CaCl_2$) for 16 h at 37 ºC. Gels were stained with Coomasie Blue (Bio-Rad) and areas of proteolytic activity around 90 kDa were quantified using MultiGauge software (FujiFilm).

References

1. Choi H, Larsen B, Lin ZY, Breitkreutz A, Mellacheruvu D, Fermin D, *et al.* SAINT: probabilistic scoring of affinity purification-mass spectrometry data. Nat Methods 2011;8:70-3

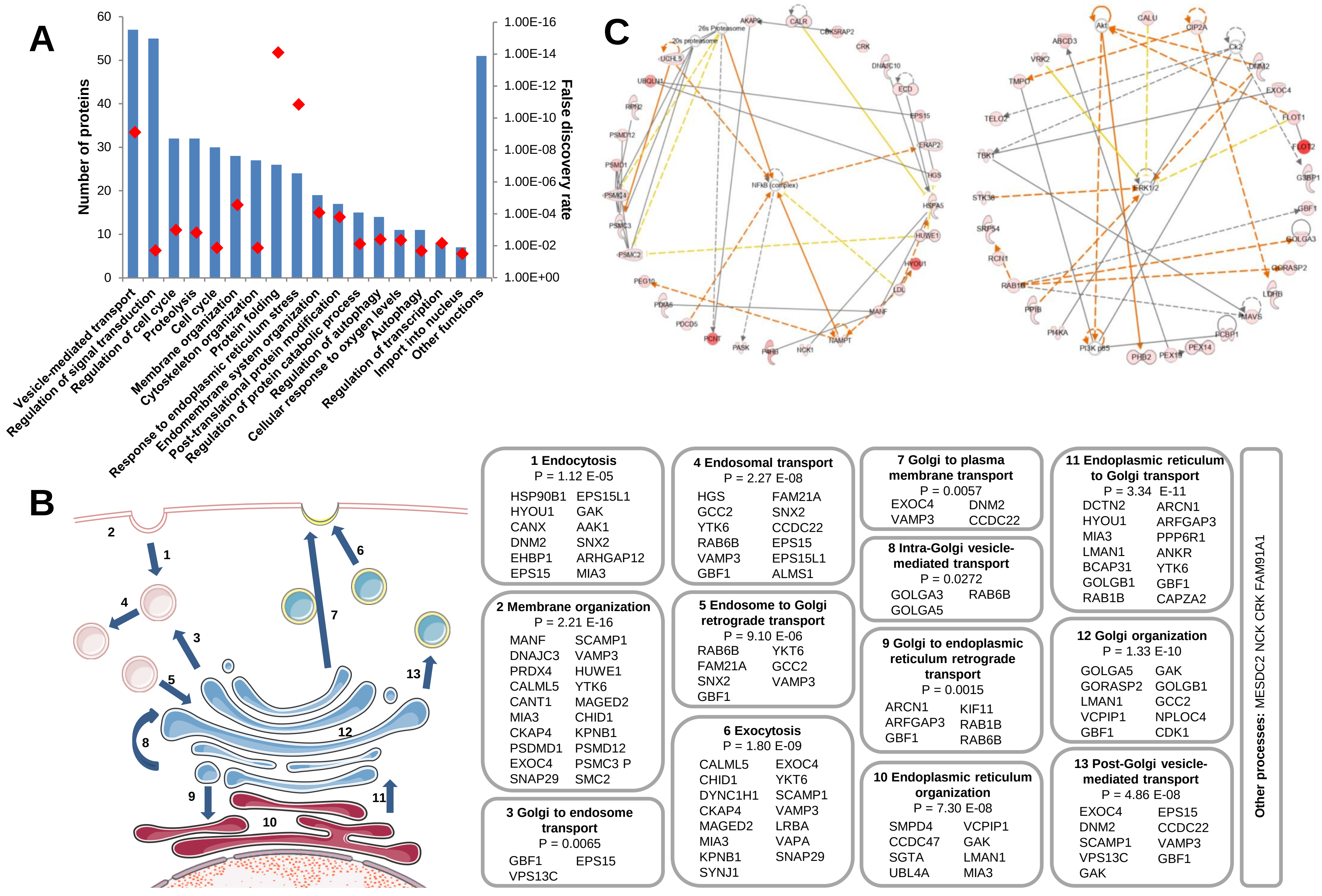

Figure S1. Analysis of PTPN1 interactome by BioID. (A) Gene ontology analysis of statistically significant biological functions according to g:Profiler. (B) Representation of proteins involved in vesicle-mediated transport and organization of membrane systems, identified in the PTP1B interactome. The false discovery rate p values of each subprocess are indicated inside each protein cluster. (C) Highest score functional networks identified by IPA in the PTP1B interactome.

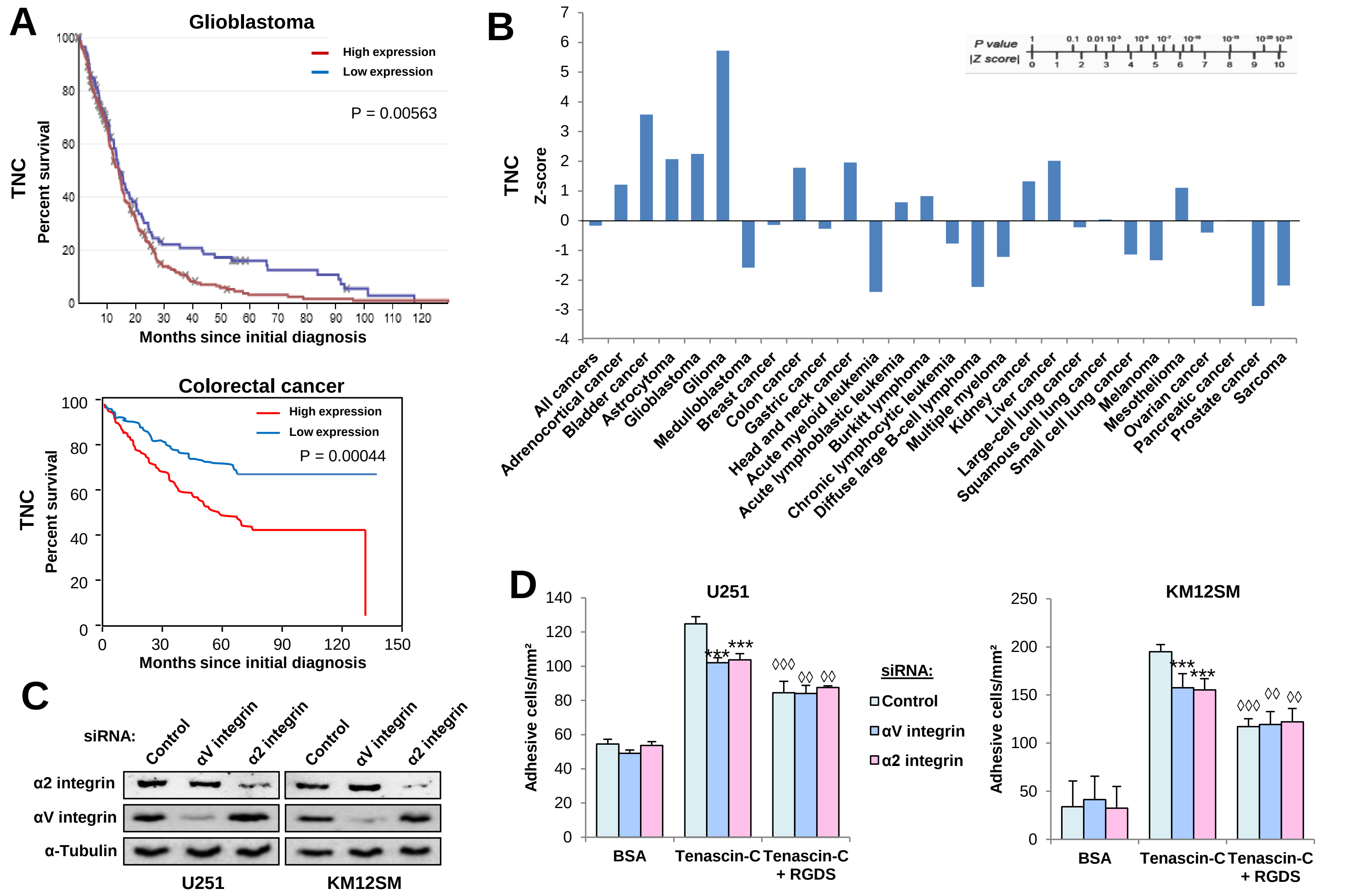

Figure S2. In silico analysis of cancer patient survival according to Tenascin-C expression. Integrins involved in tenascin-C adhesion. (A) In silico analysis by Kaplan-Meyer survival of glioblastoma and colorectal cancer patients showing a statistical correlation between high expression of TNC with poor overall survival. Inside each panel the p value of the log-rank analysis is shown. (B) Association between expression levels of TNC and poor overall survival according to the PRECOG web tool (https://www.precog.stanford.edu) by cancer type. (C) The indicated cell lines were transfected with siRNAs targeting α2 or αV integrins or control. After 48 h cells were lysed, and the extracts analyzed by Western blot to assess the silencing of integrin expression. (D) The same transfectants were subjected to adhesion assays to Tenascin-C in the presence or absence of RGDS peptides (0.5 mM). Silencing of the indicated integrin subunits (*) or the addition or RGDS peptide (◊) significantly inhibited cell adhesion to Tenascin-C (***, $p < 0.001$; ◊◊, $p < 0.01$; ◊◊◊, $p < 0.001$).

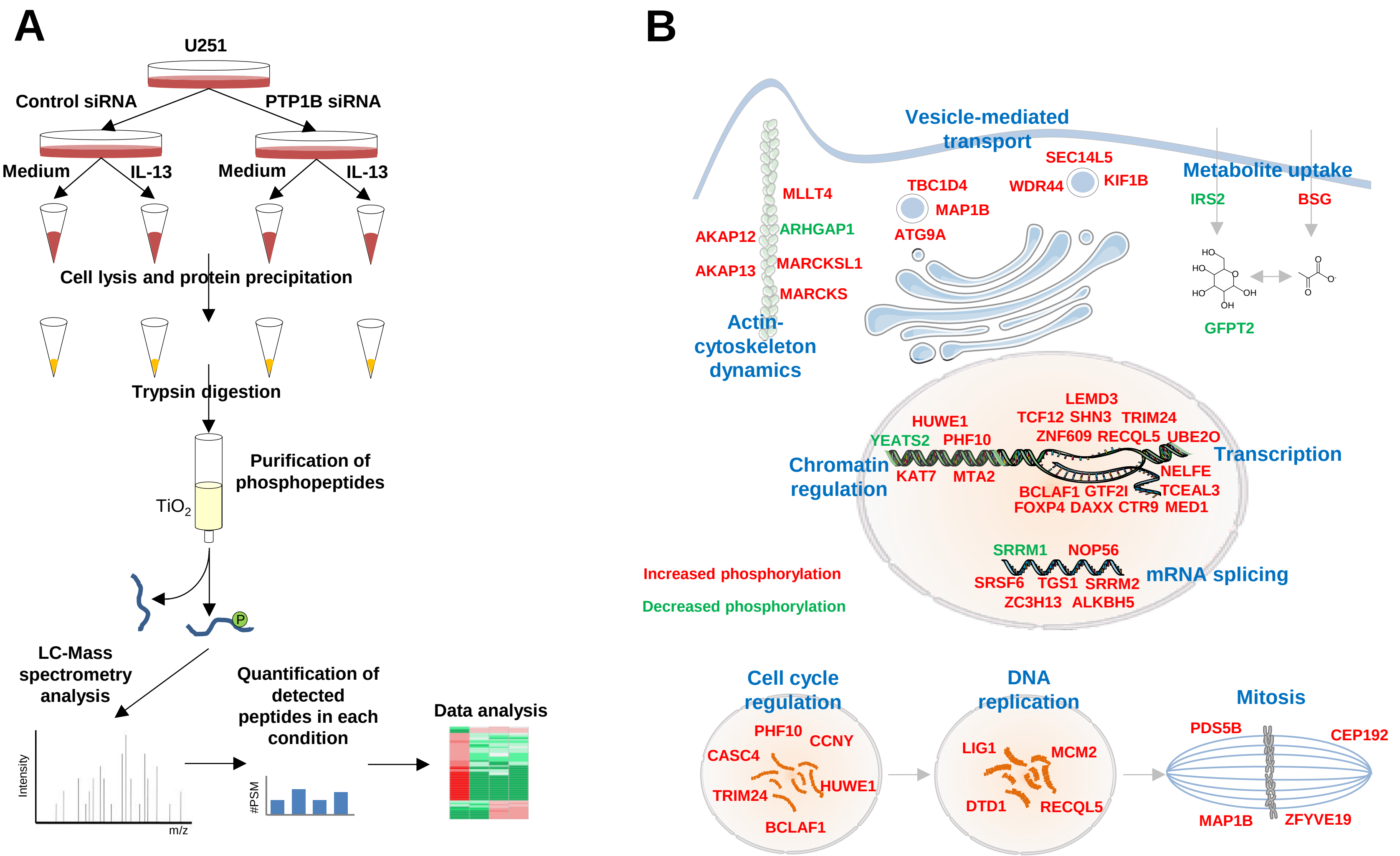

Figure S3. Phosphoproteomic analysis of PTP1B and IL-13 in cancer cells. (A) U251 cells were silenced for PTP1B expression, exposed to IL-13 and lysed. The extracts were digested with trypsin and the phosphopeptides were purified in columns of TiO_2 and analyzed by mass spectrometry. Quantification of each phosphorylated peptide in each condition reveals a pattern according to IL-13 and/or PTP1B involvement in such phosphorylation. (B) Representation of the proteins whose phosphorylation status was affected by IL-13 through PTP1B and the biological processes in which such proteins are involved according to Gene Ontology Biological Process and pertinent literature. Proteins whose phosphorylation status was enhanced by IL-13/PTP1B are written in red, whereas those proteins whose phosphorylation was reduced by IL-13/PTP1B are written in green.

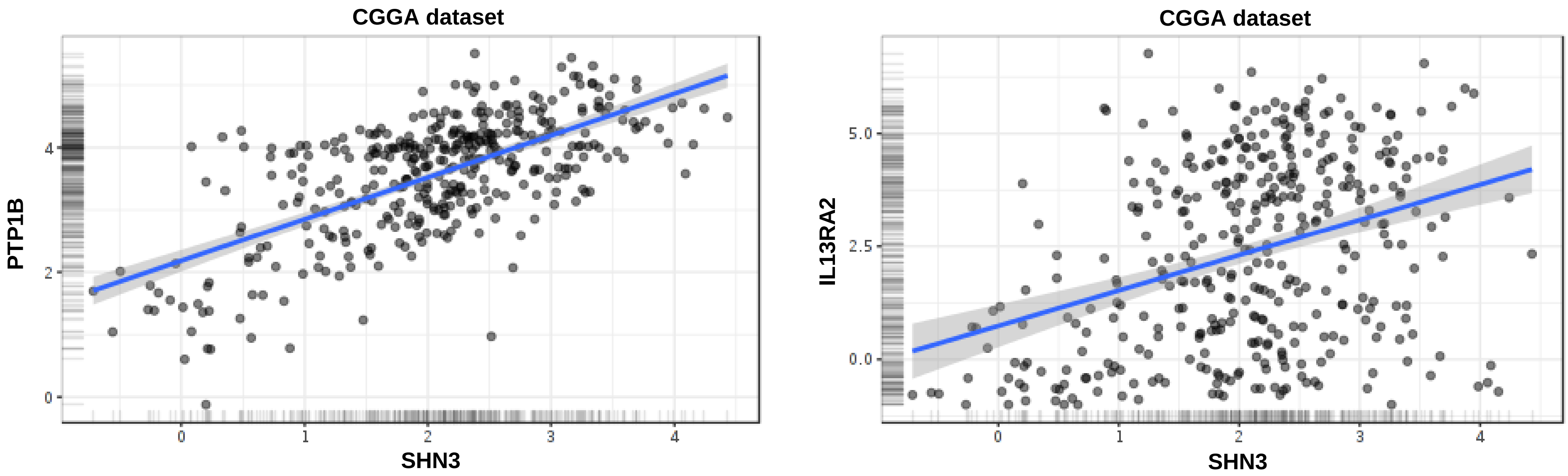

Pearson's correlation coefficient = 0.66
P = 0.0003

Pearson's correlation coefficient = 0.35
P = 0.002

Figure S4 SHN3 expression levels correlate with PTP1B and IL13Rα2 expressions. Correlation between the expression levels of SHN3 and PTP1B or IL13Rα2 according to CGGA dataset glioblastoma samples. Pearson's correlation coefficient and Pearson correlation test values are shown below.

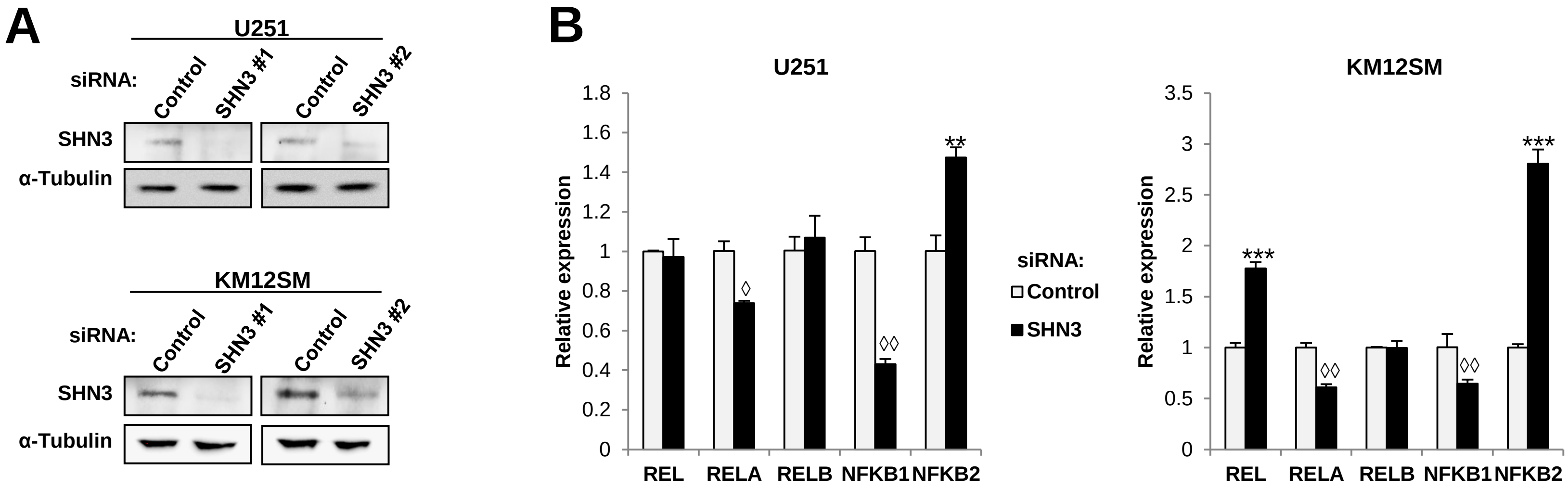

Figure S5. SHN3 regulates NF-κB expression. (A) U251 and KM12SM cells were transfected with SHN3-targeting or control siRNAs, and, after 48 h, lysed. The extracts were analyzed by Western blot to assess the downregulation of SHN3 expression. RhoGDI was used as loading control. (B) The mRNA from the same transfectants was isolated, retrotranscribed and subjected to qPCR assays to detect the expression of the indicated NF-κB subunit genes. The silencing of SHN3 causes a significant increase (**, $p < 0.01$; ***, $p < 0.001$) or decrease (◊, $p < 0.05$; ◊◊, $p < 0.01$) in the expression of the indicated genes.

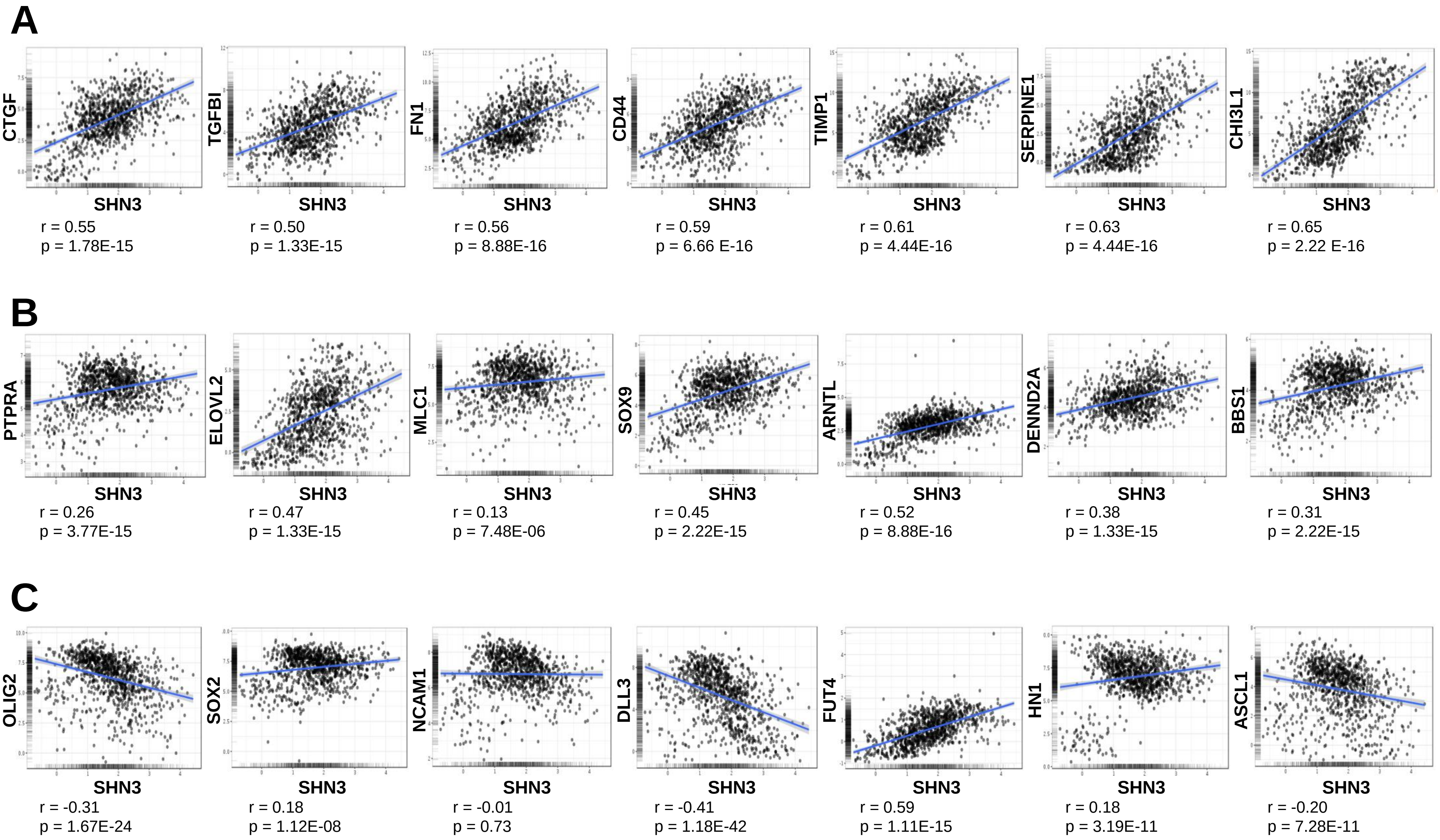

Figure S6 SHN3 expression correlates with mesenchymal and classical subtype signatures in glioblastoma. Correlation between the expression levels of SHN3 and the indicated mesenchymal (A), classical (B) or proneural (C) markers according to CGGA dataset glioblastoma samples. Pearson’s correlation coefficient and Pearson correlation test values are shown below each panel.

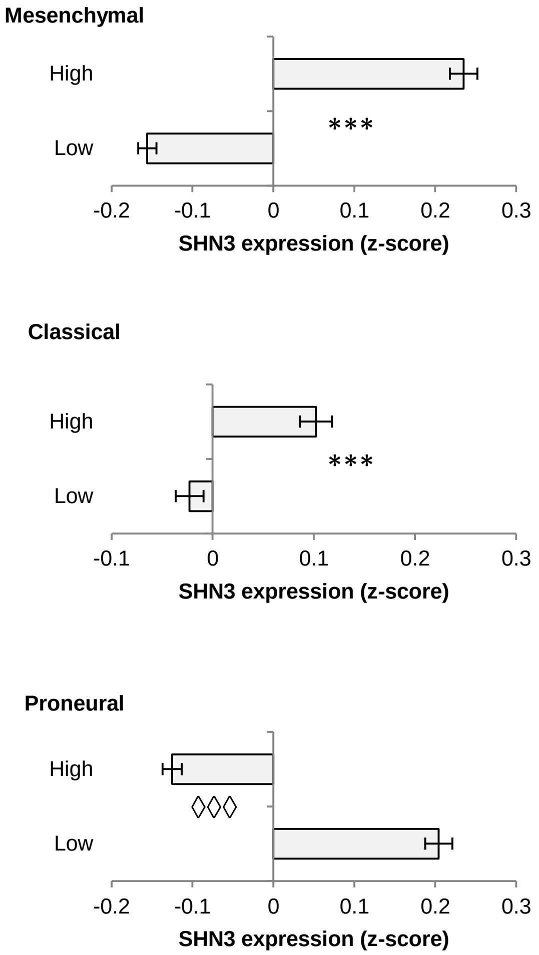

Figure S7 SHN3 expression correlates with mesenchymal and classical subtypes in glioblastoma. SHN3 expression levels (z-scores) were significantly (***, $p < 0.001$) higher in GBM cells with high values of mesenchymal or classical meta-modules, and significantly (◊◊◊, $p < 0.001$) lower in GBM cells with high values of proneural meta-module, according to SCP393 GBM scRNAseq dataset. Cell populations was divided by the median of the indicated meta-module. Error bars indicate the standard error.

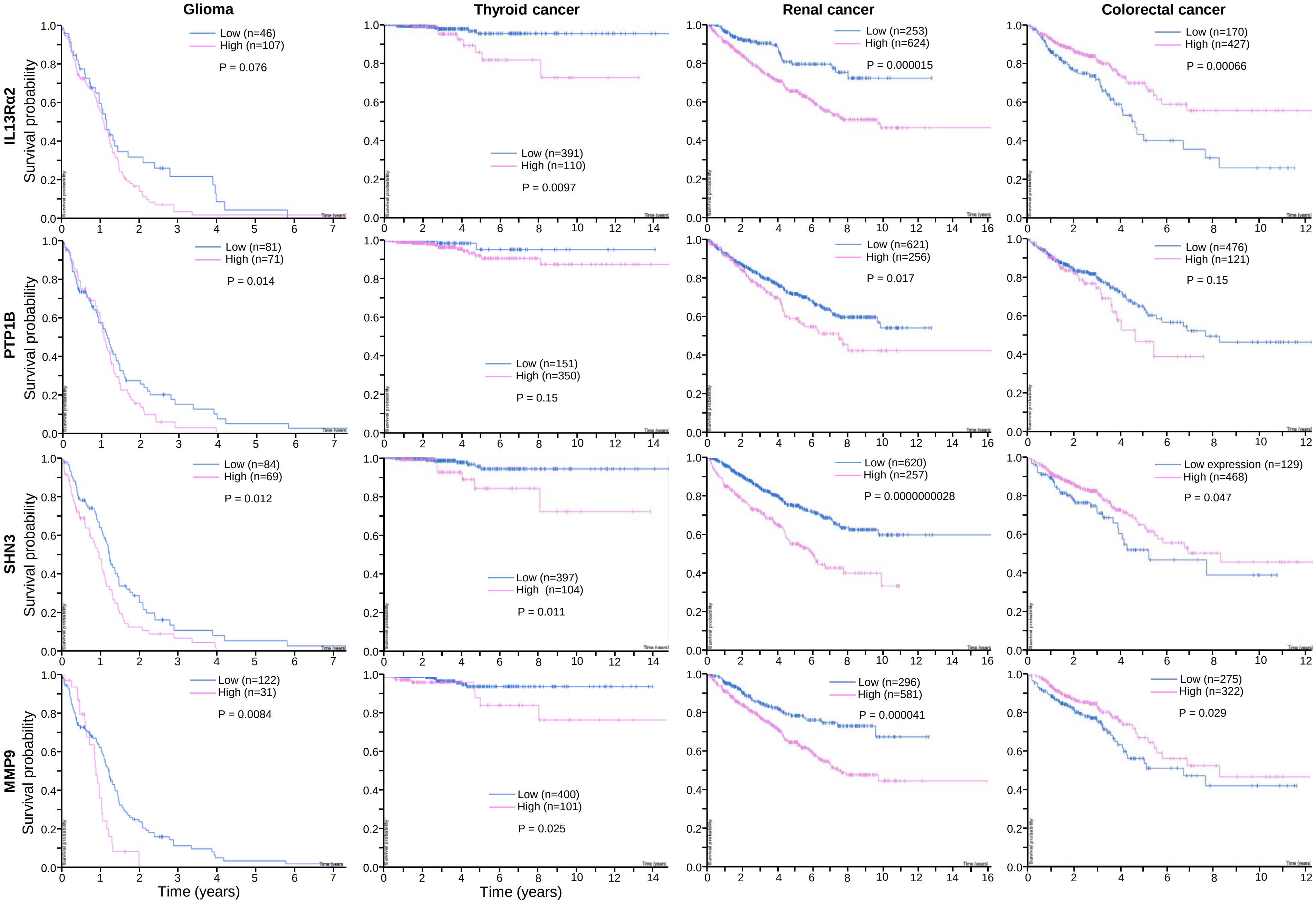

Figure S8. Association of IL13Rα2, SHN3 and PTP1B expression levels with prognosis in cancer patients. In silico analysis by Kaplan-Meyer survival of the indicated cancer patients according to the expression levels of IL13Rα2, SHN3 and PTP1B according to the Human Protein Atlas (https://www.proteinatlas.org). Inside each panel the p value of the log-rank analysis is shown together with the number of patients in each group (high and low expression samples).

Supplementary table S1. Proteins coimmunoprecipitated with PTP1B

Accession	Name	Description	Score	Coverage	Peptides	PSM
P18031	PTPN1	Tyrosine-protein phosphatase non-receptor type 1	205.67	60.92	23	68
Q04206	RELA	Transcription factor p65	93.38	38.36	17	34
Q68CZ2	TNS3	Tensin-3	42.02	9.20	8	11
Q9H4M9	EHD1	EH domain-containing protein 1	38.60	27.53	10	11
A1L0T0	ILVBL	Acetolactate synthase-like protein	25.15	5.22	2	5
O14579	COPE	Coatomer subunit epsilon	25.08	17.53	2	6
Q15653	NFKBIB	NF-kappa-B inhibitor beta	24.77	23.67	6	8
Q15075	EEA1	Early endosome antigen 1	23.89	6.17	6	6
Q14166	TTLL12	Tubulin--tyrosine ligase-like protein 12	21.42	14.13	6	7
Q9H4A6	GOLPH3	Golgi phosphoprotein 3	18.96	9.73	2	4
Q9Y4G6	TLN2	Talin-2	18.24	2.60	6	6
O00221	NFKBIE	NF-kappa-B inhibitor epsilon	18.06	13.00	4	5
P09960	LTA4H	Leukotriene A-4 hydrolase	17.15	11.41	5	5
Q9H9H4	VPS37B	Vacuolar protein sorting-associated protein 37B	16.44	17.19	3	4
Q96DA0	ZG16B	Zymogen granule protein 16 homolog B	16.33	25.96	4	5
Q86VI3	IQGAP3	Ras GTPase-activating-like protein IQGAP3	16.21	2.70	3	4
Q96G03	PGM2	Phosphoglucomutase-2	15.78	9.31	4	6
P13807	GYS1	Glycogen [starch] synthase, muscle	15.59	6.54	4	4
Q9NSC5	HOMER3	Homer protein homolog 3	15.47	18.15	5	5
Q9NZB2	FAM120A	Constitutive coactivator of PPAR-gamma-like protein 1	13.14	4.20	3	4
O14828	SCAMP3	Secretory carrier-associated membrane protein 3	12.75	9.03	2	3
Q9NPQ8	RIC8A	Synembryn-A	11.57	9.06	3	3
Q15257	PPP2R4	Serine/threonine-protein phosphatase 2A activator	11.32	13.27	3	4
P49589	CARS	Cysteine--tRNA ligase, cytoplasmic	11.19	5.65	3	3
Q9NUM3	SLC39A9	Zinc transporter ZIP9	11.01	6.47	1	3
Q2M389	WASH7	WASH complex subunit 7	10.53	3.15	3	3
P07384	CAPN1	Calpain-1 catalytic subunit	10.45	5.60	3	3
O43747	AP1G1	AP-1 complex subunit gamma-1	9.58	5.35	3	3
P00568	AK1	Adenylate kinase isoenzyme 1	9.33	19.07	3	3
Q9UPN3	MACF1	Microtubule-actin cross-linking factor 1	9.32	0.64	3	3
Q9NRW7	VPS45	Vacuolar protein sorting-associated protein 45	9.31	6.84	3	3
Q9UG63	ABCF2	ATP-binding cassette sub-family F member 2	8.76	3.53	2	3
Q9H2G2	SLK	STE20-like serine/threonine-protein kinase	8.72	3.07	3	3
Q9NRY5	FAM114A2	Protein FAM114A2	8.65	8.12	3	3
Q9UID3	VPS51	Vacuolar protein sorting-associated protein 51 homolog	8.58	5.32	2	2
P49023	PXN	Paxillin	8.33	5.57	2	2
P02751	FN1	Fibronectin	7.70	1.48	2	3
P60983	GMFB	Glia maturation factor beta	7.46	22.54	2	2
Q86Y56	DNAAF5	Dynein assembly factor 5, axonemal	7.19	14.47	2	2
Q13003	GRIK3	Glutamate receptor ionotropic, kainate 3	7.19	1.83	1	2
P08962	CD63	CD63 antigen	7.18	11.54	2	3
O60333	KIF1B	Kinesin-like protein KIF1B	7.02	1.91	2	3
Q99816	TSG101	Tumor susceptibility gene 101 protein	6.56	7.72	2	2
O15427	SLC16A3	Monocarboxylate transporter 4	5.82	5.59	2	2
Q96L33	RHOV	Rho-related GTP-binding protein RhoV	4.95	4.66	1	2
P24821	TNC	Tenascin C	4.95	1.41	2	2
Q6PHW0	IYD1	Iodotyrosine deiodinase 1	4.49	6.25	1	2
O14964	HGS	Hepatocyte growth factor-regulated tyrosine kinase substrate	4.34	3.04	2	2

Q5VW36	FOCAD	Focadhesin	3.55	0.78	1	2
P78504	JAG1	Protein jagged-1	0.00	1.89	1	2
Q9Y6K9	NEMO	NF-kappa-B essential modulator	3.28	3.58	1	1
Q5ZPR3	CD276	CD276 antigen	2.47	4.11	1	1
P23229	ITGA6	Integrin alpha-6	2.40	0.75	1	1
P19784	CSNK2A2	Casein kinase II subunit alpha'	2.35	4.00	1	1
Q14738	PPP2R5D	Serine/threonine-protein phosphatase 2A 56 kDa regulatory subunit delta	2.30	2.22	1	1
O95163	IKBKAP	Elongator complex protein 1	4.20	1.65	1	1

Supplementary table 2. PTP1B BioID interacting proteins

Accession	Name	Description	LFQ intensity (log_2)						Fold change (PTP1B vs. Control)	SP value
			Control #1	Control #2	Control #3	PTP1B #1	PTP1B #2	PTP1B #3		
P18031	PTPN1	Tyrosine-protein phosphatase non-receptor type 1	20.09	20.37	19.59	31.88	32.08	31.86	3108.87	1
P13667	PDIA4	Protein disulfide-isomerase A4	1.00	1.00	1.00	27.53	27.53	27.53	364.43	1
Q14254	FLOT2	Flotillin-2	18.20	19.00	19.77	28.30	28.20	28.26	337.09	1
Q96R06	SPAG5	Sperm-associated antigen 5	1.00	1.00	1.00	27.23	27.18	27.04	281	1
Q9Y4L1	HYOU1	Hypoxia up-regulated protein 1	1.00	1.00	1.00	27.05	26.85	27.09	252.67	1
O95613	PCNT	Pericentrin	20.35	19.78	20.51	28.51	28.30	28.43	249.3	1
P52732	KIF11	Kinesin-like protein KIF11	19.86	19.52	19.16	28.05	27.90	27.94	241.09	1
Q63HN8	RNF213	E3 ubiquitin-protein ligase RNF213	19.22	18.29	19.35	27.70	28.01	26.76	218.54	1
O15173	PGRMC2	Membrane-associated progesterone receptor component 2	1.00	1.00	1.00	26.60	26.93	26.80	217.66	1
Q99567	NUP88	Nuclear pore complex protein Nup88	1.00	1.00	1.00	26.61	26.44	26.76	193.1	1
P11441	UBL4A	Ubiquitin-like protein 4A	1.00	1.00	1.00	26.38	26.20	26.61	167.73	1
Q9UMX0	UBQLN1	Ubiquilin-1	1.00	1.00	1.00	26.39	26.44	26.29	164.35	1
P49069	CAMLG	Guided entry of tail-anchored proteins factor CAMLG	1.00	1.00	1.00	26.37	26.04	26.50	157.76	1
P47929	LGALS7	Galectin-7	1.00	1.00	1.00	26.15	26.03	26.25	140.12	1
P07237	P4HB	Protein disulfide-isomerase	19.87	19.59	19.46	27.22	27.21	27.18	136.72	1
Q9NZT1	CALML5	Calmodulin-like protein 5	1.00	1.00	1.00	26.02	25.98	26.28	135.72	1
P49257	LMAN1	Protein ERGIC-53	1.00	1.00	1.00	26.01	26.26	25.86	131.6	1
Q15751	HERC1	Probable E3 ubiquitin-protein ligase HERC1	19.47	20.49	20.47	27.73	27.68	26.73	129.82	1
Q9NYU2	UGGT1	UDP-glucose:glycoprotein glucosyltransferase 1	1.00	1.00	1.00	25.88	25.98	25.71	115.24	1
Q99460	PSMD1	26S proteasome non-ATPase regulatory subunit 1	1.00	1.00	1.00	25.65	25.29	25.46	88.66	1
Q8TBA6	GOLGA5	Golgin subfamily A member 5	1.00	1.00	1.00	25.44	25.30	25.53	85.41	1
Q8IXH7	NELFCD	Negative elongation factor C/D	1.00	1.00	1.00	25.24	25.52	25.39	83.33	1
Q99707	MTR	Methionine synthase	1.00	1.00	1.00	25.46	25.22	25.42	82.28	1
Q86TG7	PEG10	Retrotransposon-derived protein PEG10	1.00	1.00	1.00	25.24	25.40	25.24	78.18	1
O75955	FLOT1	Flotillin-1	1.00	1.00	1.00	25.26	25.11	25.42	76.65	1
P42566	EPS15	Epidermal growth factor receptor substrate 15	1.00	1.00	1.00	25.09	25.48	25.18	76.23	1
O60884	DNAJA2	DnaJ homolog subfamily A member 2	1.00	1.00	1.00	25.19	25.29	25.23	75.22	1
Q96SN8	CDK5RAP2	CDK5 regulatory subunit-associated protein 2	1.00	1.00	1.00	25.23	25.22	25.21	74.25	1
Q86XP1	DGKH	Diacylglycerol kinase eta	1.00	1.00	1.00	25.21	25.08	25.21	71.77	1
O43852	CALU	Calumenin	1.00	1.00	1.00	24.94	25.06	24.86	62.01	1

Q96CP6	GRAMD1A	Protein Aster-A	1.00	1.00	1.00	24.91	25.12	24.77	61.49	1
Q9H0L4	CSTF2T	Cleavage stimulation factor subunit 2 tau variant	1.00	1.00	1.00	24.81	24.75	24.84	55.84	1
Q9NRR5	UBQLN4	Ubiquilin-4	1.00	1.00	1.00	24.74	24.83	24.61	53.14	1
Q6P179	ERAP2	Endoplasmic reticulum aminopeptidase 2	1.00	1.00	1.00	24.57	25.03	24.42	52	1
Q8N766	EMC1	ER membrane protein complex subunit 1	1.00	1.00	1.00	24.64	24.46	24.76	49.55	1
Q15365	PCBP1	Poly(rC)-binding protein 1	1.00	1.00	1.00	24.16	24.68	24.68	46.04	1
P51648	ALDH3A2	Aldehyde dehydrogenase family 3 member A2 1	1.00	1.00	1.00	24.53	24.44	24.52	45.46	1
P27797	CALR	Calreticulin	1.00	1.00	1.00	24.71	24.39	24.25	44.6	1
Q15293	RCN1	Reticulocalbin-1	1.00	1.00	1.00	24.37	24.37	24.42	42.2	1
P14314	PRKCSH	Glucosidase 2 subunit beta	1.00	1.00	1.00	24.40	24.29	24.30	40.56	1
P80303	NUCB2	Nucleobindin-2	1.00	1.00	1.00	24.29	24.31	24.19	38.82	1
Q15398	DLGAP5	Disks large-associated protein 5	1.00	1.00	1.00	24.08	24.14	24.33	36.73	1
P30533	LRPAP1	Alpha-2-macroglobulin receptor-associated protein	1.00	1.00	1.00	24.00	23.82	24.08	31.86	1
Q8TD26	CHD6	Chromodomain-helicase-DNA-binding protein 6	1.00	1.00	1.00	23.72	23.84	23.76	27.97	1
Q13162	PRDX4	Peroxiredoxin-4	1.00	1.00	1.00	24.05	24.26	24.28	37.05	0.99
Q96A33	CCDC47	PAT complex subunit CCDC47	1.00	1.00	1.00	23.91	23.99	23.98	31.58	0.99
Q14697	GANAB	Neutral alpha-glucosidase AB	19.17	21.59	18.67	25.12	25.71	25.12	27.81	0.99
Q07065	CKAP4	Cytoskeleton-associated protein 4	20.98	19.81	20.16	25.35	25.44	25.07	27.02	0.99
Q86UW6	N4BP2	NEDD4-binding protein 2	1.00	1.00	1.00	23.65	23.68	23.72	26.25	0.99
Q92575	UBXN4	UBX domain-containing protein 4	1.00	1.00	1.00	23.59	23.63	23.51	24.49	0.99
Q969V3	NCLN	Nicalin	1.00	1.00	1.00	23.64	23.34	23.50	23.25	0.99
P30040	ERP29	Endoplasmic reticulum resident protein 29	1.00	1.00	1.00	23.28	23.66	23.52	23.1	0.99
Q8IXB1	DNAJC10	DnaJ homolog subfamily C member 10	1.00	1.00	1.00	23.50	23.29	23.47	22.15	0.99
Q7Z4H7	HAUS6	HAUS augmin-like complex subunit 6	23.87	23.79	23.77	27.50	27.83	27.52	18.34	0.99
Q15836	VAMP3	Vesicle-associated membrane protein 3	1.00	1.00	1.00	24.13	24.41	23.70	34.95	0.98
Q3B7T1	EDRF1	Erythroid differentiation-related factor 1	1.00	1.00	1.00	23.59	23.32	23.58	23.32	0.98
Q9NX40	OCIAD1	OCIA domain-containing protein 1	1.00	1.00	1.00	23.41	23.26	23.49	21.59	0.98
Q9H3N1	TMX1	Thioredoxin-related transmembrane protein 1	1.00	1.00	1.00	23.13	23.42	23.53	21.26	0.98
Q9NQX4	MYO5C	Unconventional myosin-Vc	1.00	1.00	1.00	23.18	23.00	23.22	18.31	0.98
O94964	SOGA1	Protein SOGA1	1.00	1.00	1.00	23.01	23.22	23.16	18.23	0.98
Q9NXE4	SMPD4	Sphingomyelin phosphodiesterase 4	1.00	1.00	1.00	23.30	23.03	22.98	18.05	0.98
Q9UHG3	PCYOX1	Prenylcysteine oxidase 1	1.00	1.00	1.00	22.87	23.09	23.15	17.18	0.98
Q9UMX5	NENF	Neudesin	1.00	1.00	1.00	22.92	22.95	22.78	15.55	0.98

Q13561	DCTN2	Dynactin subunit 2	1.00	1.00	1.00	22.91	22.88	22.86	15.53	0.98
O95721	SNAP29	Synaptosomal-associated protein 29	1.00	1.00	1.00	22.89	22.88	22.85	15.4	0.98
P46939	UTRN	Utrophin	20.56	19.77	20.70	25.45	24.92	24.45	22.2	0.97
P50748	KNTC1	Kinetochore-associated protein 1	1.00	1.00	1.00	23.22	23.38	23.11	19.6	0.97
Q9UHD2	TBK1	Serine/threonine-protein kinase TBK1	1.00	1.00	1.00	23.20	23.08	23.04	17.97	0.97
Q8IWW6	ARHGAP12	Rho GTPase-activating protein 12	1.00	1.00	1.00	23.09	23.17	22.97	17.63	0.97
Q9Y5K5	UCHL5	Ubiquitin carboxyl-terminal hydrolase isozyme L5	1.00	1.00	1.00	23.19	22.98	22.97	17.34	0.97
Q5JRA6	MIA3	Transport and Golgi organization protein 1 homolog	1.00	1.00	1.00	23.14	22.81	23.12	17.11	0.97
P50454	SERPINH1	Serpin H1	20.95	19.42	20.66	26.01	25.96	26.13	43.2	0.96
Q15084	PDIA6	Protein disulfide-isomerase A6	22.44	22.52	22.74	26.98	27.24	27.19	29.12	0.96
P14625	HSP90B1	Endoplasmin	25.25	25.32	25.27	28.70	29.38	28.80	17.59	0.96
Q5T5Y3	CAMSAP1	Calmodulin-regulated spectrin-associated protein 1	1.00	1.00	1.00	23.11	23.01	22.84	16.65	0.96
Q15208	STK38	Serine/threonine-protein kinase 38	1.00	1.00	1.00	22.85	23.20	22.66	15.91	0.96
O60502	MGEA5	Protein O-GlcNAcase	1.00	1.00	1.00	22.89	22.75	23.10	15.89	0.96
Q96A49	SYAP1	Synapse-associated protein 1	1.00	1.00	1.00	23.04	22.86	22.75	15.57	0.96
Q92667	AKAP1	A-kinase anchor protein 1, mitochondrial	1.00	1.00	1.00	22.94	22.79	22.92	15.53	0.96
Q9UNF1	MAGED2	Melanoma-associated antigen D2	1.00	1.00	1.00	22.84	23.08	22.63	15.33	0.96
P40855	PEX19	Peroxisomal biogenesis factor 19	1.00	1.00	1.00	22.78	22.83	22.97	15.28	0.96
O75665	OFD1	Oral-facial-digital syndrome 1 protein	1.00	1.00	1.00	22.70	22.64	22.70	13.63	0.96
Q12979	ABR	Active breakpoint cluster region-related protein	1.00	1.00	1.00	23.16	22.68	22.80	15.7	0.95
P30876	POLR2B	DNA-directed RNA polymerase II subunit RPB2	1.00	1.00	1.00	22.73	22.93	23.00	15.54	0.95
Q99614	TTC1	Tetratricopeptide repeat protein 1	1.00	1.00	1.00	22.66	22.72	23.06	14.9	0.95
Q92540	SMG7	Protein SMG7	1.00	1.00	1.00	22.88	22.75	22.74	14.62	0.95
A5YKK6	CNOT1	CCR4-NOT transcription complex subunit 1	1.00	1.00	1.00	22.76	22.61	22.77	13.93	0.95
Q8N1I0	DOCK4	Dedicator of cytokinesis protein 4	1.00	1.00	1.00	22.67	22.81	22.50	13.49	0.95
P27824	CANX	Calnexin	21.31	19.51	19.44	26.74	26.87	26.64	74.75	0.94
Q8N511	TMEM199	Transmembrane protein 199	1.00	1.00	1.00	22.56	23.14	23.24	16.76	0.94
Q96A65	EXOC4	Exocyst complex component 4	22.89	23.25	23.05	26.56	26.65	26.66	14.94	0.94
P42356	PI4KA	Phosphatidylinositol 4-kinase alpha	1.00	1.00	1.00	22.88	22.68	22.72	14.36	0.94
P16333	NCK1	Cytoplasmic protein NCK1	1.00	1.00	1.00	22.70	22.91	22.53	13.97	0.94
Q96B96	TMEM159	Lipid droplet assembly factor 1	1.00	1.00	1.00	22.52	22.50	22.76	12.91	0.94
Q14596	NBR1	Next to BRCA1 gene 1 protein	1.00	1.00	1.00	23.10	22.67	22.69	15.09	0.93
Q5VZ89	DENND4C	DENN domain-containing protein 4C	1.00	1.00	1.00	22.96	22.95	22.46	14.81	0.93

Q9H3U1	UNC45A	Protein unc-45 homolog A	1.00	1.00	1.00	22.47	22.55	22.61	12.48	0.93
Q9NVI1	FANCI	Fanconi anemia group I protein	1.00	1.00	1.00	22.43	22.44	22.57	11.99	0.93
Q14974	KPNB1	Importin subunit beta-1	20.34	19.29	21.06	27.27	26.75	27.17	92.3	0.92
P30101	PDIA3	Protein disulfide-isomerase A3	22.74	22.25	21.05	27.64	27.50	27.71	52.15	0.92
Q14696	MESDC2	LRP chaperone MESD	1.00	1.00	1.00	22.67	22.40	23.05	14.05	0.92
Q96BY7	ATG2B	Autophagy-related protein 2 homolog B	23.39	22.48	22.90	27.10	27.27	26.97	22.42	0.91
O95373	IPO7	Importin-7 1	1.00	1.00	1.00	22.61	22.62	24.23	21.07	0.91
Q9Y4R8	TELO2	Telomere length regulation protein TEL2 homolog	1.00	1.00	1.00	22.66	22.43	22.55	12.52	0.91
Q13217	DNAJC3	DnaJ homolog subfamily C member 3	1.00	1.00	1.00	22.69	22.21	22.43	11.84	0.91
Q8IWZ3	ANKHD1	Ankyrin repeat and KH domain-containing protein 1	1.00	1.00	1.00	22.06	22.50	22.70	11.67	0.91
O75976	CPD	Carboxypeptidase D	1.00	1.00	1.00	22.38	22.43	22.41	11.43	0.91
P47755	CAPZA2	F-actin-capping protein subunit alpha-2	1.00	1.00	1.00	22.34	22.41	22.38	11.2	0.91
Q8TCG1	KIAA1524	Protein CIP2A	1.00	1.00	1.00	22.40	22.11	22.39	10.75	0.91
O60749	SNX2	Sorting nexin-2	1.00	1.00	1.00	22.48	22.23	22.16	10.69	0.91
P04843	RPN1	Ribophorin 1	20.03	18.69	21.93	24.99	24.95	26.39	27.25	0.9
O95372	LYPLA2	Acyl-protein thioesterase 2	1.00	1.00	1.00	23.01	22.28	22.50	13.25	0.9
Q6PML9	SLC30A9	Zinc transporter 9	1.00	1.00	1.00	22.79	22.28	22.48	12.42	0.9
Q5JTV8	TOR1AIP1	Torsin-1A-interacting protein 1	1.00	1.00	1.00	22.59	22.52	22.30	11.95	0.9
O15126	SCAMP1	Secretory carrier-associated membrane protein 1	1.00	1.00	1.00	22.40	22.19	22.54	11.27	0.9
Q9Y2B0	CNPY2	Protein canopy homolog 2	1.00	1.00	1.00	22.60	21.96	22.38	10.99	0.9
Q15393	SF3B3	Splicing factor 3B subunit 3	1.00	1.00	1.00	21.97	22.47	22.39	10.6	0.9
O00232	PSMD12	26S proteasome non-ATPase regulatory subunit 12	1.00	1.00	1.00	22.10	22.20	22.08	9.58	0.9
Q8TCU4	ALMS1	Alstrom syndrome protein 1	19.96	19.14	20.58	26.27	26.26	24.69	48.87	0.89
Q8IWJ2	GCC2	GRIP and coiled-coil domain-containing protein 2	1.00	1.00	1.00	23.08	22.29	23.21	15.82	0.89
P78344	EIF4G2	Eukaryotic translation initiation factor 4 gamma 2	1.00	1.00	1.00	22.36	22.64	21.99	11.08	0.89
Q9C0B7	TANGO6	Transport and Golgi organization protein 6 homolog	1.00	1.00	1.00	22.39	21.89	22.25	10.01	0.89
O95905	ECD	Protein ecdysoneless homolog	1.00	1.00	1.00	22.09	22.23	22.25	9.98	0.89
Q8NDI1	EHBP1	EH domain-binding protein 1	1.00	1.00	1.00	22.24	22.01	22.07	9.5	0.89
Q709C8	VPS13C	Vacuolar protein sorting-associated protein 13C	1.00	1.00	1.00	22.24	21.94	21.97	9.21	0.89
P06493	CDK1	Cyclin-dependent kinase 1	1.00	1.00	1.00	22.39	22.06	22.57	11.07	0.88
Q14257	RCN2	Reticulocalbin-2	1.00	1.00	1.00	22.23	22.58	21.97	10.57	0.88
Q92538	GBF1	Golgi brefeldin A resistant guanine nucleotide exchange factor 1	1.00	1.00	1.00	21.92	22.37	22.49	10.5	0.88
Q08378	GOLGA3	Golgin subfamily A member 3	1.00	1.00	1.00	22.20	22.07	22.12	9.62	0.88

Q9Y484	WDR45	WD repeat domain phosphoinositide-interacting protein 4	1.00	1.00	1.00	22.10	22.14	22.06	9.43	0.88
A8MXV4	NUDT19	Nucleoside diphosphate-linked moiety X motif 19	1.00	1.00	1.00	22.08	22.14	22.03	9.35	0.88
Q9H0U4	RAB1B	Ras-related protein Rab-1B	1.00	1.00	1.00	22.01	22.26	22.21	9.79	0.87
P78381	SLC35A2	UDP-galactose translocator	1.00	1.00	1.00	22.18	22.11	21.99	9.41	0.87
Q12800	TFCP2	Alpha-globin transcription factor CP2	1.00	1.00	1.00	21.88	22.17	22.18	9.31	0.87
Q9H8Y8	GORASP2	Golgi reassembly-stacking protein 2	1.00	1.00	1.00	23.29	22.25	23.49	17.95	0.86
P07195	LDHB	L-lactate dehydrogenase B chain	1.00	1.00	1.00	23.18	22.23	22.81	14.72	0.86
Q9UHD9	UBQLN2	Ubiquilin-2	1.00	1.00	1.00	22.19	22.28	21.93	9.69	0.86
P51572	BCAP31	B-cell receptor-associated protein 31	1.00	1.00	1.00	21.91	22.01	22.43	9.58	0.86
O14976	GAK	Cyclin-G-associated kinase	1.00	1.00	1.00	22.08	22.29	21.79	9.27	0.86
Q9BRZ2	TRIM56	E3 ubiquitin-protein ligase TRIM56	1.00	1.00	1.00	21.75	21.96	22.24	8.83	0.85
Q6P2Q9	PRPF8	Pre-mRNA-processing-splicing factor 8	22.09	21.13	21.78	25.72	25.75	25.47	17.47	0.84
Q96C34	RUNDC1	RUN domain-containing protein 1	1.00	1.00	1.00	22.07	22.52	21.83	9.83	0.84
Q86Y07	VRK2	Serine/threonine-protein kinase VRK2	1.00	1.00	1.00	21.84	22.11	22.13	9.04	0.84
Q9UHI6	DDX20	Probable ATP-dependent RNA helicase DDX20	1.00	1.00	1.00	22.12	21.71	22.06	8.74	0.83
Q9Y3P9	RABGAP1	Rab GTPase-activating protein 1	1.00	1.00	1.00	21.91	21.94	21.93	8.47	0.83
O43765	SGTA	Small glutamine-rich tetratricopeptide repeat-containing alpha	1.00	1.00	1.00	22.02	22.29	21.84	9.23	0.82
Q7Z434	MAVS	Mitochondrial antiviral-signaling protein	1.00	1.00	1.00	21.83	22.19	21.81	8.61	0.82
Q8N2F6	ARMC10	Armadillo repeat-containing protein 10	1.00	1.00	1.00	21.90	21.98	21.87	8.43	0.82
Q96RG2	PASK	PAS domain-containing serine/threonine-protein kinase	1.00	1.00	1.00	21.80	21.83	22.02	8.26	0.82
O60826	CCDC22	Coiled-coil domain-containing protein 22	1.00	1.00	1.00	21.72	21.82	21.95	7.99	0.82
Q8WVQ1	CANT1	Soluble calcium-activated nucleotidase 1	1.00	1.00	1.00	21.88	22.55	21.87	9.64	0.81
P98174	FGD1	FYVE, RhoGEF and PH domain-containing protein 1	1.00	1.00	1.00	21.74	21.99	21.86	8.17	0.81
Q2M2I8	AAK1	AP2-associated protein kinase 1	1.00	1.00	1.00	21.74	21.88	21.82	7.91	0.81
P28288	ABCD3	ATP-binding cassette sub-family D member 3	20.25	20.76	19.36	26.69	26.64	26.83	77.67	0.8
P57678	GEMIN4	Gem-associated protein 4	1.00	1.00	1.00	21.69	21.80	22.13	8.25	0.8
O14737	PDCD5	Programmed cell death protein 5	1.00	1.00	1.00	22.02	21.59	21.76	7.87	0.8
Q9NRY5	FAM114A2	Protein FAM114A2	1.00	1.00	1.00	21.91	21.54	21.76	7.61	0.8
Q13724	MOGS	Mannosyl-oligosaccharide glucosidase	1.00	1.00	1.00	21.71	21.74	21.70	7.47	0.8
Q99623	PHB2	Prohibitin-2	1.00	1.00	1.00	21.71	22.03	21.94	8.31	0.79
Q9Y295	DRG1	Developmentally-regulated GTP-binding protein 1	1.00	1.00	1.00	22.12	21.48	21.99	8.31	0.78
Q9NP61	ARFGAP3	ADP-ribosylation factor GTPase-activating protein 3	1.00	1.00	1.00	21.74	21.77	21.63	7.45	0.78
P55145	MANF	Mesencephalic astrocyte-derived neurotrophic factor	1.00	1.00	1.00	21.74	21.49	21.99	7.64	0.77

Q9BWS9	CHID1	Chitinase domain-containing protein 1	1.00	1.00	1.00	21.67	21.82	21.64	7.44	0.77
Q9H6S0	YTHDC2	3-5 RNA helicase YTHDC2	1.00	1.00	1.00	21.72	21.42	21.89	7.36	0.77
Q9H488	POFUT1	GDP-fucose protein O-fucosyltransferase 1	1.00	1.00	1.00	21.84	21.37	21.90	7.5	0.75
Q13283	G3BP1	Ras GTPase-activating protein-binding protein 1	1.00	1.00	1.00	21.62	22.57	21.53	8.78	0.74
P57740	NUP107	Nuclear pore complex protein Nup107	1.00	1.00	1.00	21.70	22.22	21.39	7.89	0.74
Q8IZP2	ST13P4	Putative protein FAM10A4	1.00	1.00	1.00	21.45	21.88	22.01	7.81	0.74
Q15417	CNN3	Calponin-3	1.00	1.00	1.00	21.67	21.45	21.80	7.15	0.74
P35998	PSMC2	26S proteasome regulatory subunit 7	21.19	18.59	21.13	24.33	24.26	24.34	12.05	0.73
Q8TAT6	NPLOC4	Nuclear protein localization protein 4 homolog	1.00	1.00	1.00	21.56	21.54	21.66	6.9	0.73
Q8TEM1	NUP210	Nuclear pore membrane glycoprotein 210	19.18	21.67	20.65	25.05	25.27	24.03	16.63	0.72
O15372	EIF3H	Eukaryotic translation initiation factor 3 subunit H	1.00	1.00	1.00	22.77	21.70	22.96	12.74	0.72
P42167	TMPO	Lamina-associated polypeptide 2, isoforms beta/gamma	26.45	26.31	26.17	28.83	28.83	29.05	8.18	0.72
P62280	RPS11	40S ribosomal protein S11	1.00	1.00	1.00	21.51	21.44	21.84	6.98	0.72
Q9UBS4	DNAJB11	DnaJ homolog subfamily B member 11	1.00	1.00	1.00	21.69	21.93	21.36	7.3	0.71
P78527	PRKDC	DNA-dependent protein kinase catalytic subunit	25.17	25.21	25.20	28.01	28.17	27.94	9.69	0.7
O14964	HGS	Hepatocyte growth factor-regulated tyrosine kinase substrate	1.00	1.00	1.00	21.63	21.56	21.51	6.84	0.7
Q96CW5	TUBGCP3	Gamma-tubulin complex component 3	1.00	1.00	1.00	21.57	21.56	21.48	6.71	0.7
Q96AY4	TTC28	Tetratricopeptide repeat protein 28	20.92	20.36	20.86	24.46	24.74	23.11	11.43	0.68
P48444	ARCN1	Coatomer subunit delta	1.00	1.00	1.00	21.98	21.30	21.93	7.71	0.68
Q4G148	GXYLT1	Glucoside xylosyltransferase 1	1.00	1.00	1.00	21.76	21.36	21.72	7.06	0.68
Q8TD19	NEK9	Serine/threonine-protein kinase Nek9	1.00	1.00	1.00	21.74	21.61	21.37	6.92	0.68
Q9NU22	MDN1	Midasin	1.00	1.00	1.00	23.42	23.88	21.92	20.21	0.67
P61011	SRP54	Signal recognition particle 54 kDa protein	1.00	1.00	1.00	21.59	21.29	21.70	6.72	0.67
P17980	PSMC3	26S proteasome regulatory subunit 6A	1.00	1.00	1.00	21.62	21.50	21.33	6.54	0.67
P26599	PTBP1	Polypyrimidine tract-binding protein 1	1.00	1.00	1.00	22.15	21.24	22.02	8.14	0.65
Q7KZF4	SND1	Staphylococcal nuclease domain-containing protein 1	1.00	1.00	1.00	21.55	21.90	21.25	6.93	0.65
O15084	ANKRD28	Phosphatase 6 regulatory ankyrin repeat subunit A	1.00	1.00	1.00	21.51	21.27	21.33	6.11	0.64
P23284	PPIB	Peptidyl-prolyl cis-trans isomerase B	22.61	19.93	20.10	24.82	25.27	26.17	18.69	0.63
O15498	YKT6	Synaptobrevin homolog YKT6	19.71	21.61	21.59	23.53	25.28	24.96	12.49	0.63
Q658Y4	FAM91A1	Protein FAM91A1	1.00	1.00	1.00	22.05	21.18	21.48	7.08	0.63
O75381	PEX14	Peroxisomal membrane protein PEX14	1.00	1.00	1.00	21.34	21.34	21.54	6.22	0.63
P50570	DNM2	Dynamin-2	1.00	1.00	1.00	21.57	21.18	21.31	6.08	0.62
Q6Y7W6	GIGYF2	GRB10-interacting GYF protein 2	25.26	25.30	25.21	28.05	28.22	27.95	9.53	0.61

P04844	RPN2	Ribophorin 2	20.96	20.92	21.04	24.29	23.68	23.40	7.69	0.61
Q15058	KIF14	Kinesin-like protein KIF14	1.00	1.00	1.00	21.63	21.18	21.43	6.29	0.61
Q6NZ36	FAAP20	Fanconi anemia core complex-associated protein 20	1.00	1.00	1.00	21.45	21.37	21.35	6.16	0.61
Q96JQ2	CLMN	Calmin	1.00	1.00	1.00	21.42	21.27	21.25	5.9	0.61
Q14789	GOLGB1	Golgin subfamily B member 1	21.56	19.45	20.47	25.01	23.96	24.20	13.21	0.6
Q9ULE6	PALD1	Paladin	1.00	1.00	1.00	21.61	21.10	21.34	6.08	0.6
Q14145	KEAP1	Kelch-like ECH-associated protein 1	21.44	21.29	21.74	24.31	24.31	24.18	7.67	0.59
O60566	BUB1B	Mitotic checkpoint serine/threonine-protein kinase BUB1 beta	20.99	21.15	21.05	22.83	23.67	24.28	6.58	0.58
Q9NRW1	RAB6B	Ras-related protein Rab-6B	1.00	1.00	1.00	21.11	21.64	21.28	6.03	0.58
Q96JH7	VCPIP1	Deubiquitinating protein VCPIP1	22.88	22.36	23.05	25.79	25.74	25.57	9.46	0.57
P46108	CRK	Adapter molecule crk	24.19	23.99	24.09	26.66	26.72	26.39	7.53	0.57
P43490	NAMPT	Nicotinamide phosphoribosyltransferase	1.00	1.00	1.00	21.45	22.21	20.99	7.12	0.55
Q12797	ASPH	Aspartyl/asparaginyl beta-hydroxylase	1.00	1.00	1.00	21.25	21.11	21.36	5.66	0.55
P43686	PSMC4	26S proteasome regulatory subunit 6B	1.00	1.00	1.00	21.57	21.11	21.15	5.83	0.54
Q641Q2	WASHC2A	WASH complex subunit 2A	21.25	21.65	21.73	23.96	23.94	24.21	6.37	0.53
P11021	HSPA5	Endoplasmic reticulum chaperone BiP	26.43	26.52	26.46	28.53	28.59	28.50	5.71	0.53
O75153	CLUH	Clustered mitochondria protein homolog	1.00	1.00	1.00	21.08	21.32	21.28	5.6	0.53
Q9P2J5	LARS	Leucine--tRNA ligase, cytoplasmic	19.41	22.22	20.32	24.57	25.08	24.65	13.26	0.52
Q99996	AKAP9	A-kinase anchor protein 9	20.29	20.55	19.18	23.89	22.92	22.92	8.18	0.52
Q9UPN7	PPP6R1	Trinucleotide repeat-containing gene 6B protein	1.00	1.00	1.00	21.41	21.04	21.17	5.58	0.52
Q9UBC2	EPS15L1	Epidermal growth factor receptor substrate 15-like 1	22.38	22.93	22.27	25.02	25.31	24.86	7.11	0.51
Q7Z6Z7	HUWE1	E3 ubiquitin-protein ligase HUWE1	22.07	22.48	21.86	24.90	24.47	24.59	6.88	0.51
Q68DQ2	CRYBG3	Very large A-kinase anchor protein	19.78	20.21	21.88	25.08	24.02	24.03	12.03	0.5

Supplementary Table S3: Proteins regulated by IL-13 through PTP1B

Protein accession	Protein name	Peptide sequence	Phospho-rylated residues	(A) siRNA control + IL-13 # PSMs	(B) siRNA control + Medium # PSMs	(C) siRNA PTP1B + IL-13 # PSMs	(D) siRNA PTP1B + Medium # PSMs	ratio A/B	ratio A/C	ratio A/D	ratio B/C	ratio B/D	ratio C/D
Q8IYB3	SRRM1	RRsPsPAPPPR	S3; S5	3	5	7	5	0.60	0.43	0.60	0.71	1.00	1.40
Q07960	ARHGAP1	SDDSKSSsPELVTHLK	S8	2	4	4	4	0.50	0.50	0.50	1.00	1.00	1.00
O94808	GFPT2	RLDsSAcLHAVGDK	S4	0	2	2	2	0.00	0.00	0.00	1.00	1.00	1.00
Q9Y4H2	IRS2	KSSEGGVGVGPGGGDEPPTsPR	S20	0	2	2	2	0.00	0.00	0.00	1.00	1.00	1.00
Q9ULM3	YEATS2	ASsPIKQSHEPVPDTSVEK	S3	0	2	2	2	0.00	0.00	0.00	1.00	1.00	1.00
Q8TEA8	DTD1	SASsGAEGDVSSEREP	S4	6	3	3	4	2.00	2.00	1.50	1.00	0.75	0.75
O60343	TBC1D4	tSStcSNESLSVGGTSVTPR	T1; T4	5	3	3	3	1.67	1.67	1.67	1.00	1.00	1.00
Q99081	TCF12	TSsTNEDEDLNPEQK	S3	8	3	2	3	2.67	4.00	2.67	1.50	1.00	0.67
Q8ND76	CCNY	SAsADNLTLPR	S3	6	3	2	2	2.00	3.00	3.00	1.50	1.50	1.00
Q13247	SRSF6	SNsPLPVPPSK	S3	5	3	2	2	1.67	2.50	2.50	1.50	1.50	1.00
Q6PD62	CTR9	HPKGEEGsDDDETENGPKPK	S8	5	2	2	2	2.50	2.50	2.50	1.00	1.00	1.00
Q969T4	UBE2E2	QRsDDESPSTSSGSSDADQR	S3	7	2	2	2	3.50	3.50	3.50	1.00	1.00	1.00
O95251	KAT7	SSGsETEQVVDFSDR	S4	8	2	2	2	4.00	4.00	4.00	1.00	1.00	1.00
P18858	LIG1	VLGsEGEEEDEALsPAK	S4; S14	4	2	2	2	2.00	2.00	2.00	1.00	1.00	1.00
P49736	MCM2	GNDPLTSsPGR	S8	4	1	2	2	4.00	2.00	2.00	0.50	0.50	1.00
P46821	MAP1B	TTsPPEVSGYSYEK	S3	5	1	2	2	5.00	2.50	2.50	0.50	0.50	1.00
Q9C0H2	TTYH3	YLATSQPRPDSSGsH	S14	4	1	1	2	4.00	4.00	2.00	1.00	0.50	0.50
Q9Y320	TMX2	AGDNIPEEQPVASTPTTVsDGENKK	S19	4	2	2	1	2.00	2.00	4.00	1.00	2.00	2.00
Q9NTI5	PDS5B	GHTASEsDEQQWPEEK	S7	4	2	2	1	2.00	2.00	4.00	1.00	2.00	2.00
Q9NYF8	BCLAF1	KETQsPEQVKSEK	S5	4	2	2	1	2.00	2.00	4.00	1.00	2.00	2.00
Q02952	AKAP12	LKETcVSGEDPTQGADLsPDEK	S18	4	2	2	1	2.00	2.00	4.00	1.00	2.00	2.00
O00567	NOP56	FSKEEPVssGPEEAVGK	S8; S9	4	1	2	1	4.00	2.00	4.00	0.50	1.00	2.00
P35613	BSG	KPEDVLDDDDAGsAPLK	S13	4	1	2	1	4.00	2.00	4.00	0.50	1.00	2.00
O15164	TRIM24	NESEDNKFsDDsDDDFVQPR	S9; S12	4	1	2	1	4.00	2.00	4.00	0.50	1.00	2.00
Q969E4	TCEAL3	EDEGEPGDEGQLEDEGsQEKQGR	S17	3	1	1	1	3.00	3.00	3.00	1.00	1.00	1.00
P49006	MARCKSL1	EGGGDSSASSPtEEEQEQGEIGAcSDE GTAQEGK	T12	3	1	1	1	3.00	3.00	3.00	1.00	1.00	1.00
Q12802	AKAP13	sGSLDSELSVSPK	S1	3	1	1	1	3.00	3.00	3.00	1.00	1.00	1.00
Q9UER7	DAXX	DGDKsPMSSLQISNEK	S5	4	0	1	1	∞	4.00	4.00	0.00	0.00	1.00

Q96RS0	TGS1	DRPHASGTDGDEsEEDPPEHKPSK	S13	3	1	0	1	3.00	∞	3.00	∞	1.00	0.00
Q9NYF8	BCLAF1	NTPSQHSHSIQHsPER	S13	3	1	0	1	3.00	∞	3.00	∞	1.00	0.00
O94762	RECQL5	YDEGsGGSGDEGRDEAHKR	S5	3	1	0	1	3.00	∞	3.00	∞	1.00	0.00
P29966	MARCKS	EELQANGSAPAADKEEPAAAGSGAAsPSAAEK	S26	3	1	1	0	3.00	3.00	∞	1.00	∞	∞
Q9C0C9	UBE2O	DHTDQTSEtAPDASVPPSVKPK	T9	3	0	0	0	∞	∞	∞	-	-	-
O94776	MTA2	DISSsLNSLADSNAR	S5	2	0	0	0	∞	∞	∞	-	-	-
Q9UQ35	SRRM2	GEFSAsPMLK	S6	3	1	1	0	3.00	3.00	∞	1.00	∞	∞
P55196	MLLT4	SSPNVANQPPsPGGK	S11	3	1	1	0	3.00	3.00	∞	1.00	∞	∞
P07814	EPRS	EYIPGQPPLSQSsDsSPTR	S13; S15	4	0	1	0	∞	4.00	∞	0.00	-	∞
Q8WUB8	PHF10	GTSDSSSGNVsEGESPPDSQEDSFQGR	S11	3	0	1	0	∞	3.00	∞	0.00	-	∞
Q96K21	ZFYVE19	LPDsDDDEDEETAIQR	S4	4	0	1	0	∞	4.00	∞	0.00	-	∞
Q9Y2U8	LEMD3	ENYsDsEEEDDDDVASSR	S4; S6	2	0	0	0	∞	∞	∞	-	-	-
P78347	GTF2I	ESTSSKsPPR	S7	2	0	0	0	∞	∞	∞	-	-	-
O15014	ZNF609	FcDsPTsDLEMR	S4; S7	2	0	0	0	∞	∞	∞	-	-	-
Q6ZR54	PPME1	GGPLGTLLANAGPSTVPVtPTAGScQPSPLSPGGSDPPPPPr	T19	3	0	0	0	∞	∞	∞	-	-	-
Q5T200	ZC3H13	GNIETTsEDGQVFsPK	S7; S14	3	0	0	0	∞	∞	∞	-	-	-
Q7Z6Z7	HUWE1	GsGTAsDDEFENLR	S2; S6	2	0	0	0	∞	∞	∞	-	-	-
Q6P6C2	ALKBH5	KSyESSEDcSEAAGSPAR	Y3	3	0	0	0	∞	∞	∞	-	-	-
O60333	KIF1B	ISDISPIGRDPSESSFsSATLTPSSTcPSLVDSR	S17	2	0	0	0	∞	∞	∞	-	-	-
O43304	SEC14L5	NQAGPRDPsSLEAHGPR	S9	2	0	0	0	∞	∞	∞	-	-	-
P07814	EPRS	NQGGGLsSSGAGEGQGPK	S7	2	0	0	0	∞	∞	∞	-	-	-
Q9Y570	PPME1	QcEGItSPEGSK	T6	2	0	0	0	∞	∞	∞	-	-	-
Q8IVH2	FOXP4	RDsSSHEETPGSHPLYGHGEcK	S3	3	0	0	0	∞	∞	∞	-	-	-
Q7Z3C6	ATG9A	REsDESGESAPDEGGEGAR	S3	3	0	0	0	∞	∞	∞	-	-	-
Q5JSH3	WDR44	SDTDTGVcSGtDEDPDDKNAPFR	T11	2	0	0	0	∞	∞	∞	-	-	-
Q5T1R4	SHN3	sLGDEEEPPAFESTK	S1	2	0	0	0	∞	∞	∞	-	-	-
P18615	NELFE	sLSEQPVMDTATATEQAK	S1	2	0	0	0	∞	∞	∞	-	-	-
P29218	IMPA1	SsPVDLVTATDQK	S2	2	0	0	0	∞	∞	∞	-	-	-
Q8TEP8	CEP192	StSDLDKDDASYLR	T2	3	0	0	0	∞	∞	∞	-	-	-
Q15648	MED1	SYQNsPsSDDGIRPLPEYSTEK	S5; S7	2	0	0	0	∞	∞	∞	-	-	-
Q6P4E1	CASC4	VAENVADKNEEPsSNHIPHGK	S13	2	0	0	0	∞	∞	∞	-	-	-

Supplementary Table S4. Potential targets of NF-κB regulated by PTP1B and IL-13

Proteins regulated by IL13 PTP1B		ENCODE	SWISS REGULON
Q12802	AKAP13	Yes	Yes
Q6P6C2	ALKBH5	Yes	Yes
Q07960	ARHGAP1	Yes	Yes
Q7Z3C6	ATG9A	Yes	No
Q9NYF8	BCLAF1	Yes	No
P35613	BSG	Yes	Yes
Q6P4E1	CASC4	Yes	No
Q8TEP8	CEP192	Yes	No
Q6PD62	CTR9	Yes	No
Q8TEA8	DTD1	Yes	No
P07814	EPRS	Yes	No
Q8IVH2	FOXP4	Yes	Yes
P78347	GTF2I	Yes	Yes
P29218	IMPA1	Yes	No
O95251	KAT7	Yes	No
O60333	KIF1B	Yes	No
Q9Y2U8	LEMD3	Yes	No
P18858	LIG1	Yes	No
P29966	MARCKS	Yes	No
P49006	MARCKSL1	Yes	Yes
P49736	MCM2	Yes	No
Q15648	MED1	Yes	No
O94776	MTA2	Yes	No
O00567	NOP56	Yes	No
Q9NTI5	PDS5B	Yes	No
Q6ZR54	PPME1	Yes	No
O94762	RECQL5	Yes	No
Q8IYB3	SRRM1	Yes	Yes
Q9UQ35	SRRM2	Yes	No
Q13247	SRSF6	Yes	No
Q99081	TCF12	Yes	Yes
Q96RS0	TGS1	Yes	No
Q9Y320	TMX2	Yes	No
O15164	TRIM24	Yes	No
Q9C0H2	TTYH3	Yes	No
Q969T4	UBE2E2	Yes	No
Q96K21	ZFYVE19	Yes	No
