# Supplementary material for: Schnurri-3 drives tumor growth and invasion in cancer cells expressing interleukin-13 receptor alpha 2
Source: Cell Death Dis. 2023 Nov 14;14(11):742. doi: 10.1038/s41419-023-06255-4 (PMC10645886; doi:10.1038/s41419-023-06255-4)
Supplement: Supplementary file 3 — Original western blots [file 41419_2023_6255_MOESM3_ESM.pdf]

Figure 1D

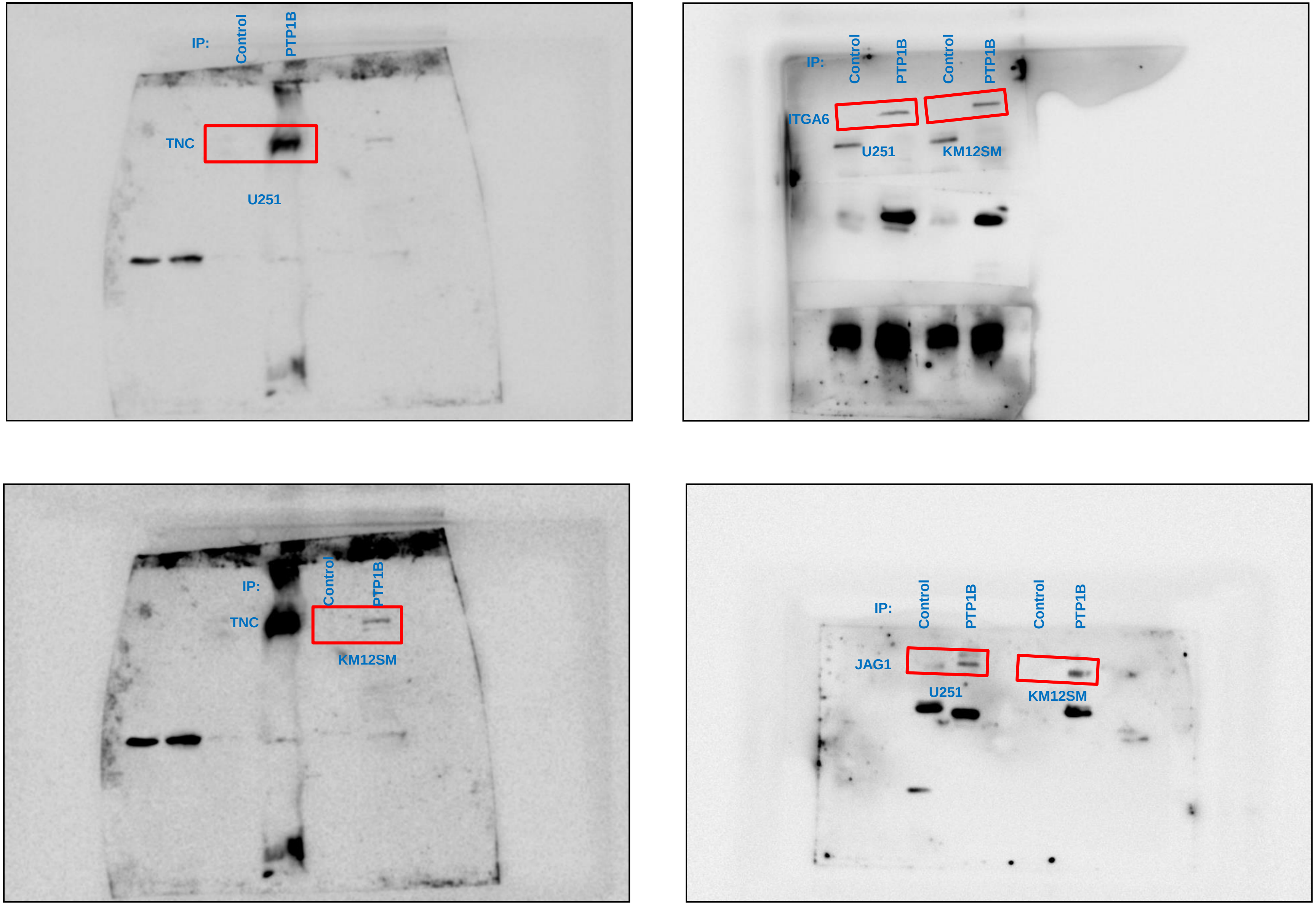
IP:
Control
PTP1B
TNC
U251
IP:
Control
PTP1B
Control
PTP1B
ITGA6
U251
KM12SM
IP:
Control
PTP1B
TNC
KM12SM
IP:
Control
PTP1B
Control
PTP1B
JAG1
U251
KM12SM

Figure 1D (continuation)

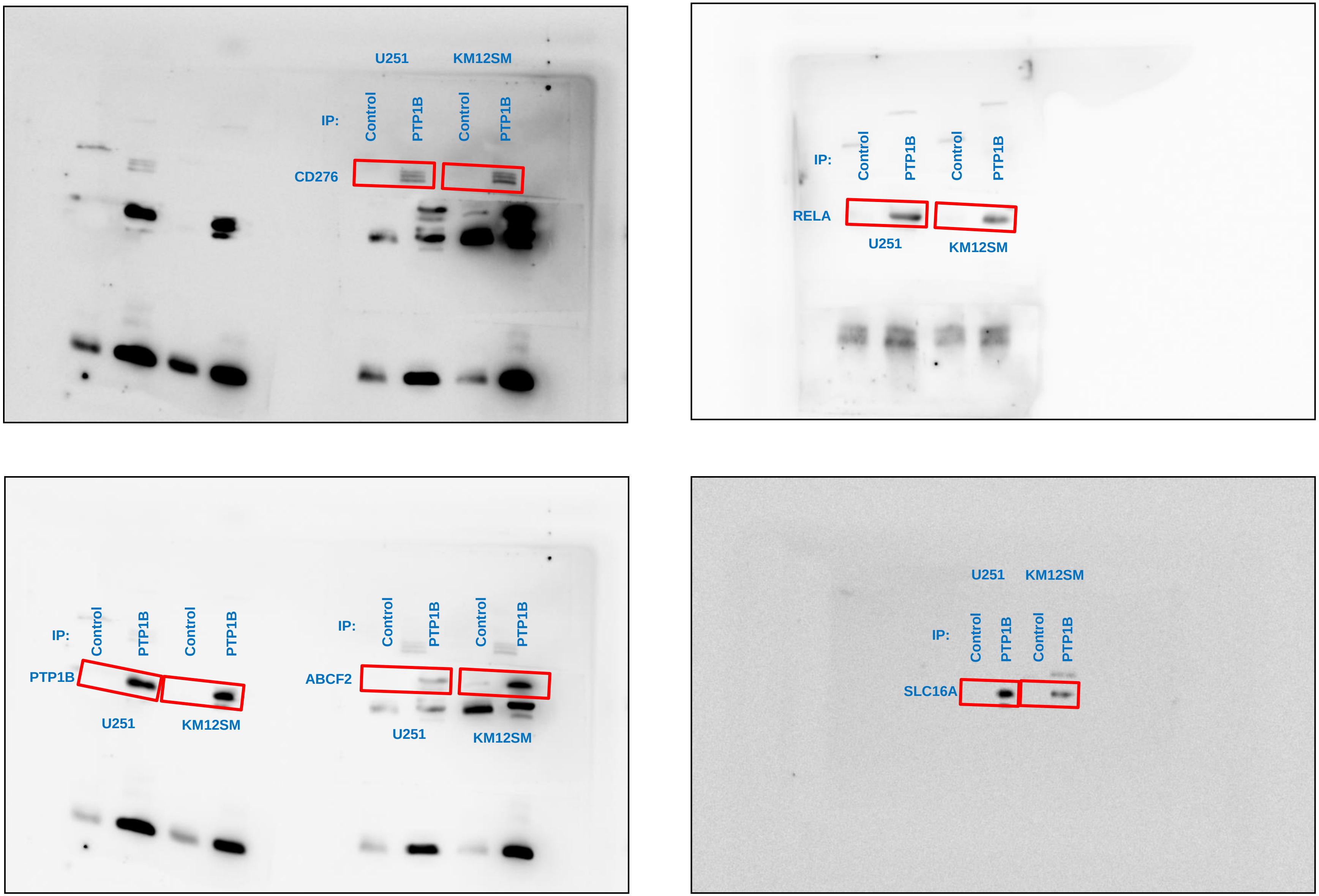

Figure 1D (continuation)

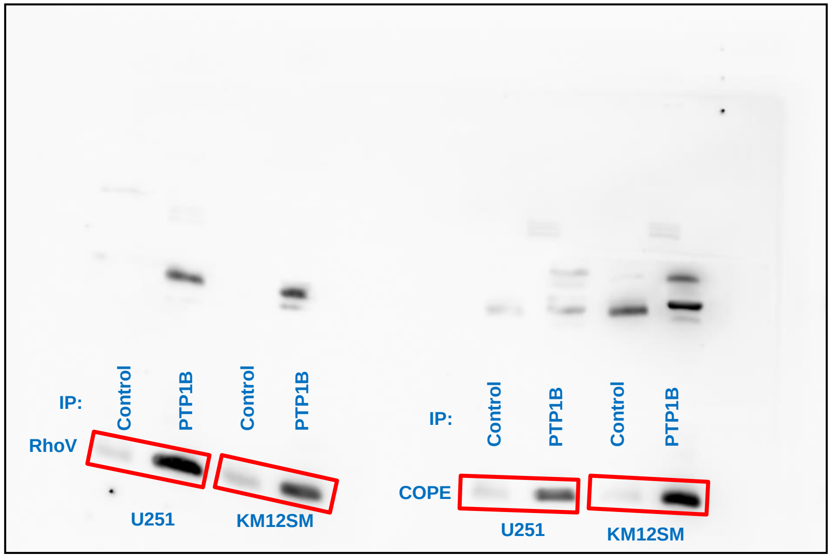

Figure 2A

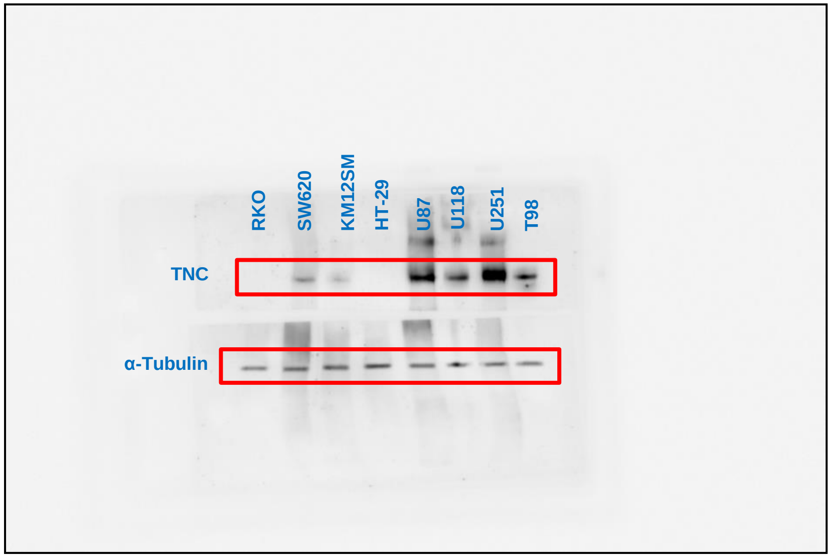

Figure 3E

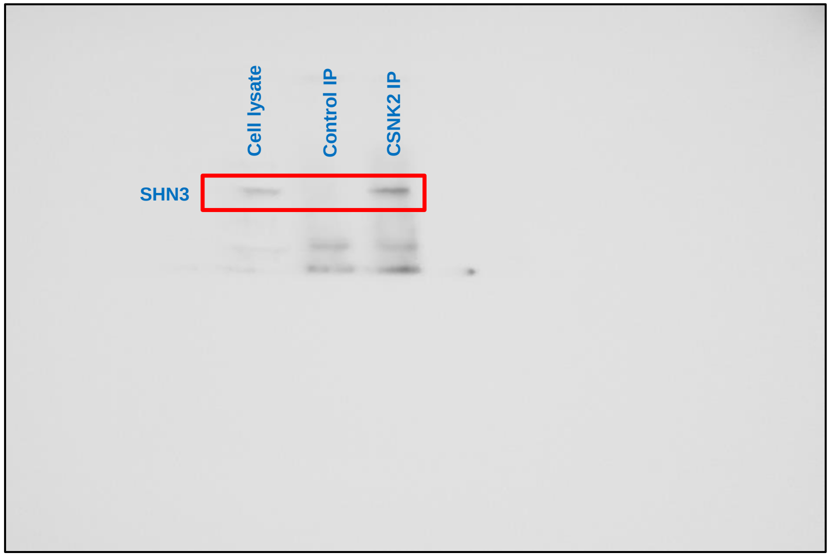
Cell lysate
Control IP
CSNK2 IP
SHN3

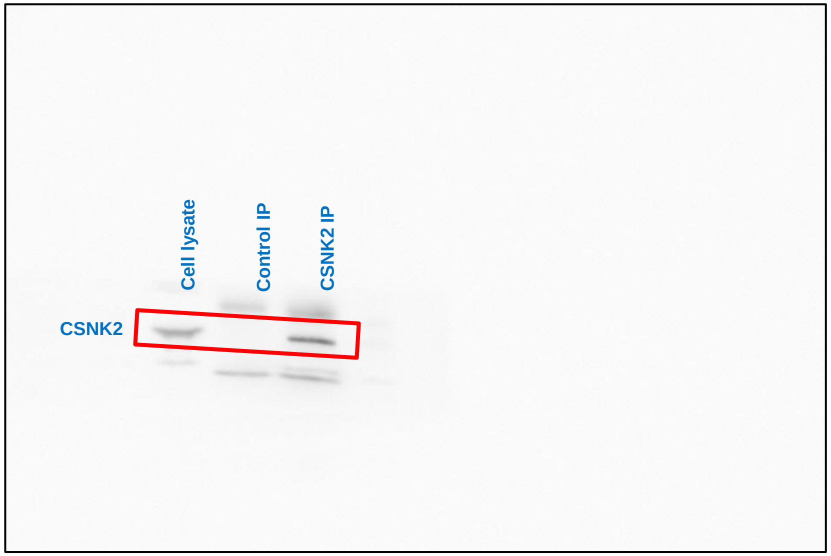
Cell lysate
Control IP
CSNK2 IP
CSNK2

Figure 4A

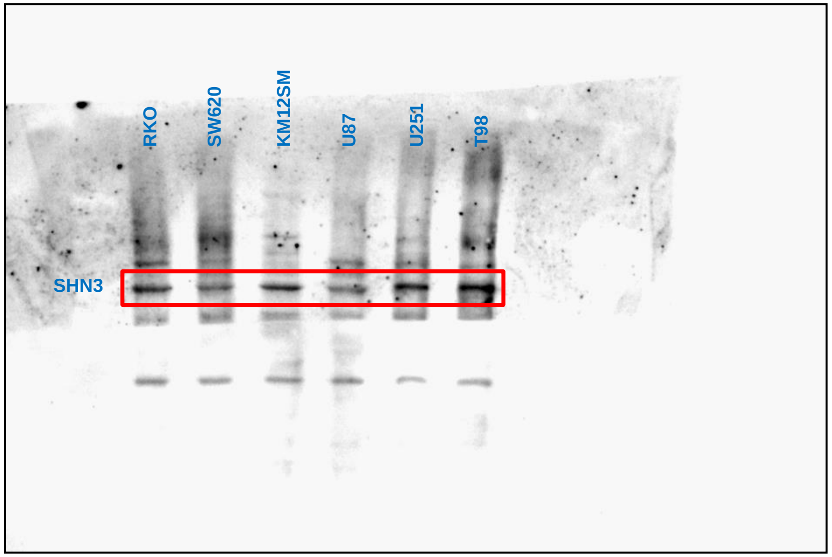
RKO
SW620
KM12SM
U87
U251
T98
SHN3

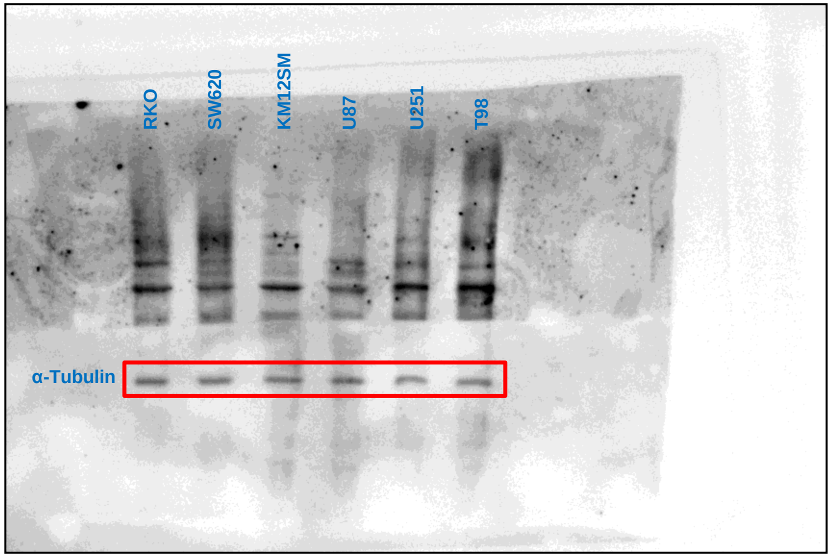
RKO
SW620
KM12SM
U87
U251
T98
α-Tubulin

Figure 4B

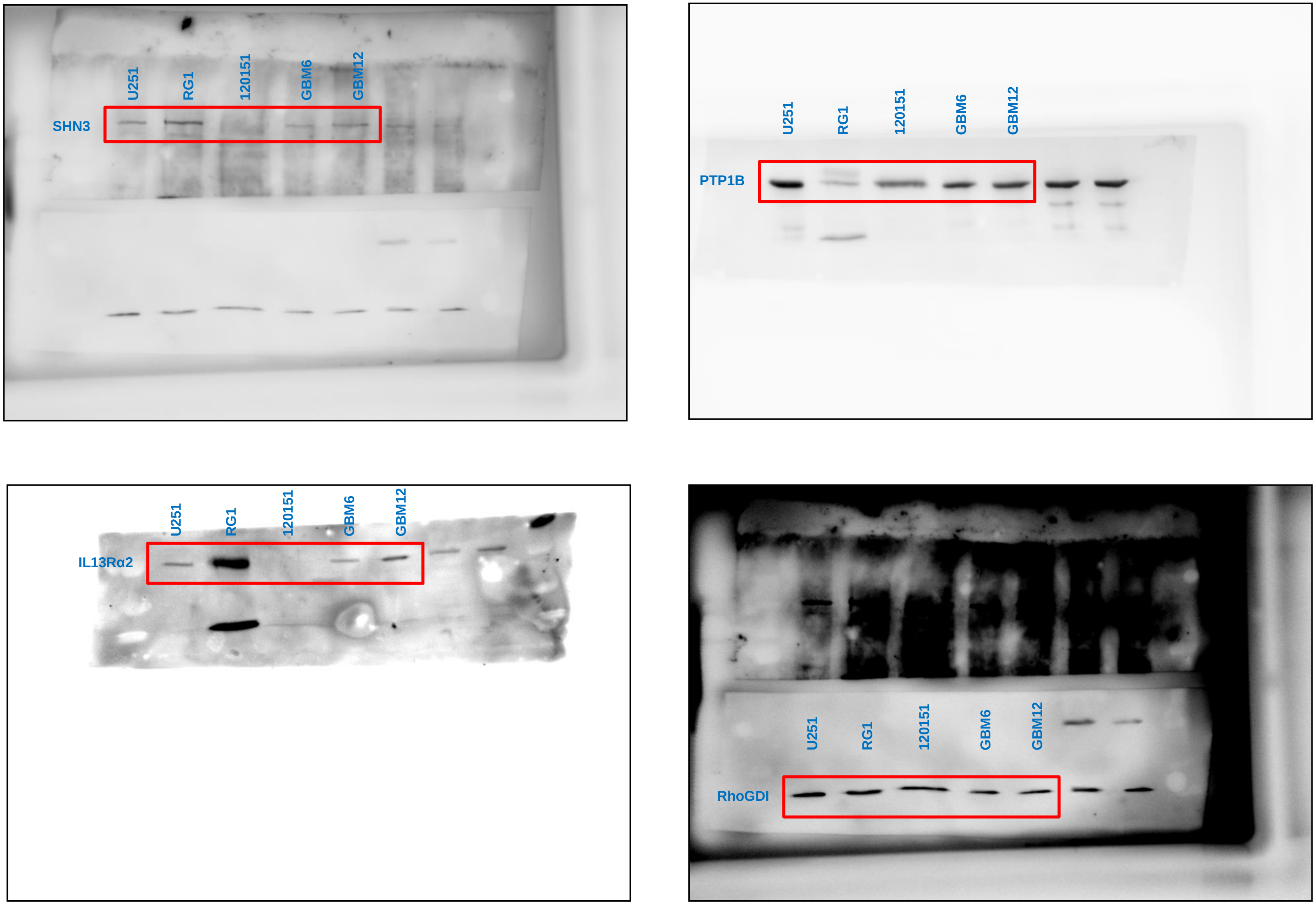

Figure 4D

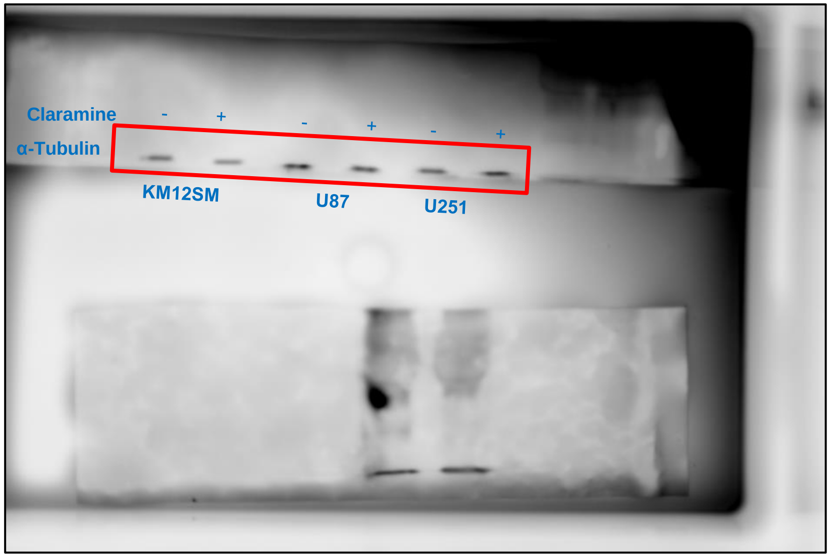

Claramine
-
+
-
+
-
+
α-Tubulin
KM12SM
U87
U251

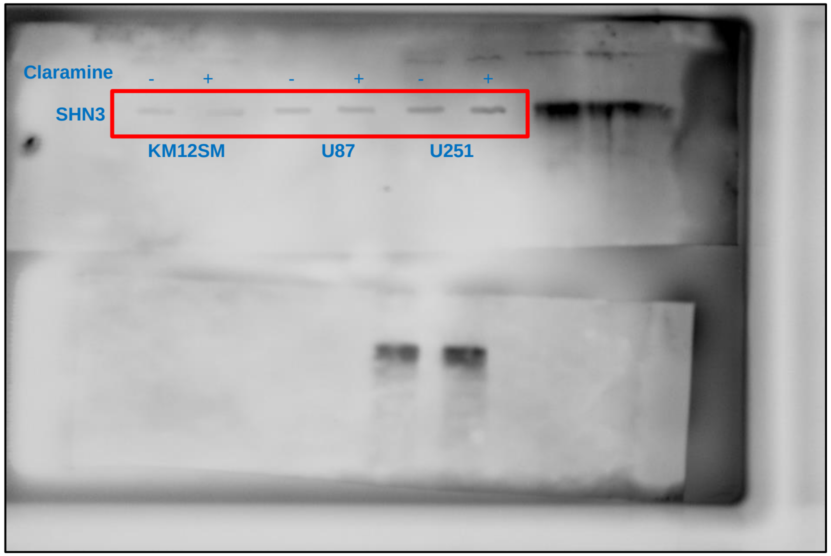

Claramine
-
+
-
+
-
+
SHN3
KM12SM
U87
U251

Figure 4E

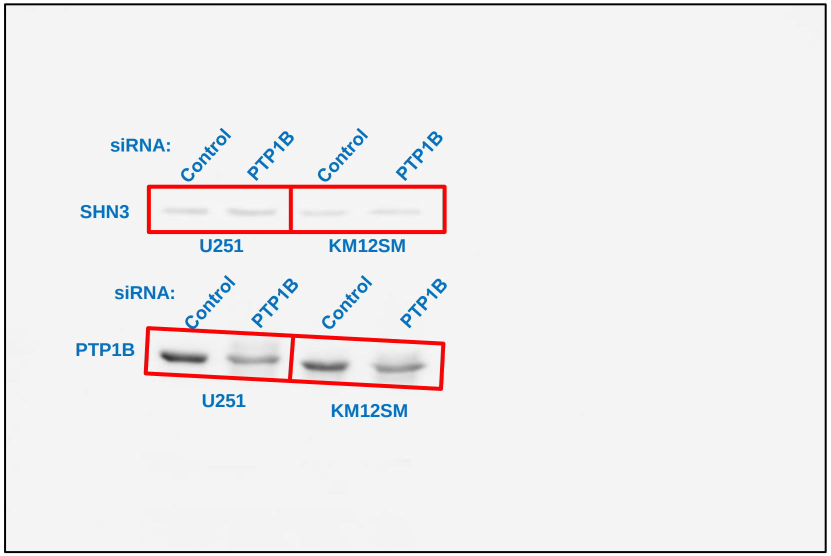

siRNA:
Control
PTP1B
Control
PTP1B
SHN3
U251
KM12SM
siRNA:
Control
PTP1B
Control
PTP1B
PTP1B
U251
KM12SM

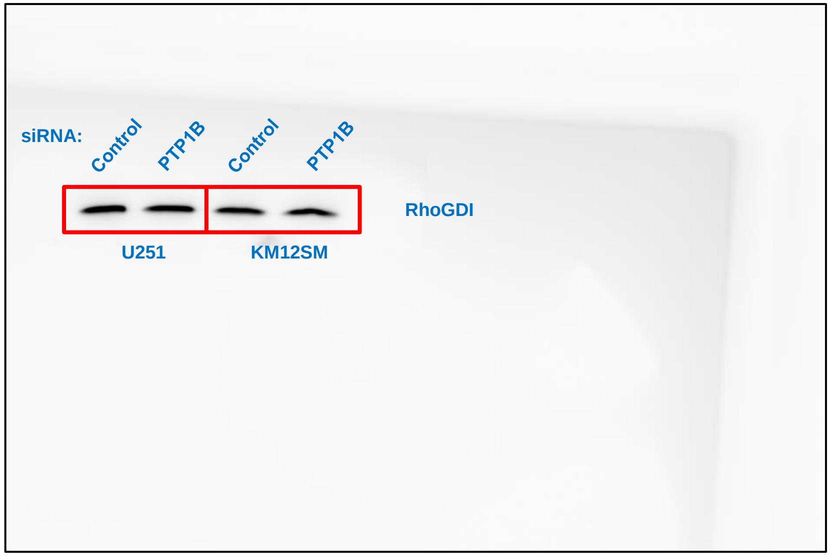

siRNA:
Control
PTP1B
Control
PTP1B
RhoGDI
U251
KM12SM

Figure 6A

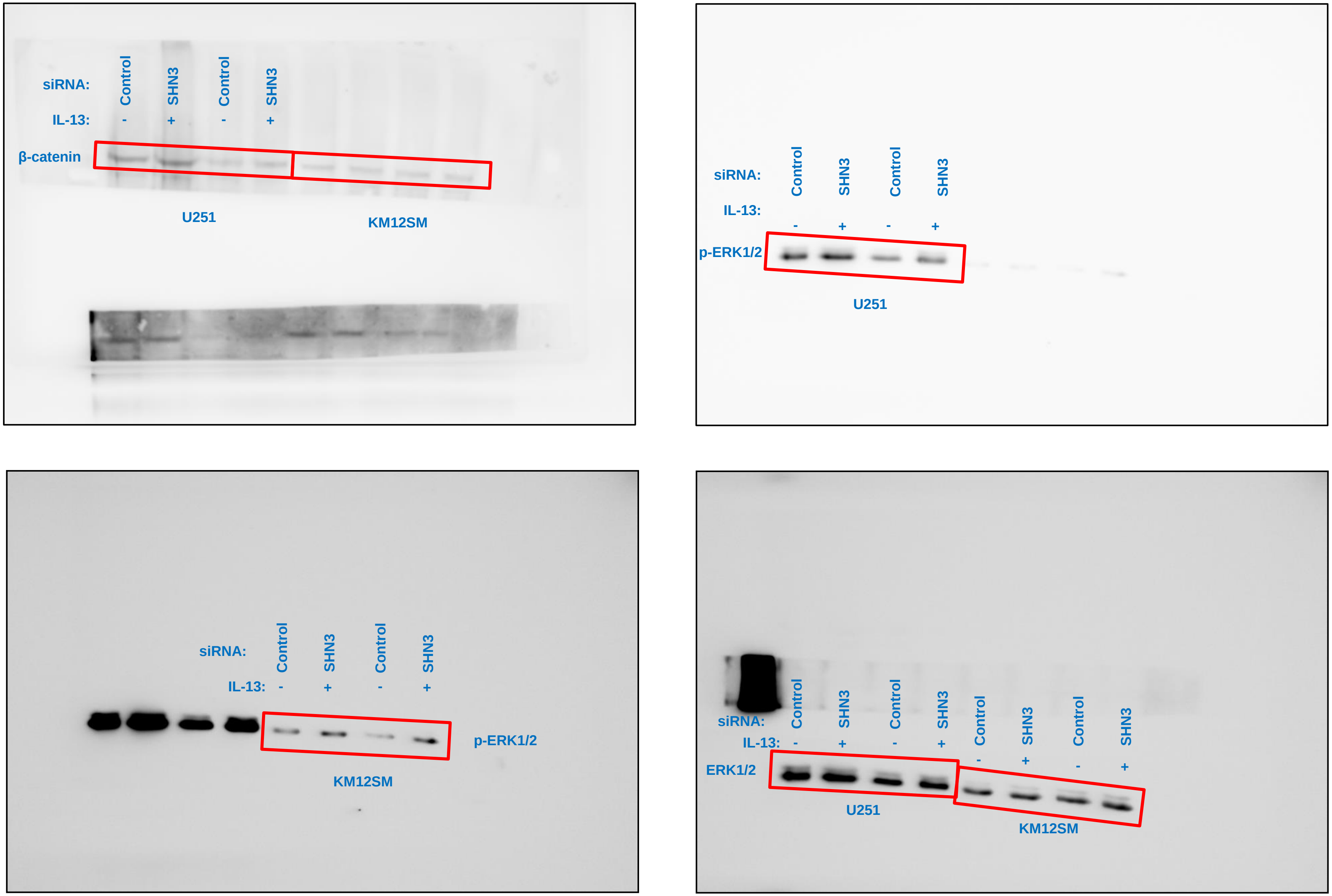

siRNA:
Control
SHN3
Control
SHN3
IL-13:
-
+
-
+
β-catenin
U251
KM12SM
siRNA:
Control
SHN3
Control
SHN3
IL-13:
-
+
-
+
p-ERK1/2
U251
siRNA:
Control
SHN3
Control
SHN3
IL-13:
-
+
-
+
p-ERK1/2
KM12SM
siRNA:
Control
SHN3
Control
SHN3
Control
SHN3
Control
SHN3
IL-13:
-
+
-
+
-
+
-
+
ERK1/2
U251
KM12SM

Figure 6A (continuation)

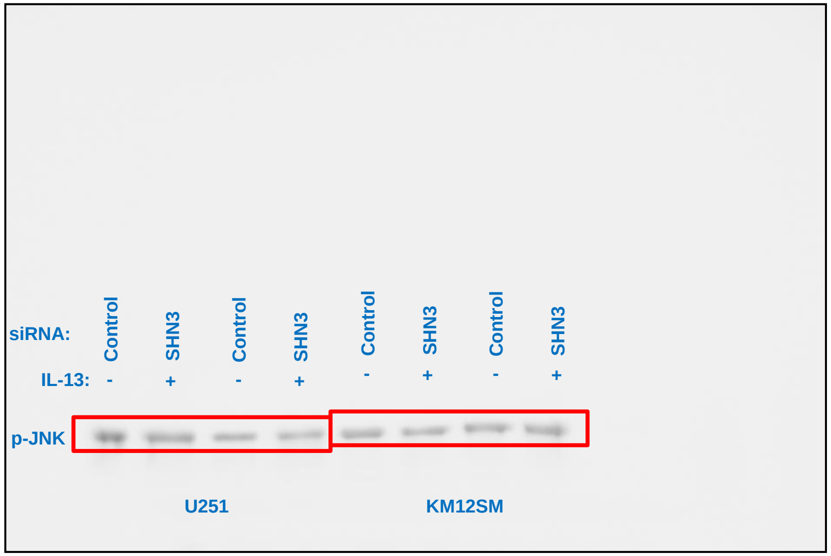

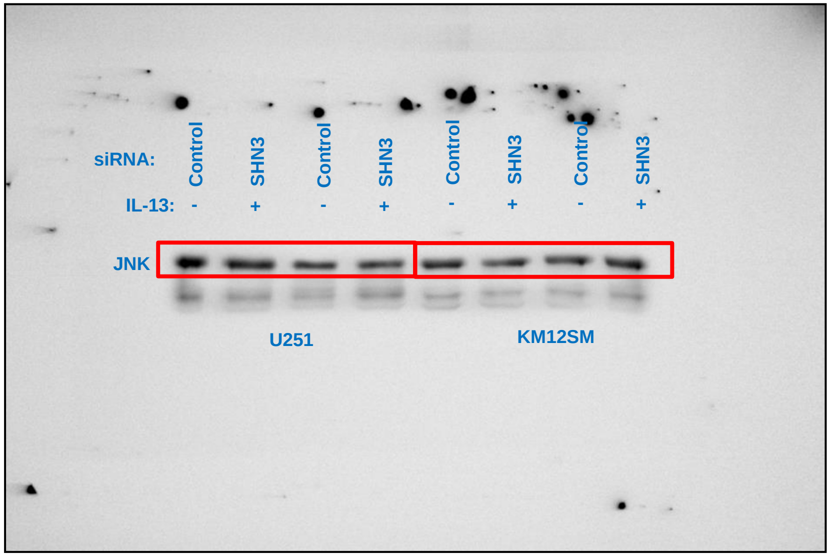

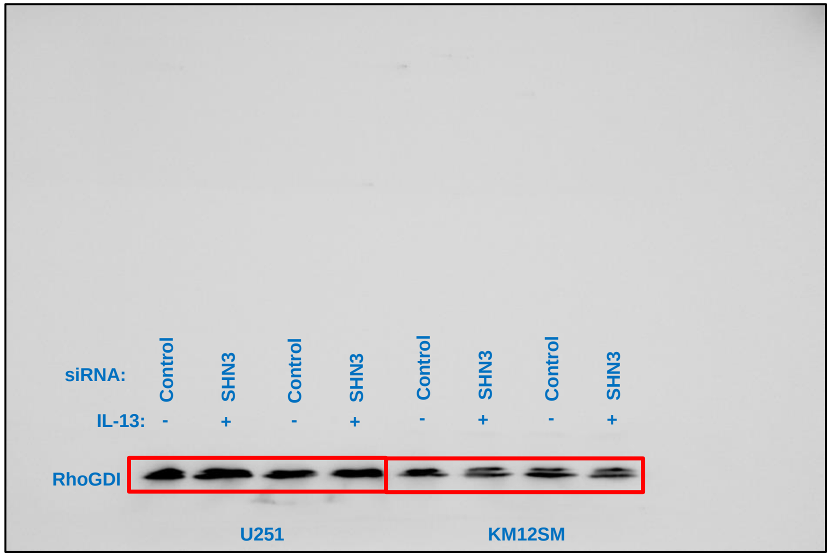

Figure 6D

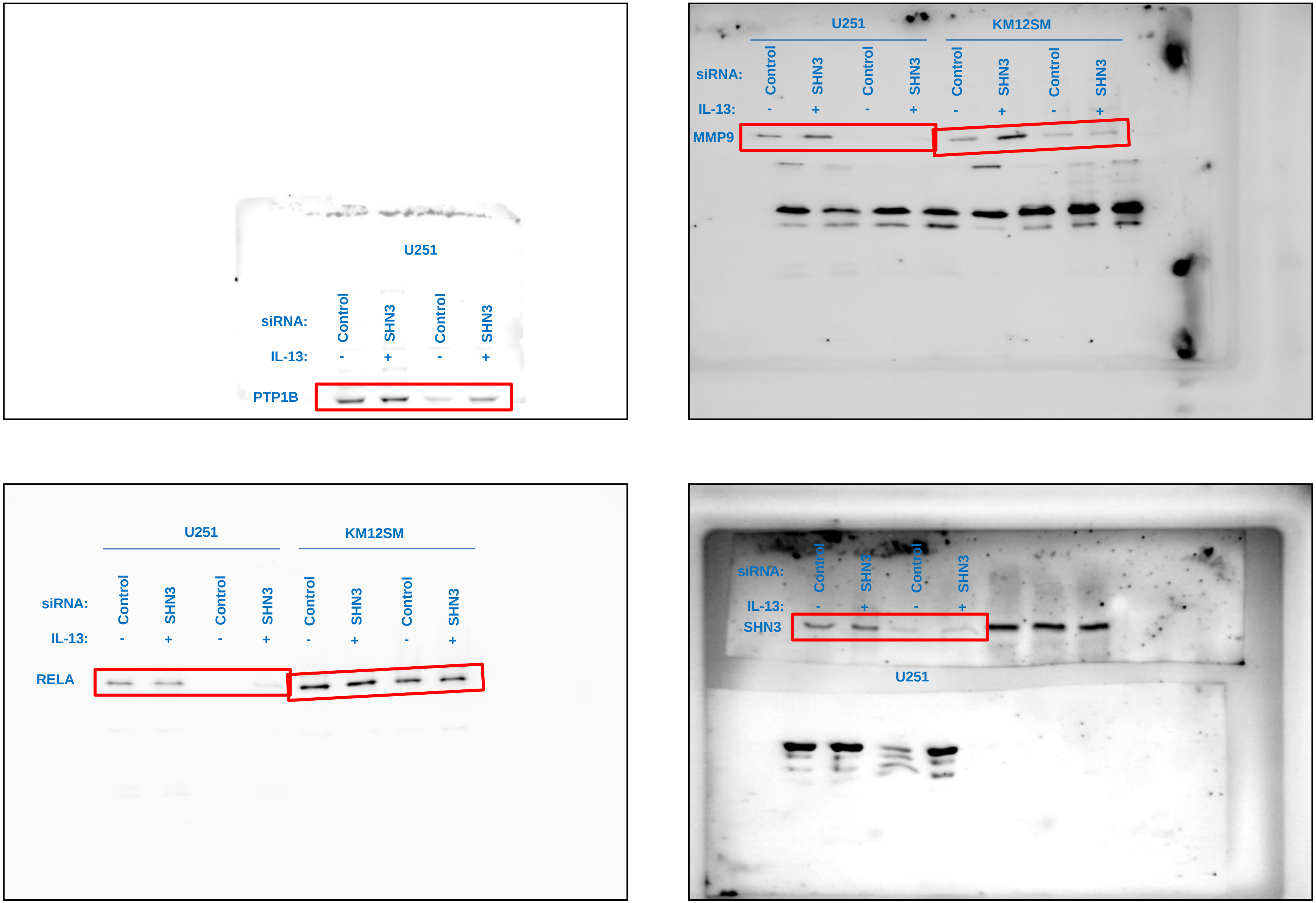
U251
siRNA:
Control
SHN3
Control
SHN3
IL-13:
-
+
-
+
PTP1B
U251
KM12SM
siRNA:
Control
SHN3
Control
SHN3
Control
SHN3
Control
SHN3
IL-13:
-
+
-
+
-
+
-
+
MMP9
U251
KM12SM
siRNA:
Control
SHN3
Control
SHN3
Control
SHN3
Control
SHN3
IL-13:
-
+
-
+
-
+
-
+
RELA
siRNA:
Control
SHN3
Control
SHN3
IL-13:
-
+
-
+
SHN3
U251

Figure 6D (continuation)

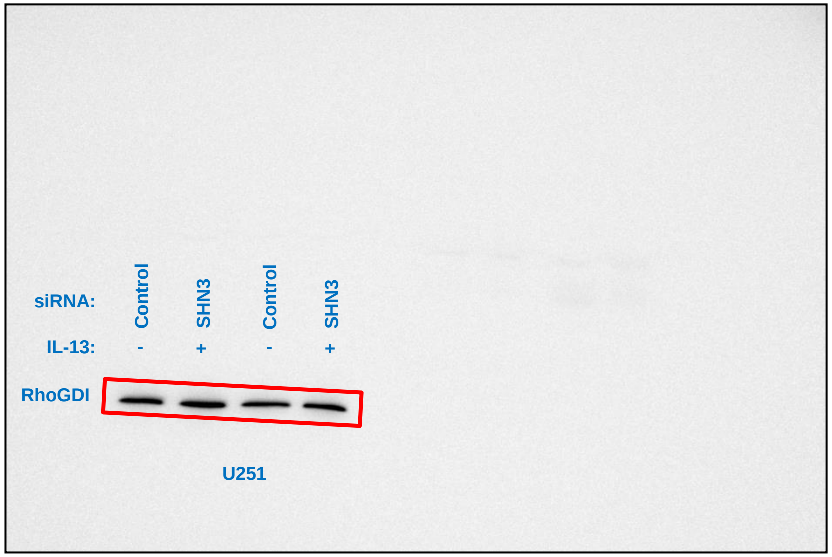

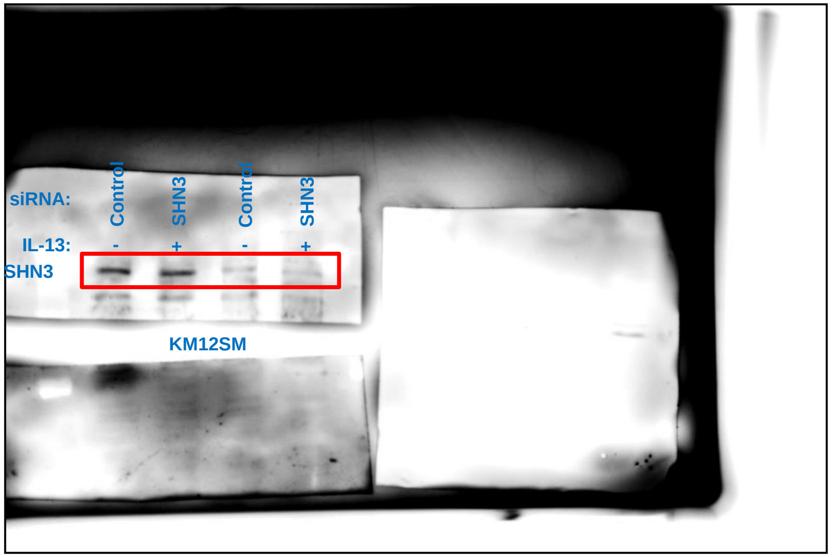

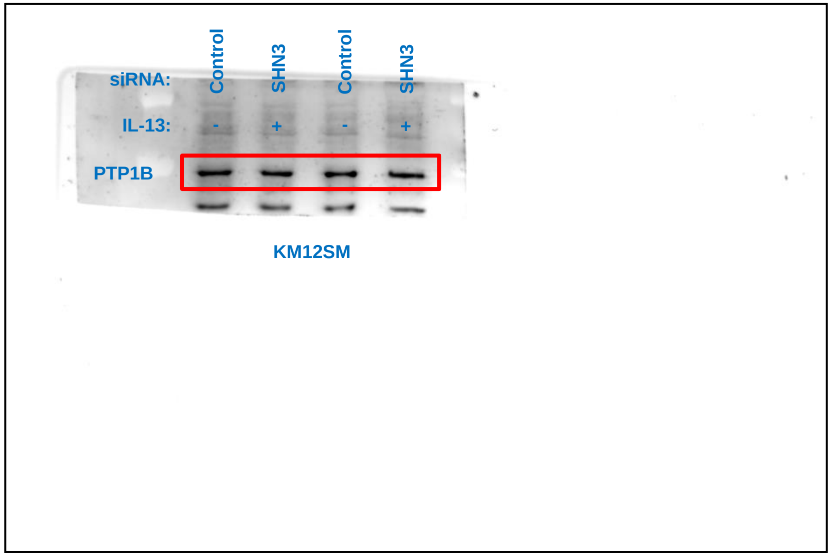

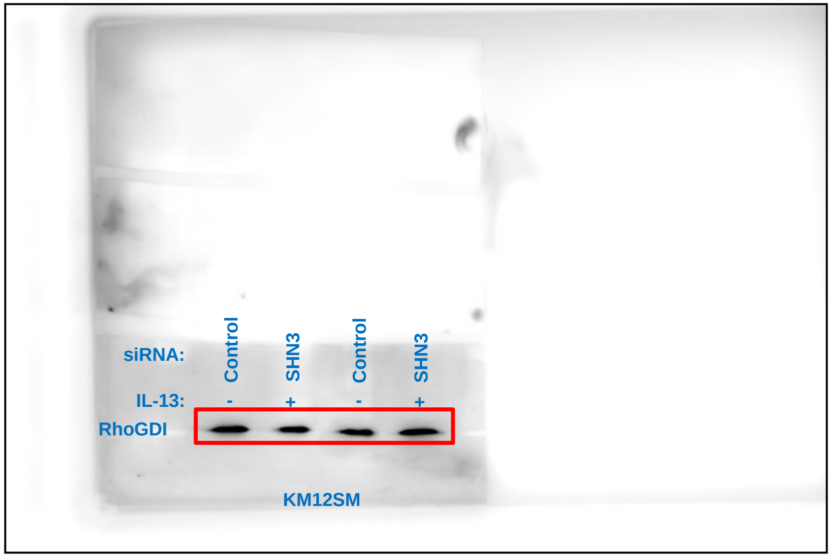

Figure S3C

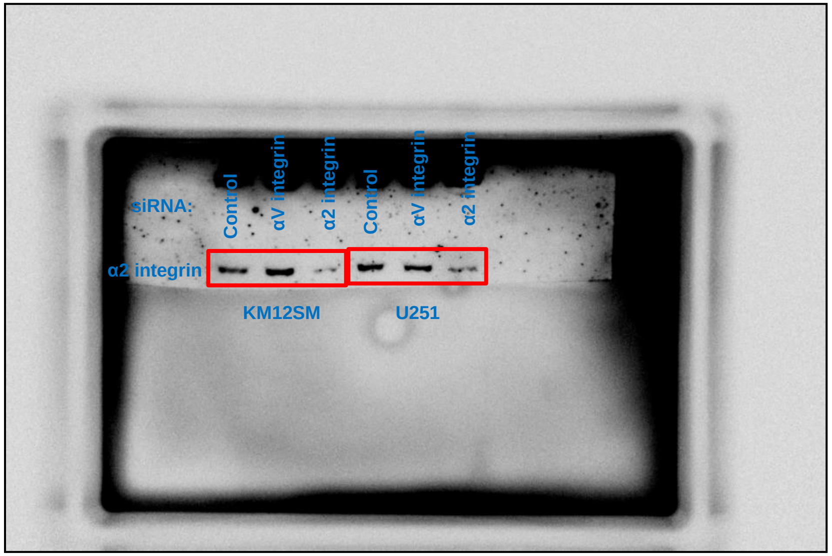
siRNA:
Control
αV integrin
α2 integrin
Control
αV integrin
α2 integrin
α2 integrin
KM12SM
U251

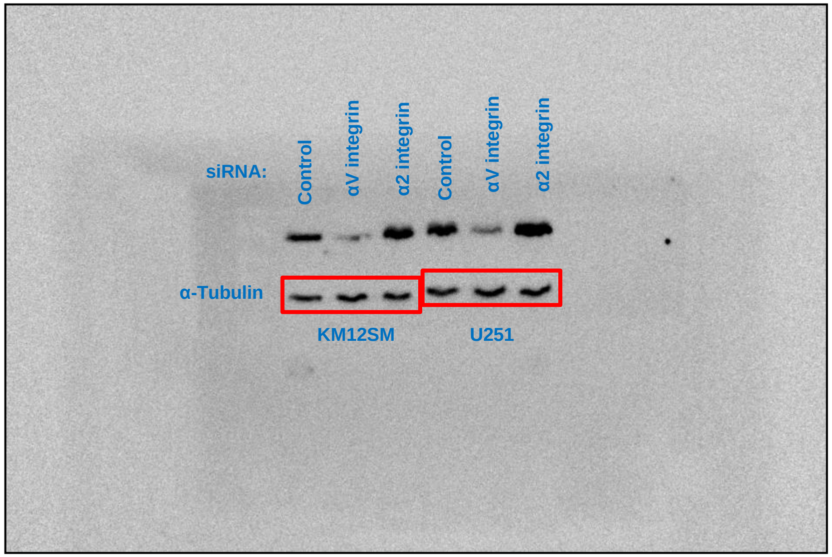
siRNA:
Control
αV integrin
α2 integrin
Control
αV integrin
α2 integrin
α-Tubulin
KM12SM
U251

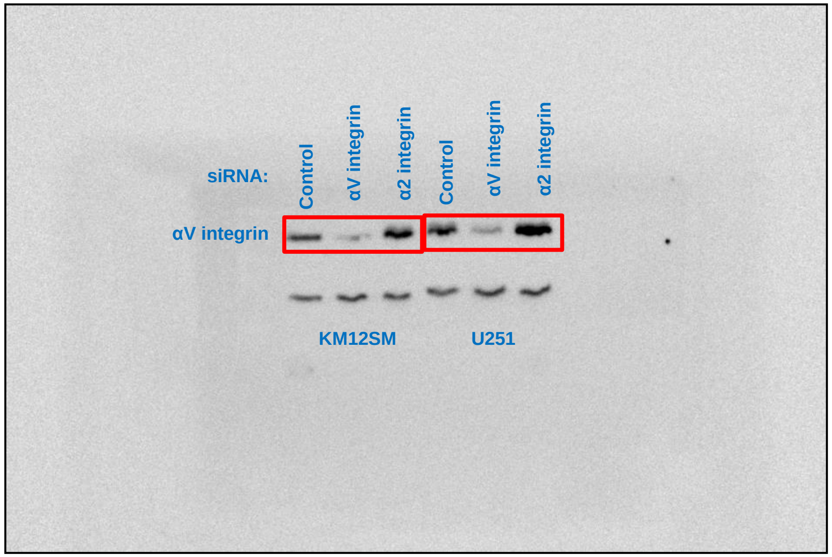
siRNA:
Control
αV integrin
α2 integrin
Control
αV integrin
α2 integrin
αV integrin
KM12SM
U251

Figure S5A

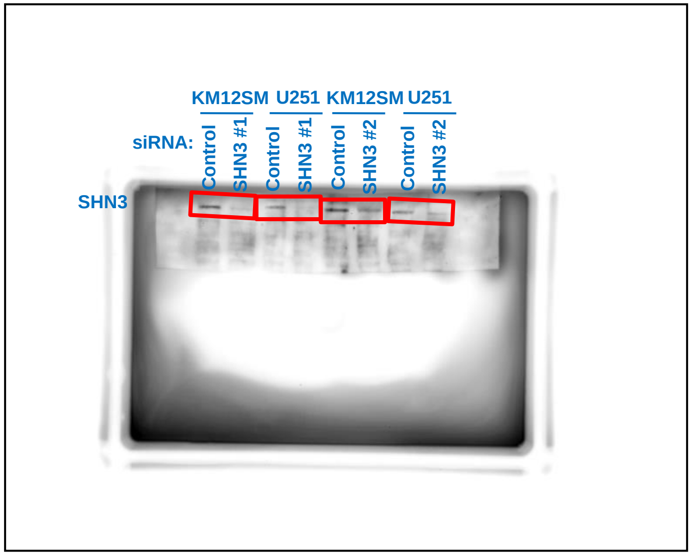

KM12SM
U251
KM12SM
U251
siRNA:
Control
SHN3 #1
Control
SHN3 #1
Control
SHN3 #2
Control
SHN3 #2
SHN3

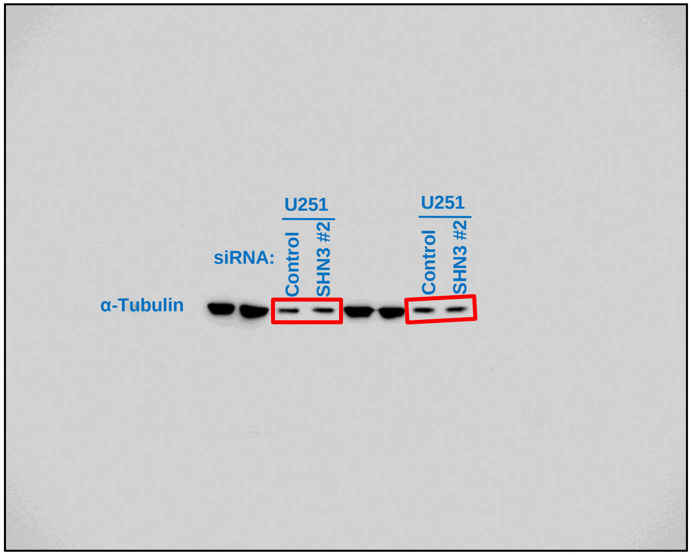

U251
U251
siRNA:
Control
SHN3 #2
Control
SHN3 #2
α-Tubulin

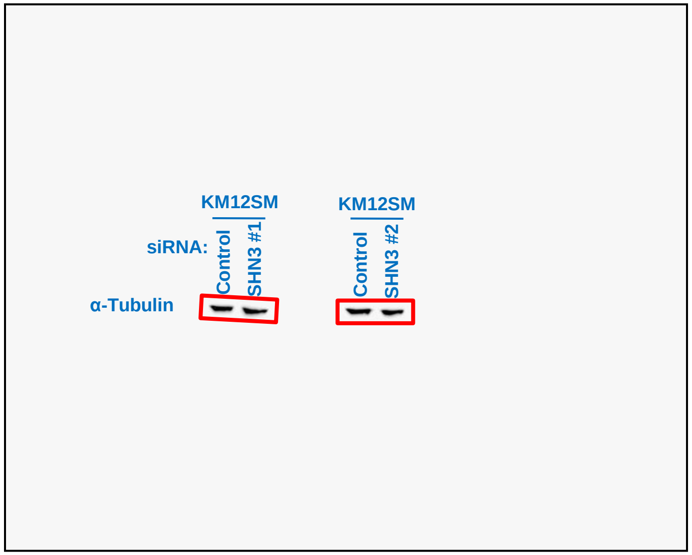

KM12SM
KM12SM
siRNA:
Control
SHN3 #1
Control
SHN3 #2
α-Tubulin
